# Dahuang gancao dandelion decoction regulates intestinal flora and inhibits NF-κB/ARA signaling pathway to alleviate ulcerative colitis

**DOI:** 10.3389/fimmu.2025.1735021

**Published:** 2026-01-29

**Authors:** Xieraili Malajiang, Wan er Shen, Alimu Aersilan, Mengzi Liu, Yuan Liang, Shengyi Wang, Saifuding Abula, Adelijiang Wusiman

**Affiliations:** 1 College of Veterinary Medicine, Xinjiang Agricultural University, Urumqi, China; 2 Xinjiang Key Laboratory of New Drug Study and Creation for Herbivorous Animal (XJ-KLNDSCHA), Xinjiang Agricultural University, Urumqi, China; 3 College of School of Public Administration (Law School), Xinjiang Agricultural University, Urumqi, China; 4 Changji University, Changji, China; 5 Lanzhou Institute of Husbandry and Pharmaceutical Sciences of Chinese Academy of Agricultural Sciences, Lanzhou, China

**Keywords:** dahuang gancao dandelion decoction, gutmicrobiota, intestinal barrier, NF-κB/ARA signaling pathway, traditional chinese medicine, ulcerative colitis

## Abstract

**Objective:**

The aim of this study was to assess the therapeutic effects and underlying mechanisms of Dahuang Gancao Dandelion Decoction (DGD-D) on dextran sulfate sodium (DSS)-induced ulcerative colitis (UC) in a mice model, with an emphasis on modulation of gut microbiota and intestinal metabolites, maintenance of intestinal barrier integrity, and inhibition of inflammatory signaling pathways.

**Methods:**

Ultra-high-performance liquid chromatography with quadrupole electrospray ionization mass spectrometry (UHPLC-QE-MS) was used to examine the DGD-D, which was prepared from 40 g rhubarb, 10 g licorice, and 10 g dandelion. Potential routes and targets were found using network pharmacology. Six male C57BL/6 mice per group were randomized into control, DSS, DGD-D, and mesalazine (5-ASA) groups. UC was induced with 3% DSS for 7 days, with DGD-D administered prophylactically at 1950 mg/kg. Evaluated parameters included colon length, spleen index, and DAI, cytokine levels (ELISA, qRT-PCR), histological changes (H&E, PAS, AB-PAS), barrier proteins (IF, IHC, qRT-PCR), gut microbiota (16S rRNA sequencing), metabolites (LC-MS) and pathway validation.

**Results:**

UHPLC-QE-MS identified 418 components in DGD-D, including flavonoids (25.12%) and phenolic acids (9.33%). Network pharmacology highlighted NF-κB signaling as a key pathway. In comparison to DSS, DGD-D dramatically decreased spleen index, restored colon length, and decreased DAI scores (*P* < 0.05). It increased anti-inflammatory IL-4 and IL-10 while downregulating pro-inflammatory cytokines (IL-1β, IL-6, IL-17, TNF-α, and IFN-γ) and MPO (*P* < 0.01). Histologically, DGD-D attenuated intestinal epithelial damage, increased goblet cells and glycoproteins, and enhanced ZO-1, Occludin, and MUC2 expression (*P* < 0.01). Microbiota analysis showed increased α-diversity, elevated *Firmicutes/Bacteroidetes* ratio, enriched beneficial *Lachnospiraceae* and *Ruminococcaceae*, and reduced *Proteobacteria* and *Alcaligenaceae*, *Moraxellaceae* and *Xanthomonadaceae* (*P* < 0.05). Metabolomics revealed downregulation of arachidonic acid (ARA) pathway mediators (ARA, LTA4, LTB4, LTD4; *P* < 0.001). DGD-D inhibited TLR4/MyD88/NF-κB p65/NLRP3/5-LOX expression (*P* < 0.01 or *P* < 0.001), suppressing NF-κB/ARA signaling.

**Conclusion:**

DGD-D is a viable TCM-based treatment for UC since it improves DSS-induced UC by modifying gut microbiota and intestinal metabolites, reestablishing intestinal barrier function, and blocking the NF-κB/ARA pathway.

## 1 Introduction

Ulcerative colitis (UC), a relapsing-remitting idiopathic inflammatory bowel disease, is defined by persistent superficial intestinal epithelial inflammation and crypt abscesses commencing at the rectosigmoid junction and extending proximally to varying degrees, conferring an elevated risk of colorectal dysplasia and adenocarcinoma, with hallmark clinical features encompassing urgency, bloody mucoid diarrhea, and crampy lower abdominal pain (1). UC is a significant health burden, with a global prevalence expected to surpass 5 million cases by 2023, involving 1.5 million cases in North America and 2 million cases in Europe (2, 3). This condition is believed to arise from the interplay between genetic predisposition and environmental influences, leading to dysregulation of gut microbiota and metabolic profiles, which in turn promote disruption of the intestinal barrier and initiate immune dysfunction in the gut (4). The healthy gut microbiota is made up of over 90% of Firmicutes, Bacteroidetes, and Proteobacteria (4). It is well known that alterations in the composition of the gut microbiota community (e.g. a decrease in Firmicutes and an increased in Proteobacteria), disrupt the intestinal milieu and promote the activation of several pro-inflammatory signaling cascades, particularly the TLR4, MYD88, and NF-κB activation (5). Meanwhile, studies have demonstrated significant differences in intestinal metabolites—including bile acid metabolites, tryptophan metabolites, and arachidonic acid (ARA) metabolites—between patients with UC and healthy people (6, 7). Among these, inflammatory metabolites such as ARA abnormally elevate within the body, promoting the degradation of key proteins in the intestinal barrier (e.g., ZO-1, Occludin, MUC2), thus it leads to ulcerative colitis (8). Therefore, modulating the gut microbiota and metabolic profiles to restore intestinal barrier integrity represents an effective therapeutic strategy for the management of ulcerative colitis. Current therapies, such as 5-aminosalicylic acid and biologics, provide limited efficacy and are associated with significant side effects or reduced effectiveness over time, underscoring the need for safer, more effective treatments (9).

Traditional Chinese medicine (TCM) offers a multi-target approach to managing gastrointestinal disorders by modulating the intestinal microenvironment and immune responses (10). Dahuang Gancao Decoction (DGD), a classical TCM formula comprising rhubarb (Rheum palmatum L.) and licorice (Glycyrrhiza uralensis Fisch.), is documented for its heat-clearing, anti-inflammatory, and antibacterial effects, particularly in treating damp-heat dysentery (11, 12). Rhubarb provides relieving diarrhea and detoxifying benefits, while licorice enhances formula synergy and analgesia (12, 13). Dandelion (Taraxacum mongolicum Hand.-Mazz.), a potent TCM herb, exhibits anti-inflammatory and antidiarrheal properties, with its polysaccharides shown to increase Firmicutes abundance and support intestinal barrier integrity (14).

Based on these pharmacological properties, we hypothesized that incorporating dandelion into Dahuang Gancao Decoction would further enhance its therapeutic potential against UC. As a result, we formulated Dahuang Gancao Dandelion Decoction (DGD-D) using rhubarb, licorice, and dandelion, and examined its therapeutic effectiveness in a mice model caused by DSS. Employed network pharmacology to evaluate intestinal barrier function, the gut microbiota and metabolites, thereby elucidating the therapeutic efficacy and mechanism of action of DGD-D. In order to promote DGD-D as a viable TCM-based therapy alternative for the treatment of UC.

## 2 Materials and methods

### 2.1 Preparation of DGD-D

Dahuang Gancao Dandelion Decoction (DGD-D) was prepared using 40 g rhubarb (Rheum palmatum L.), 10 g licorice (Glycyrrhiza uralensis Fisch.), and 10 g dandelion (Taraxacum mongolicum Hand.-Mazz.) sourced from Ailikan Herbal Store (Urumqi, Xinjiang, China). Herbs were authenticated by botanical experts at Xinjiang Agricultural University and voucher specimens (No. XJAU2022-001–003) were deposited at the university’s herbarium. The dried herbs were decocted in 20 volumes of distilled water for1.5 h, after which a second extraction in 15 volumes of water for 1 h. The mixed filtrates were concentrated to 0.15 g/mL. The UHPLC-QE-MS was used to measure the chemical composition of them.

### 2.2 Network pharmacology analysis

Bioactive constituents of DGD-D were identified through UHPLC-MS/MS analysis. The compounds were retrieved from the TCM System databases such as TCMSP and HERB based on the following criteria: bioavailability (OB) ≥ 30%, drug-likeness (DL) ≥ 0.18, and relevance to inflammation treatment. SwissTargetPrediction (http://swisstargetprediction.ch/, probability > 0.01), BATMAN-TCM (score ≥ 80), and TargetNet (http://targetnet.scbdd.com, probability ≥ 70%) were used to predict potential targets. Targets related to UC were obtained from GeneCards, DrugBank, OpenTargets, and the Comparative Toxicogenomics Database. Overlapping targets were illustrated through Venn diagrams generated using Microbioinformatics tools (https://www.bioinformatics.com.cn/). Compound-target-disease interaction networks were constructed with Cytoscape v3.9.1 (https://cytoscape.org/), whilst analysing protein-protein interactions (PPIs) using String (https://stringdb.org/). Finally, the relevant targets of DGD - D and UC were subjected to KEGG pathway enrichment analysis and GO functional enrichment analysis.

### 2.3 Animal experiment

The Animal Welfare and Ethics Committee examined and permitted each experimental protocol (No. 2022016). The mice (C57BL/6) (Wuhan, China) were 6–8 weeks old and weighed 20–24 g.

The experiment was divided into two parts. In Part I, following a week of acclimatization, 32 mice (half male and half female) were divided into four groups at random: control (CK), LOW (950mg/kg), Mediue (1450mg/kg), High (1950mg/kg). During the 0–7 days of the experiment, Mice were euthanized 24 hours after the last administration under anesthesia induced by intraperitoneal injection of 1.25% avertin (20 mL/kg; Nanjing Aibei Biotechnology Co., Ltd). Then, samples of liver, spleen, kidney and small intestine tissues were collected for drug safety experiments.

**Figure 1:**
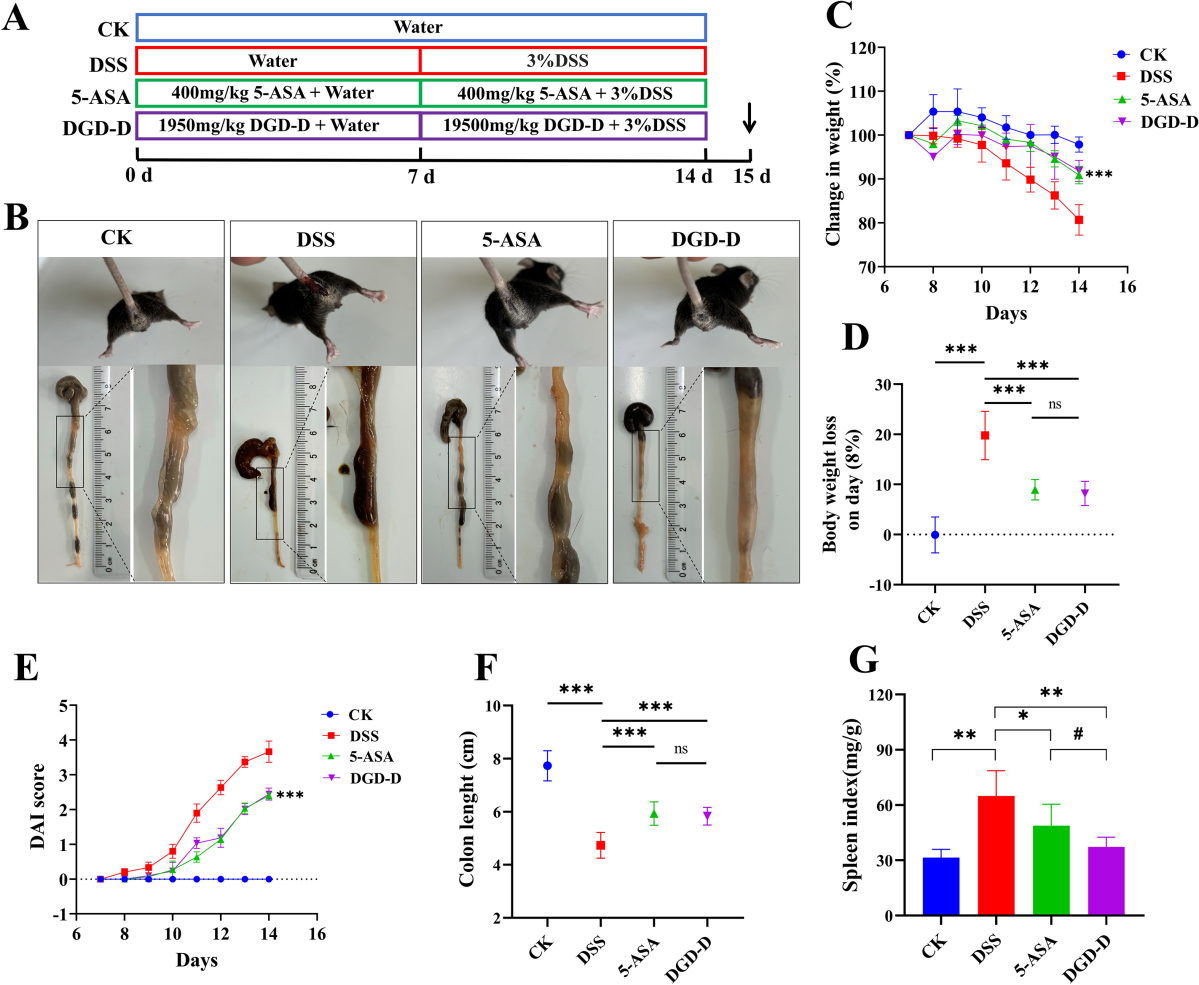
DGD-D alleviates DSS-induced acute colitis in mice. **(A)** showing the study design. **(B)** clinical symptoms and colon morphology on day 14 **(C)** body weight changes. **(D)** weight loss on day 14. **(E)** DAI scores. **(F)** colon length. **(G)** spleen index. Each group included six mice. *P < 0.05, **P < 0.01, ***P < 0.001, relative to the DSS model control group, #*P* < 0.05 relative to the 5-ASA positive control group.

In Part II, as depicted in the diagram of experimental design (**Figure 1A**), 24 mice (male) were divided into four groups at random: control (CK), DSS model (DSS), DGD-D group (1950 mg/kg, based on TCM literature (15, 16)and pilot studies), and 5-ASA group (400 mg/kg; Shanghai Aidefa Pharmaceutical Co., Ltd, Shanghai, China) via gavage. During the 0–14 days of the experiment, all groups received daily gavage as follows: CK and DSS groups, saline; DGD-D group, extrac; 5-ASA group, mesalazine. In order to replicate chronic UC progression in the DSS model, starting from day 8, the CK group was maintained on double-distilled water, whereas the remaining groups were administered a 3% DSS solution (molecular weight: 36,000–50,000 Da; Shanghai Macklin Biochemical Technology Co., Ltd.) for 7 day. Mice were euthanized 24 hours after the last administration under anesthesia induced by intraperitoneal injection of 1.25% avertin (20 mL/kg; Nanjing Aibei Biotechnology Co., Ltd). Serum, colon tissues, and spleen samples were then collected for subsequent analysis.

### 2.4 Pharmacological evaluation

The DAI was determined as the average of the weight change score (WCS), fecal blood score (FBS), and fecal consistency score (FCS), calculated using the formula: DAI = (WCS + FBS + FCS)/3 (17). The spleen index was computed as follows: Spleen index = [spleen mass (mg)/body weight (g)] × 10 (17). Following measurement of length, colon tissue samples were divided into portions: one subset was fixed in 4% paraformaldehyde solution for histological analysis, while the remaining portions were snap-frozen in liquid nitrogen and stored at -80°C for molecular and biochemical assessments.

### 2.5 Quantitative RT-PCR

Total RNA from colonic tissue was quantified and assessed by Nanodrop spectrophotometer (Thermo, USA). The complementary DNA was synthesized from total RNA using the 2× RT OR-Easy™ Mix and Real-Time PCR Easy™ kits (Fujij Biotech Co., Ltd.) from all RNA for qRT-PCR. GAPDH was the internal control, while target genes were IL-1β, IL-6, IL-17, IFN-γ, TNF-α, MPO, ZO-1, Occludin, MUC2, TLR4, MyD88, NF-κB p65 (Rela), NLRP3, and 5-LOX. Relative gene expression was measured by 2^−ΔΔCt method and following primers were used (**Table 1**).

**Table 1:** The particular sequences of the targeted genes primers that were employed for this study.

Gene	Sense primer (5′–3′)	Antisense primer (5′–3′)
β-actin	GTGACGTTGACATCCGTAAAGA	GCCGGACTCATCGTACTCC
L-1β	CCTGTGGCCTTGGGCCTCAA	GAGGTGCTGATGTACCAGTTGG
IL-6	CAAGAGACTTCCATCCAGTTGCCT	TTTCTCATTTCCACGATTTCCCAG
IL-17	CGCAATGAAGACCCTGATA	CTTGCTGGATGAGAACAGAA
IFN-γ	TGAGACAATGAACGCTACACA	TAACAGCCAGAAACAGCCA
TNF-α	CATCTTCTCAAAATTCGAGTGACAA	CATCTTCTCAAAATTCGAGTGACAA
MPO	GAGTCCCACTCAGCAAGGTC	TCTGGCGATTCAGTTTGGCT
ZO-1	GCCGCTAAGAGCACAGCAA	GCCCTCCTTTTAACACATCAGA
Occludin	TTGAAAGTCCACCTCCTTACAGA	CCGGATAAAAAGAGTACGCTGG
MUC2	GCCCACCTCACAAGCAGTAT	GTCATAGCCAGGGGCAAACT
TLR4	ATGGCATGGCTTACACCACC	GAGGCCAATTTTGTCTCCACA
MyD88	AACTGGAGACACAAGCGGAC	CATCCGGCGGCACCAAT
NF-κB p65(Rela)	CACACCCCACCATCAAGATCAA	CTCTATAGGAACTATGGATACTGCG
NLRP3	TGTTCAGCTCTGACCTCTGTG	TGAGGCTGCAGTTGTCTAATTCCA
5-LOX	ACAATGACTTCGAACGGGGC	TGGAGCCAGTATTTGCGCTT

### 2.6 ELISA

Colon tissues were first homogenized in PBS (1:9, w/v) supplemented with protease inhibitors (Roche, Switzerland). Then the supernatant was collected from it after centrifugation (12,000 × g, 10 min, 4°C). Cytokine levels were then quantified by commercial kits (Shanghai Kexing Trading Co., Ltd) according to the provided protocols.

### 2.7 Histopathological analysis

Following the histopathological protocol, specimens of colonic tissue underwent fixation in neutral-buffered formalin and subsequent embedding within paraffin wax blocks; thereafter, consecutive sections measuring 3 μm in thickness were microtome-sectioned, affixed to charged glass slides, and subjected to conventional H&E staining to enable detailed microscopic study, with resultant tissue alterations being systematically scored via the semiquantitative rubric specified in **Table 2** (18). Goblet cells and glycoproteins were further visualized using periodic acid–Schiff (PAS) and alcian blue–periodic acid–Schiff (AB–PAS) staining. Quantitative assessments were performed using Image-Pro Plus 6.0 software.

**Table 2:** The Scoring criteria of histological changes in this study (18).

Score	Inflammation degree	Crypt damage	Percentage of ulceration affected area
0	No inflammatory cell infiltration	There was no damage	0%
1	Mild inflammatory cell infiltration in mucosal layer	Partial crypt injury	1%–10%
2	Moderate inflammatory cell infiltration in the mucosa	The crypt gap was large, the goblet cells were decreased.	10%–25%
3	Numerous inflammatory cells infiltration in the mucosa	The crypt structure was destroyed and a large area of crypt was lost.	25%–50%
4	Transmural inflammatory infiltration of the muscularis mucosae	The crypts and goblet cells were basically missing	50%–100%

### 2.8 Immunofluorescence analysis

Colon sections that had been dewaxed were blocked with 3% BSA for 30 minutes after undergoing antigen retrieval in EDTA buffer (pH 8.0; Servicebio, Wuhan, China). Primary antibodies targeting ZO-1 (catalog no. 21773-1-AP), occludin (catalog no. 27260-1-AP), and MUC2 (catalog no. 27675-1-AP) sourced from Proteintech Group (Wuhan, China) were incubated with the samples at 4°C for 12–16 h. Following PBS washes, slices were counterstained with DAPI (Servicebio) for 10 minutes, treated for 1 hour in the dark with a fluorescein-conjugated secondary antibody (Cat. No. SA00001-1), and examined under the microscope.

### 2.9 Immunohistochemical analysis

Paraffin-dewaxed sections of colonic tissue were first treated with 10% normal goat serum (Servicebio) for 10 minutes to facilitate permeabilization at 4°C using primary antibodies to ZO-1 (catalog no. GB15195), occludin (catalog no. GB111401), MUC2 (catalog no. 27675-1-AP), TLR4 (catalog no. GB15186), MyD88 (catalog no. GB111554), NF-κB p65 (RELA) (catalog no. GB11997), NLRP3 (catalog no. GB114320), and 5-LOX (catalog no. GB111330). Subsequent rinsing in phosphate-buffered saline preceded a 2 h incubation at ambient temperature with a fluorophore-conjugated goat anti-rabbit secondary antibody (catalog no. G1213), after which nuclear counterstaining with DAPI was applied for 10 minutes prior to fluorescence microscopy acquisition. Digital image analysis for quantitative assessment was conducted via Image-Pro Plus 6.0 software.

### 2.10 16S rRNA gene sequencing

The CK, DSS, and DGD-D groups’ cecal contents were taken on day 15 and kept at -80°C. Using the Illumina MiSeq platform, Suzhou PANOMIX Biomedical Tech Co., Ltd. (Suzhou, China) carried out 16S rRNA gene sequencing. Microbial α-diversity (Chao1, Pielou_e, Shannon, Simpson) and β-diversity (PCoA) were analyzed in QIIME2. Taxonomic differences at phylum and family levels were assessed, and differential taxa were identified using LEfSe (LDA > 4, P < 0.05).

### 2.11 Metabolomics analysis

Colon tissues underwent non-targeted metabolomics profiling using LC-MS at Suzhou Panomics Biomedical Technology Co., Ltd. (Suzhou, China). Chromatographic separation of metabolites was accomplished employing a UPLC HSS T3 column (2.1 × 100 mm, 1.8 μm) under a binary gradient of 0.1% formic acid in water (solvent A) and acetonitrile (solvent B) (0–3 min, 5% B; 3–10 min, 5-95% B) delivered at a flow rate of 0.4 mL/min. Mass spectrometry analysis involved electrospray ionization (ESI) in both positive and negative polarities for ionization and detection, covering a mass-to-charge (m/z) spectrum from 70 to 1000 Da. To explore data patterns, principal component analysis (PCA) was performed via the ropls package within the R environment. Differentially abundant metabolites were selected based on variable importance in projection (VIP) values exceeding 1, combined with Benjamini-Hochberg adjusted P-values below 0.05, enabling robust identification of key biomarkers. These findings were visually represented through volcano plots to highlight fold changes and significance, Venn diagrams to show overlaps across groups, and pie charts to depict proportional distributions of metabolite classes (19). Additionally, Kyoto Encyclopedia of Genes and Genomes (KEGG) pathway mapping was applied to reveal enriched biological pathways, with statistical significance set at *P* < 0.05 to prioritize relevant metabolic networks (20).

### 2.12 Statistical analysis

The data is expressed as the mean ± standard deviation (SD). Statistical analyses were conducted using GraphPad Prism (version 8.0). Data conforming to a normal distribution were analyzed using one-way ANOVA, followed by Tukey’s multiple comparisons *post hoc* test to identify pairwise differences. P-value adjustment was performed using the Bonferroni correction for multiple tests. Differences were deemed statistically significant when p values were *P* < 0.05, ensuring reliable detection of treatment effects while controlling for type I errors.

## 3 Results

### 3.1 Chemical composition and stability of DGD-D

**Figure 2:**
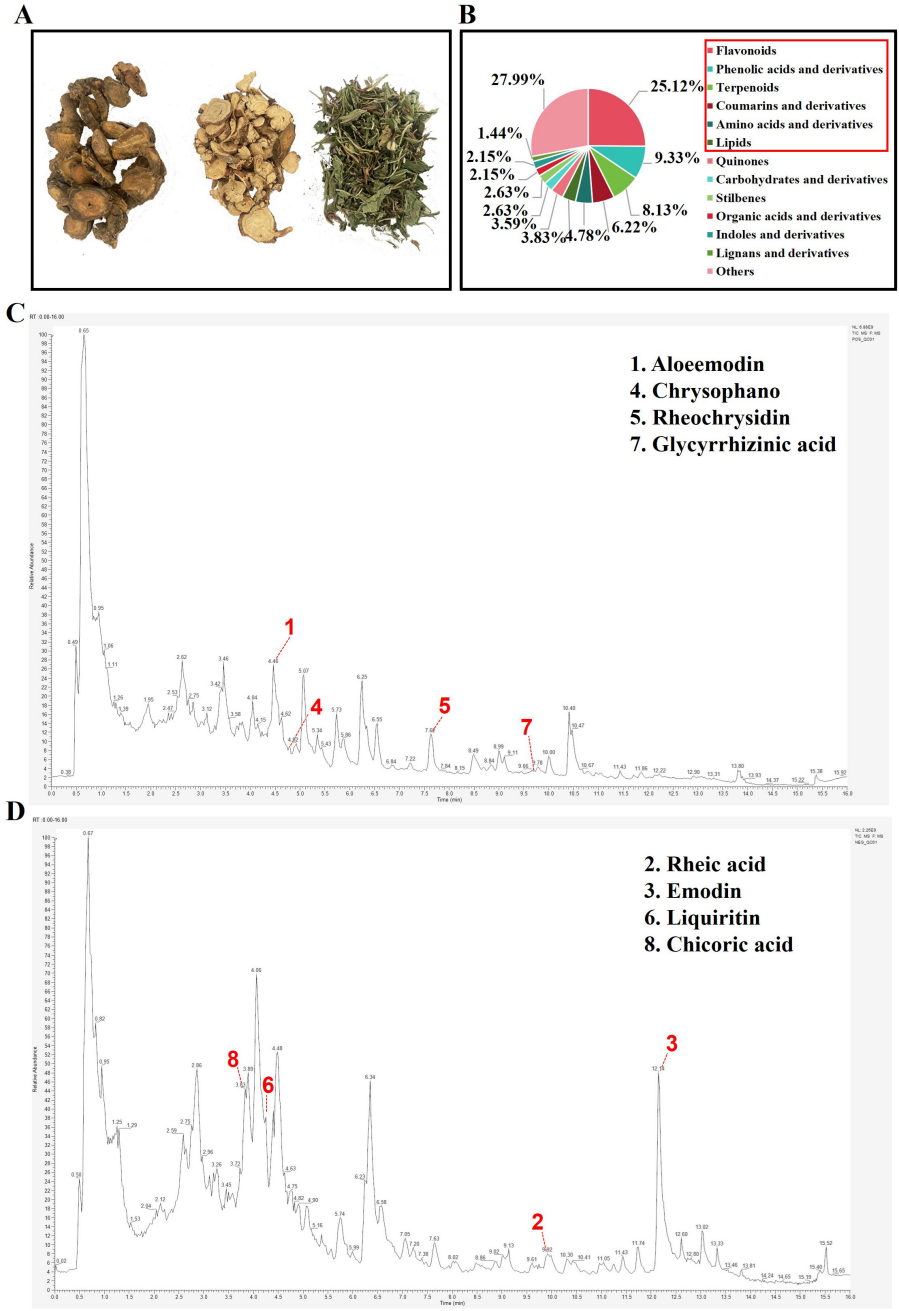
To identify the chemical composition of DGD-D. **(A)** Composition of medicinal ingredients. **(B)** Component type distribution. **(C)** Mode under positive ionization. **(D)** Mode under negative ionization. ① Aloe-emodin, ② Rhein acid, ③ Emodin, ④ Aloeemodin, ⑤ Aloeemodin, ⑥ Liquiritin, ⑦ Glycyrrhizic acid, ⑧ Chicoric acid.

The 418 components were found in Dahuang Gancao Dandelion Decoction (DGD-D), with the following primary classes: flavonoids (25.12%), phenolic acids and derivatives (9.33%), terpenoids (8.13%), coumarins and derivatives (6.22%), amino acids and their derivatives (4.78%) and lipids (3.83%), (**Figure 2B**). Key compounds, consistent with the Pharmacopoeia of the People’s Republic of China (2020), included aloe-emodin, rhein, emodin, chrysophanol, and physcion from rhubarb; glycyrrhizin and glycyrrhizic acid from licorice; and chicoric acid from dandelion (**Figures 2C, D**). These findings confirm the presence and chemical stability of DGD-D’s active components.

### 3.2 Network pharmacology analysis of DGD-D

**Figure 3:**
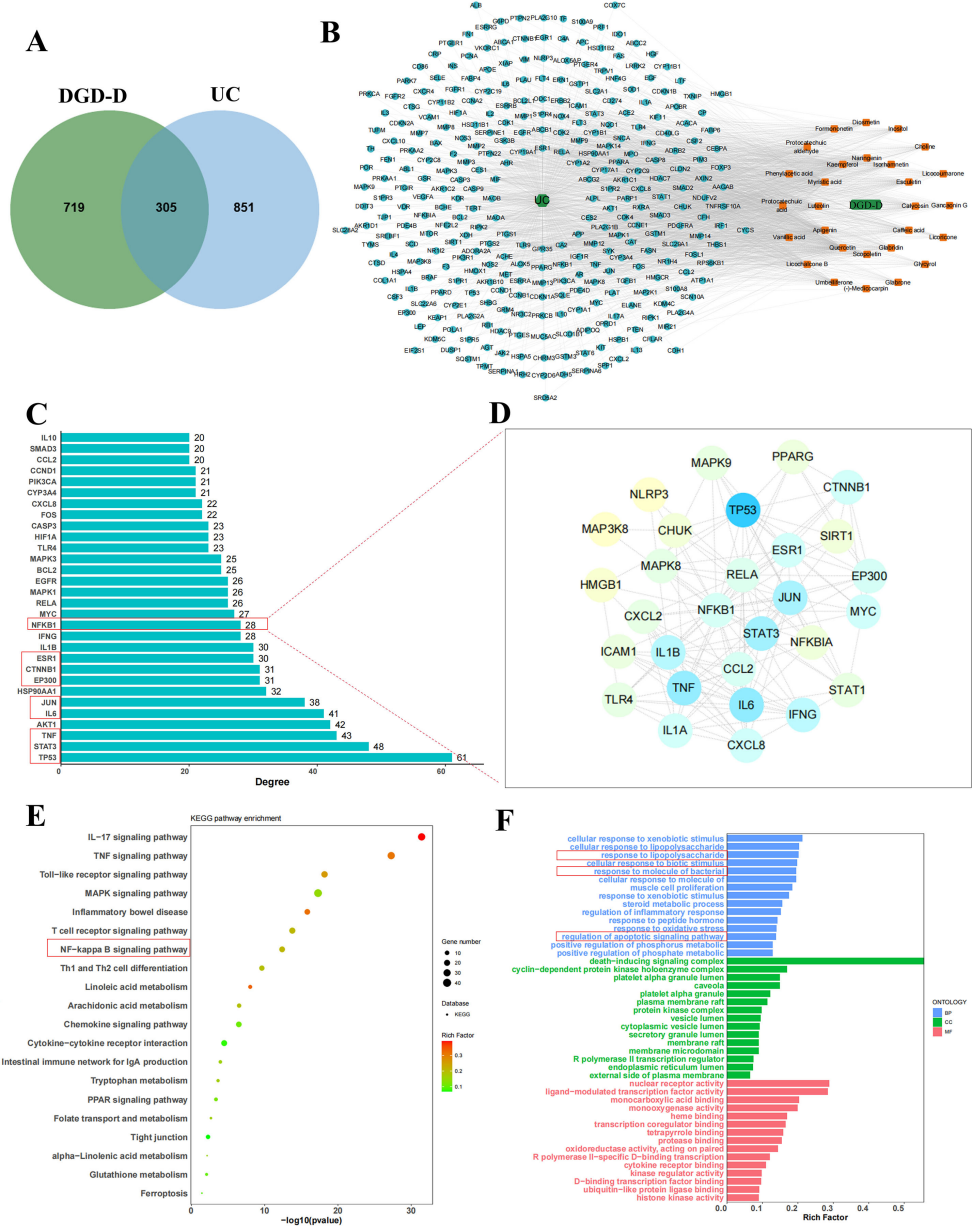
The network pharmacology analysis of the DGD-D in colitis. **(A)** Overlapping targets between DGD-D and ulcerative colitis (UC). **(B)** Network of active compounds and their predicted targets. **(C)** Top 30 key targets identified. **(D)** Protein–protein interaction (PPI) network related to NF-κB signaling. **(E)** KEGG pathway enrichment of shared targets. **(F)** Gene Ontology (GO) enrichment of shared targets.

Based on the detection and identification results of UHPLC-MS, combined with the screening of TCMSP and HERB databases (OB ≥ 30%, DL ≥ 0.18), 28 major active ingredients in DGD-D were identified, targeting 1,024 potential sites in DGD-D, while 1,156 targets associated with UC were recovered. A Venn analysis showed that 305 targets overlapped (**Figure 3A**). According to a compound-target-disease network, the top targets were TP53, STAT3, TNF, AKT1, JUN, IL-6, EP300, HSP90AA1, CTNNB1, and ESR1 (**Figures 3B, C**). Analysis of protein-protein interactions (PPI) revealed that eight of these targets were linked to NF-κB (**Figure 3D**). Among the 20 KEGG pathways (*P* < 0.05), nine were inflammation-related, with NF-κB signaling as the primary focus (**Figure 3E**). GO analysis revealed enrichment in the control of apoptosis, inflammatory responses, and lipopolysaccharide-mediated signaling (**Figure 3F**).

### 3.3 Safety and efficacy of DGD-D

CK group showed no pathological alterations in the liver, spleen, kidneys, and small intestine, with normal tissue structure and healthy status. Compared to the CK group, the Low, Medium, and high groups exhibited no significant differences in any of the aforementioned tissues. (**Supplementary Figure S1**).

DSS induced diarrhea, rectal bleeding, weight loss, colon shortening, and spleen enlargement in mice, confirming successful UC modeling (**Figure 1B**). Compared to the DSS group, DGD-D treatment significantly reduced diarrhea and rectal bleeding, attenuated weight loss (*P* < 0.01 or *P* < 0.001), lowered Disease Activity Index (DAI) scores (*P* < 0.001), restored colon length (*P* < 0.001), and decreased spleen index (*P* < 0.01) (**Figures 1C, G**). These effects were comparable to mesalazine (5-ASA; *P* > 0.05).

### 3.4 Regulation of colonic cytokines by DGD-D

**Figure 4:**
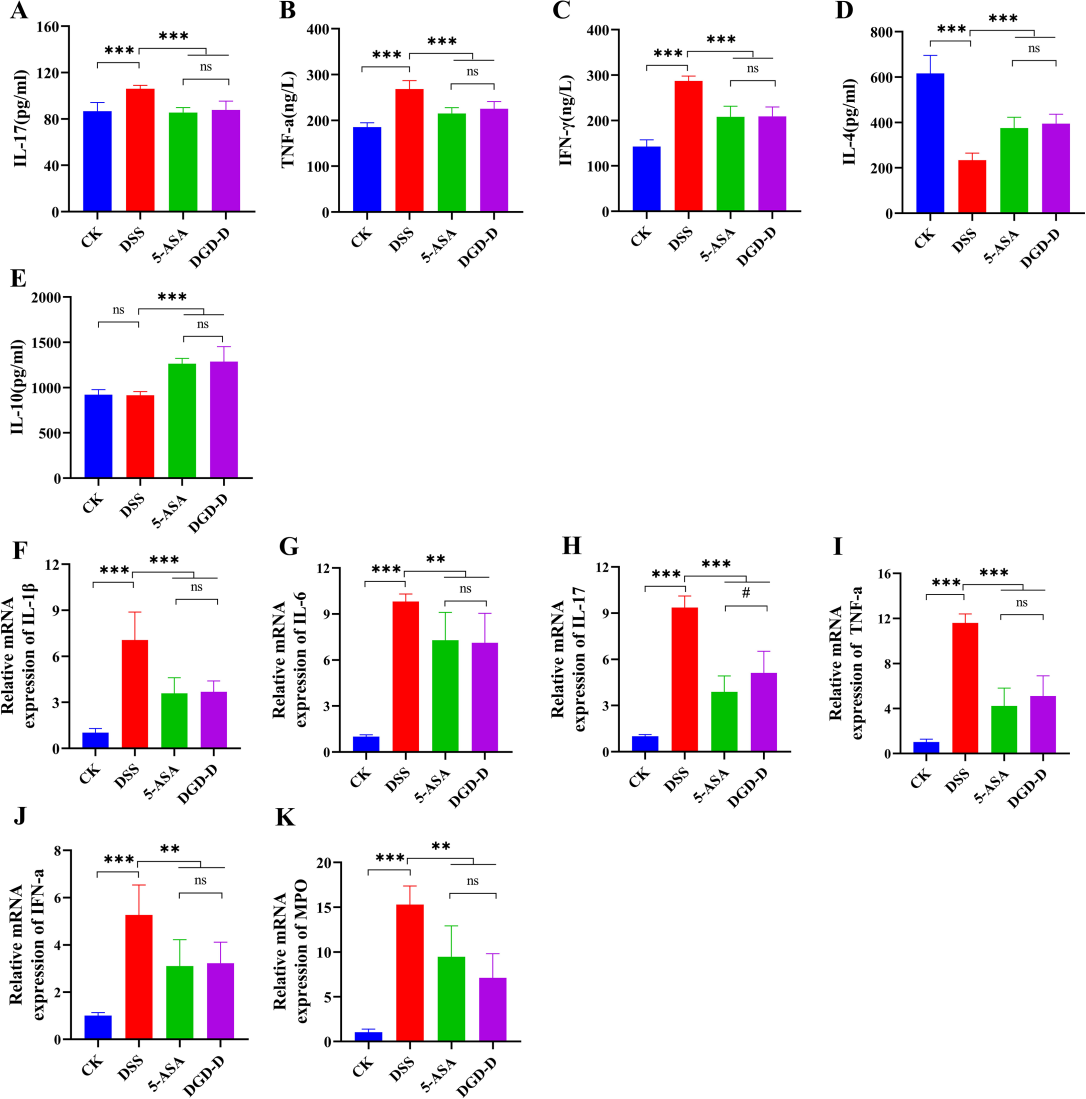
DGD-D modulates cytokine expression in DSS-induced colitis. **(A–E)** Protein levels of IL-4, IL-17, IFN-γ, IL-10, and IL-1β in colon tissues. **(F–K)** Relative mRNA expression of IL-1β, IL-6, IL-17, TNF-α, IFN-γ, and MPO. Each group included six mice. **P < 0.01, ***P < 0.001 relative to the DSS model control group, #*P* < 0.05 relative to the 5-ASA positive control group.

ELISA showed DSS significantly elevated IL-17, TNF-α, and IFN-γ levels (*P* < 0.001) and reduced IL-4 (*P* < 0.001), with no change in IL-10 (**Figures 4A–E**). DGD-D and 5-ASA treatments decreased IL-1β, IL-17, TNF-α, and IFN-γ while increasing IL-4 and IL-10 (*P* < 0.001). qRT-PCR confirmed DSS-induced upregulation of IL-1β, IL-6, IL-17, TNF-α, IFN-γ, and myeloperoxidase (MPO) mRNA (*P* < 0.01 or *P* < 0.001), which DGD-D and 5-ASA significantly downregulated (*P* < 0.01 or *P* < 0.001) (**Figures 4F–K**).

### 3.5 DGD-D improves colonic histological structure

**Figure 5:**
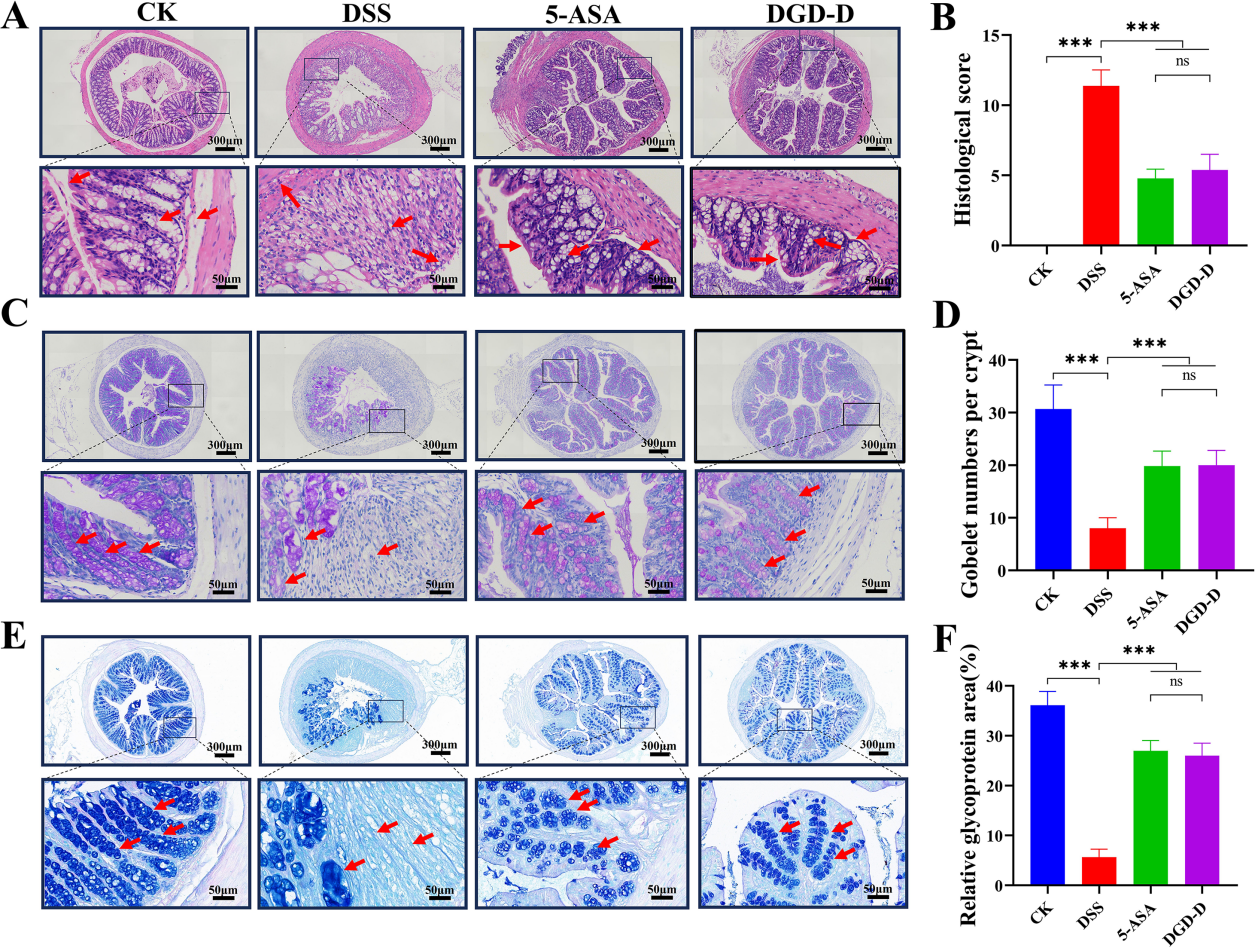
DGD-D restores colonic architecture and mucosal integrity. **(A)** HE (red arrows) demonstrate the anatomy of the mucosa and submucosa (×50, ×300; scale bars, 300 μm, and 50 μm). **(B)** HE staining-based histological scores. **(C)** Goblet cells (red arrows) are highlighted by PAS staining (×50, ×300; scale bars, 300 μm, and 50 μm). **(D)** Areas with PAS are quantified. **(E)** AB-PAS staining demonstrating the distribution of mucin (red arrows) (×50, ×300; scale bars, 300 μm, and 50 μm). **(F)** Areas with AB-PAS positivity are quantified. Each group included six mice. ***P < 0.001 relative to the DSS model control group.

In mice treated with DSS, hematoxylin-eosin (HE) staining showed increased histological scores (*P* < 0.001) along with significant epithelial damage, crypt loss, and inflammatory cell infiltration. Histological scores were lowered (*P* < 0.001) by DGD-D and 5-ASA treatments, which also decreased crypt damage, submucosal edema, and immune cell infiltration (**Figures 5A, B**). PAS and AB-PAS staining revealed reduced content goblet cell numbers and glycoprotein in the DSS group, which 5-ASA and DGD-D restored (*P* < 0.001) (**Figures 5C–F**).

### 3.6 DGD-D enhances intestinal barrier integrity

**Figure 6:**
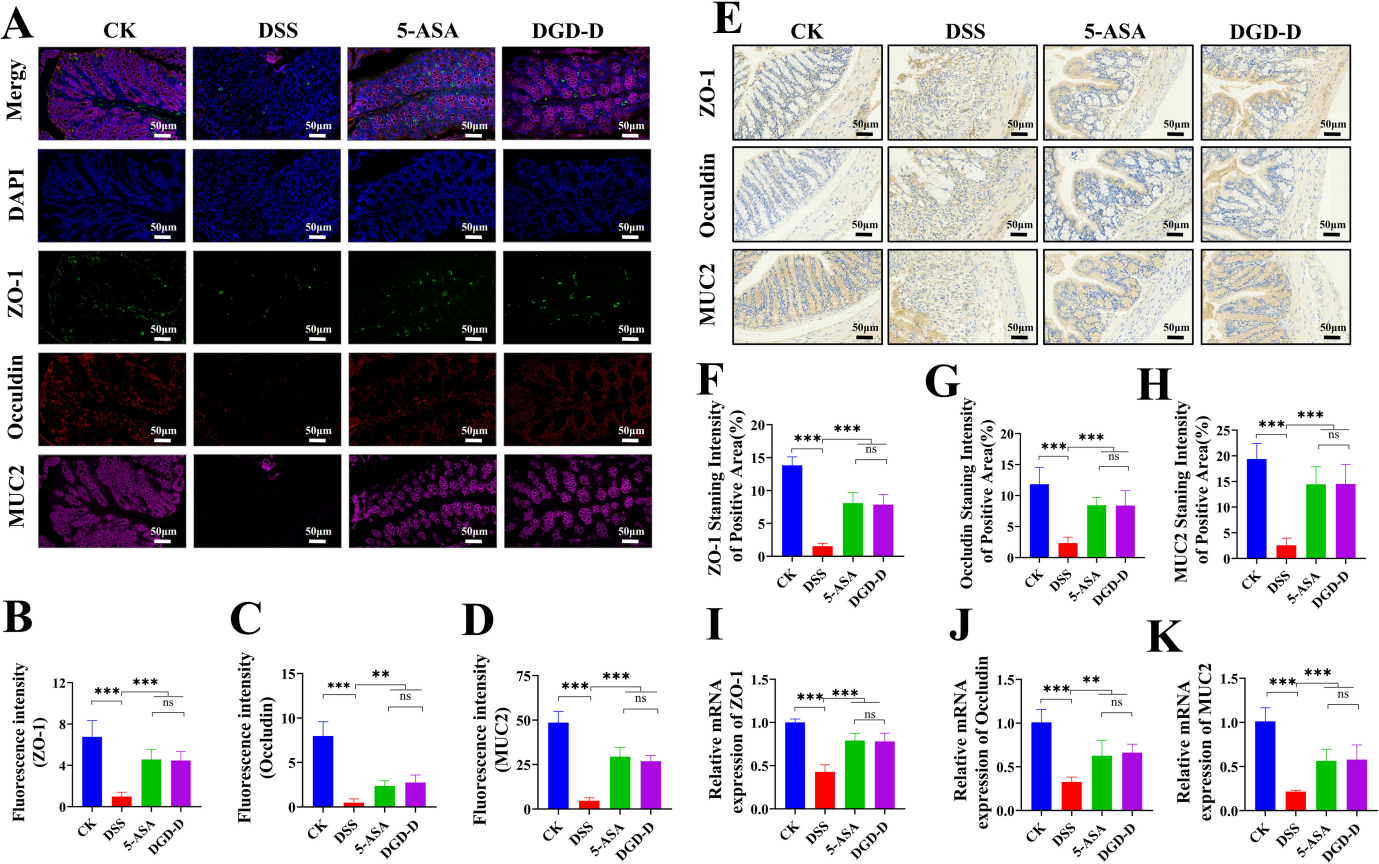
DGD-D enhances intestinal barrier integrity in DSS-induced colitis. **(A)** Representative immunofluorescence pictures (×300; scale bar, 50 μm) demonstrating the expression of MUC2, Occludin, and ZO-1 in colonic tissues. **(B–D)** Measurement of ZO-1, occludin, and MUC2 relative fluorescence intensity, respectively. **(E)** Representative immunohistochemistry staining pictures (×300; scale bar, 50 μm). **(F–H)** Quantification of ZO-1, occludin, and MUC2 IHC-positive staining areas, respectively. **(I–K)** mRNA levels of ZO-1, Occludin, and MUC2 expression in colonic tissues. Each group included six mice. **P < 0.01, ***P < 0.001 relative to the DSS model control group.

The findings of IF, IHC, and qRT-PCR analysis showed DSS significantly reduced ZO-1, Occludin and MUC2 expression levels (*P* < 0.001). DGD-D treatment increased these barrier proteins’ expression (*P* < 0.01 or *P* < 0.001), comparable to 5-ASA (*P* > 0.05) (**Figures 6A, K**).

### 3.7 DGD-D modulates gut microbiota dysbiosis

**Figure 7:**
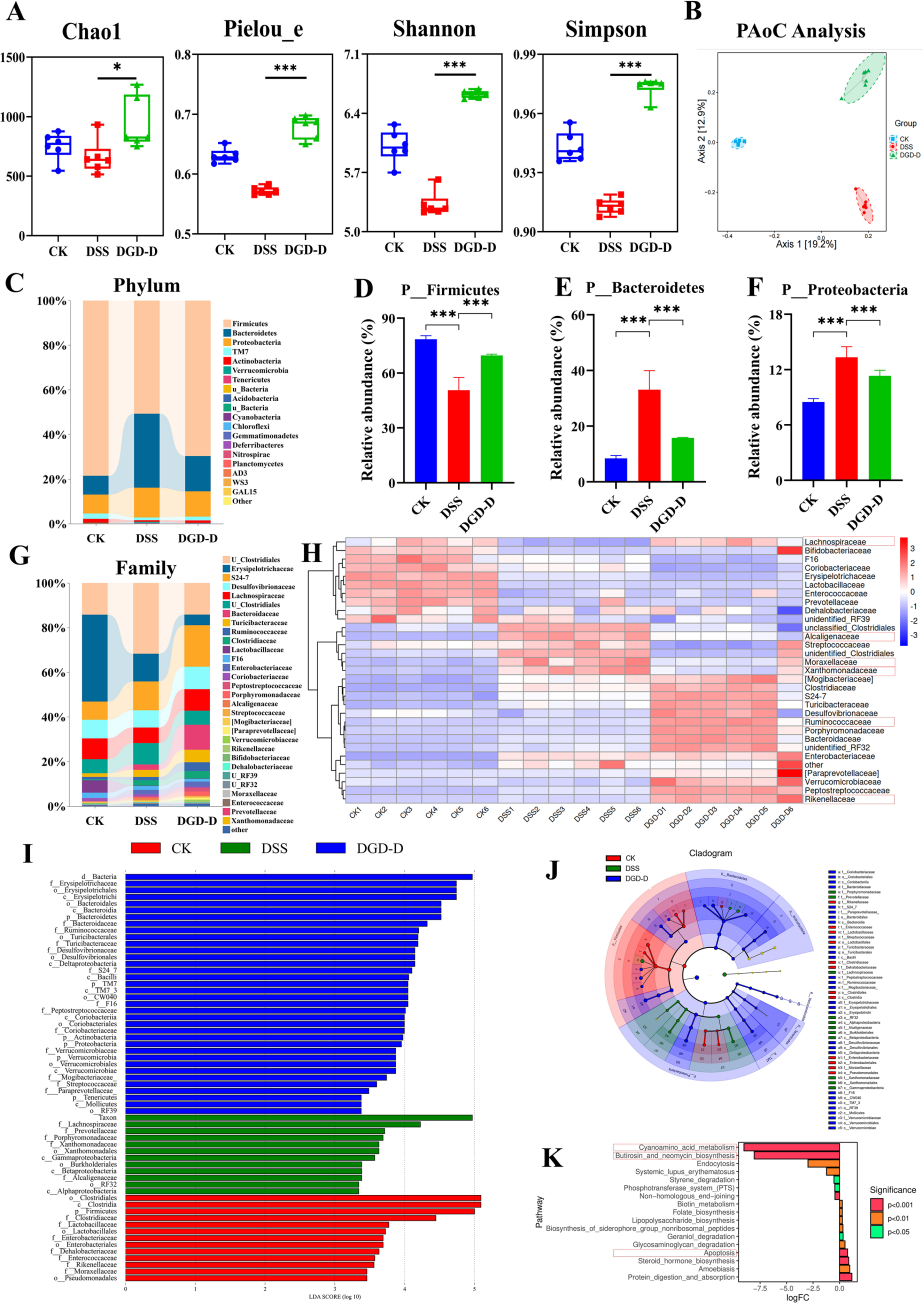
DGD-D restores gut microbiota in the DSS-induced colitis. **(A)** α-Diversity indices. **(B)** Principal coordinates analysis (PCoA) of OTU profiles illustrating group separation. **(C)** Relative abundance of gut microbiota at the phylum level. **(D–F)** Relative changes in *Firmicutes***(D)**, *Bacteroidetes***(E)**, and *Proteobacteria***(F)** respectively. **(G)** The relative level of gut microbiota. **(H)** Heatmap illustrating species with significant differences among groups. **(I)** Cladogram of microbiota enriched among groups as identified by LEfSe analysis. **(J)** LEfSe was employed to pinpoint microbial taxa exhibiting significant enrichment differences across experimental groups. **(K)** KEGG predicted pathways for between group differences. Each group included six mice. Statistical significance levels are indicated as follows: *P < 0.05, ***P < 0.001 relative to the disease model control group.

DSS decreased microbial α-diversity, according to 16S rRNA sequencing (Chao1, Pielou_e, Shannon, Simpson indices). These parameters were improved by DGD-D (*P* < 0.05 or *P* < 0.001) (**Figure 7A**). Different microbial profiles were shown by Principal Coordinate Analysis (PCoA) for the CK, DSS, and DGD-D groups (**Figure 7B**). DSS reduced the *Firmicutes/Bacteroidetes* ratio at the phylum level by increasing *Bacteroidetes* and *Proteobacteria* and decreasing *Firmicutes*. These patterns were reversed by DGD-D (*P* < 0.001) (**Figures 7C–F**). At the family level, DSS increased *Alcaligenaceae*, *Moraxellaceae*, and *Xanthomonadaceae*, while decreasing *Lachnospiraceae*; DGD-D restored these abundances and increased *Ruminococcaceae* (*P* < 0.05) (**Figures 7G, H**),. LEfSe confirmed enrichment of *Ruminococcaceae* in theDGD-D groups, while *Alcaligenaceae Moraxellaceae*, and *Xanthomonadaceae*, was significantly enriched in the DSS group (**Figures 7I, G**). The results of KEGG pathway prediction showed that there were significant differences in the enrichment levels of pathways such as Cyanoamino acid metabolism, Butirosin and neomycin biosynthesis, and Apoptosis between the DSS Vs DGD-D group (*P* < 0.001) (**Figure 7K**).

### 3.8 DGD-D regulates colonic metabolic pathways

**Figure 8:**
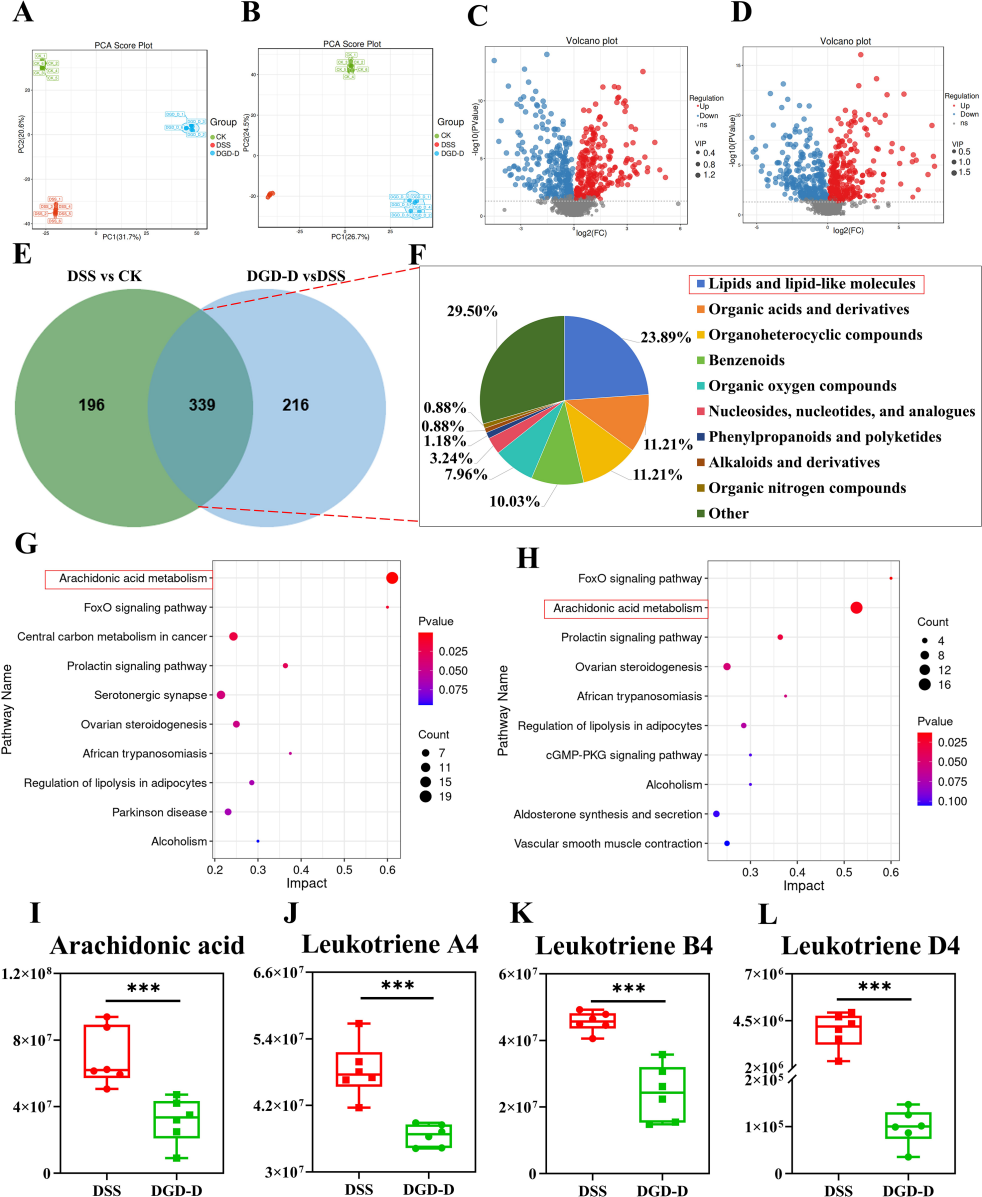
DGD-D modulates colonic metabolite profiles in DSS-induced ulcerative colitis. **(A, B)** Principal component analysis (PCA) illustrating distinct clustering of metabolite profiles among groups. **(C, D)** Volcano plots showing differential metabolites between DSS vs CK **(C)** and DGD-D vs DSS **(D)**. **(E)** Venn diagram depicting shared and unique differential metabolites across groups. **(F)** Proportional distribution of common metabolites among the three groups. **(G, H)** Enrichment analysis for differential metabolites in DSS vs CK **(G)** and DGD-D vs DSS **(H)**. **(I–L)** Relative levels of key arachidonic acid pathway metabolites, including Arachidonic acid **(I)**, Leukotriene A4 **(J)**, Leukotriene B4 **(K)**, and Leukotriene D4 **(L)**. Each group included six mice. Statistical significance levels are indicated as follows: ***P < 0.001, relative to the disease model control group.

Non-targeted metabolomics via LC-MS showed distinct metabolic profiles among CK, DSS, and DGD-D groups (**Figures 8A, B**). Of 1,232 identified metabolites, 535 (258 upregulated, 277 downregulated) differed between DSS and CK (VIP > 1, *P* < 0.05), and 555 (264 upregulated, 291 downregulated) differed between DGD-D and DSS (VIP > 1, *P* < 0.05) (**Figures 8C, D**). Venn analysis identified 339 shared differential metabolites, with lipids and lipid-like molecules comprising 23.89% (**Figures 8E, F**). KEGG enrichment revealed ARA, prolactin, and FoxO signaling pathways as significantly affected (*P* < 0.05) in both the DSS vs CK and DGDG-D vs DSS comparisons. Additionally, in the ARA metabolic pathway, DGD-D downregulated ARA, leukotriene A4 (LTA4), leukotriene B4 (LTB4), and leukotriene D4 (LTD4) levels (*P* < 0.001) (**Figures 8G–J**). As shown in **Supplementary Figure S2**, *Lachnospiraceae* and *Ruminococcaceae* were significantly negatively correlated with ARA, LTA4, LTB4, and LTD4 (*P* < 0.001). In contrast, *Alcaligenaceae* and *Moraxellaceae* showed significantly positive correlations with ARA, LTA4, LTB4, and LTD4 (P < 0.001). Additionally, *Xanthomonadaceae* was also significantly positively correlated with LTA4 and LTB4 (*P* < 0.001) (**Supplementary Figure S2**).

### 3.9 DGD-D inhibits NF-κB/ARA signaling pathway

**Figure 9:**
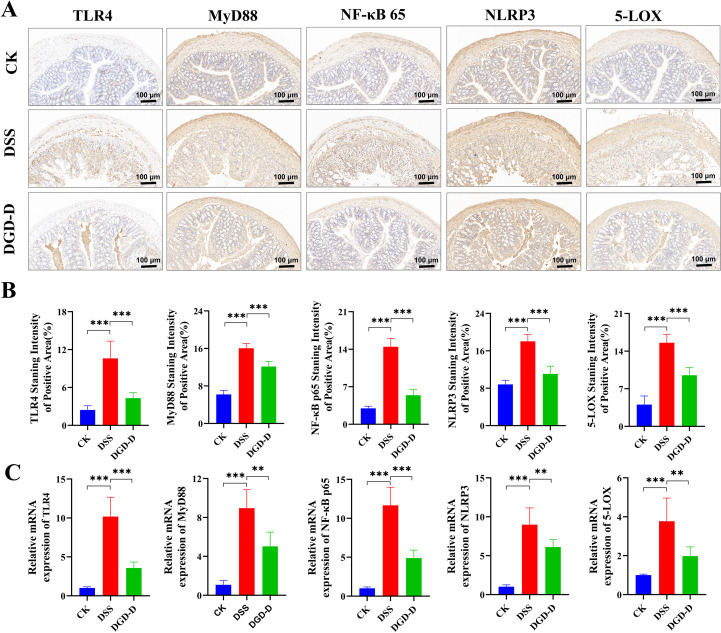
DGD-D suppresses NF-κB/ARA pathway activation to alleviate colonic inflammation. **(A)** Immunohistochemical images (×100; scale bar = 100 μm). **(B)** Quantification of immunofluorescence-positive areas for TLR4, MyD88, NF-κB p65, NLRP3, and 5-LOX. **(C)** Relative mRNA expression levels of TLR4, MyD88, NF-κB p65, NLRP3, and 5-LOX in colon tissues. Each group included six mice. Statistical significance levels are indicated as follows: **P < 0.01, ***P < 0.001, relative to the disease model control group.

IHC and qRT-PCR analyses showed DSS upregulated TLR4, MyD88, NF-κB p65, NLRP3, and 5-LOX expression in colon tissues (*P* < 0.001). DGD-D treatment downregulated these markers (*P* < 0.01 or *P* < 0.001) (**Figures 9A–E**). These findings indicate DGD-D suppresses NF-κB/ARA signaling activation.

## 4 Discussion

Traditional Chinese medicine (TCM) compounds demonstrate significant advantages in the prevention and treatment of UC due to their favorable safety profile and multiple effects, such as modulating gut microbiota and restoring intestinal barrier function (21). To further investigate the relationship between DGD-D and UC, the UPLC-MS/MS method and network pharmacology were used. PPI network construction and KEGG pathway enrichment analysis revealed that the NF-κB signaling pathway likely plays a pivotal role in mediating the anti−colitis effect of DGD-D (**Figures 3D, E**). The NF-κB pathway is involved in inflammatory responses, apoptosis, and cellular differentiation, and can be activated by upstream signals such as TLR4/MyD88 (22). In the context of ulcerative colitis, activation of this pathway not only promotes the expression of pro−inflammatory cytokines, including IL−1β, IL−6 and TNF−α, but also modulates inflammatory−related signaling such as ARA, thereby aggravating pyroptosis and tissue damage (23).

Human UC symptoms, such as diarrhea, colon shortening, and weight loss, are replicated in DSS-induced UC mice model (24). With outcomes similar to those of mesalazine (5-ASA), DGD-D administration significantly restored colon length, attenuated splenomegaly, and decreased DAI scores (**Figure 1**). Anti-inflammatory cytokines (IL-4, IL-10) and Pro-inflammatory cytokines (IL-1β, IL-6, IL-17, TNF-α, and IFN-γ) are out of balance in UC, and tissue damage is exacerbated by increased MPO (25, 26). DGD-D restored immunological balance by upregulating IL-4 and IL-10 and suppressing pro-inflammatory cytokines and MPO (**Figure 4**). The above results indicate that DGD-D can effectively alleviate the pathological symptoms of colitis, restored immunological balance, and thereby exert a protective effect again UC.In the progression of UC, the intestinal epithelium is gradually damaged, with a reduction in the number of goblet cells, Intestinal endotoxins and pathogenic molecules to cross the mucosal layer, and cause damage to tight junction proteins (e.g. ZO-1 and Occludin), and mucins (MUC2), thus resulting in impaired intestinal structure and barrier function (27, 28). DSS-induced barrier disruption was mitigated by DGD-D, which restored goblet cell numbers, reduced inflammatory cell infiltration, and enhanced ZO-1, Occludin, and MUC2 expression (**Figures 5**, **6**), suggesting robust epithelial protection.Gut microbiota dysbiosis is central to UC pathogenesis, with a reduced *Firmicutes/Bacteroidetes* ratio and increased *Proteobacteria* promoting inflammation (29). DGD-D restored microbial balance by increasing *Firmicutes/Bacteroidetes* ratio and reducing *Proteobacteria* abundance (**Figures 7C–F**). At the family level, DGD-D enhanced beneficial taxa within the phylum *Firmicute*, including *Lachnospiraceae* and *Ruminococcaceae*, while decreasing pathogenic taxa in the *Proteobacteria*, such as *Alcaligenaceae*, *Moraxellaceae*, and *Xanthomonadaceae* (**Figures 7G–K**). *Firmicutes* can produce butyrate, which inhibits the NF-κB signaling pathway, thereby suppressing the degradation of tight junction proteins (e.g., ZO-1 and Occludin) and restoring intestinal barrier integrity (30). In contrast, the abnormal increase of Proteobacteria in UC elevates luminal LPS levels (31). This LPS binds to Toll−like receptor 4 (TLR4) on epithelial and immune cells, activating multiple inflammatory signaling pathways and triggering a massive release of pro−inflammatory cytokines, which further disrupts intestinal barrier function (32). Based on the above research results, DGD-D might significantly mitigate the development of UC by altering the abundance of *Firmicutes* and *Proteobacteria*, thus influencing the overall structure of gut microbiota.

**Figure 10:**
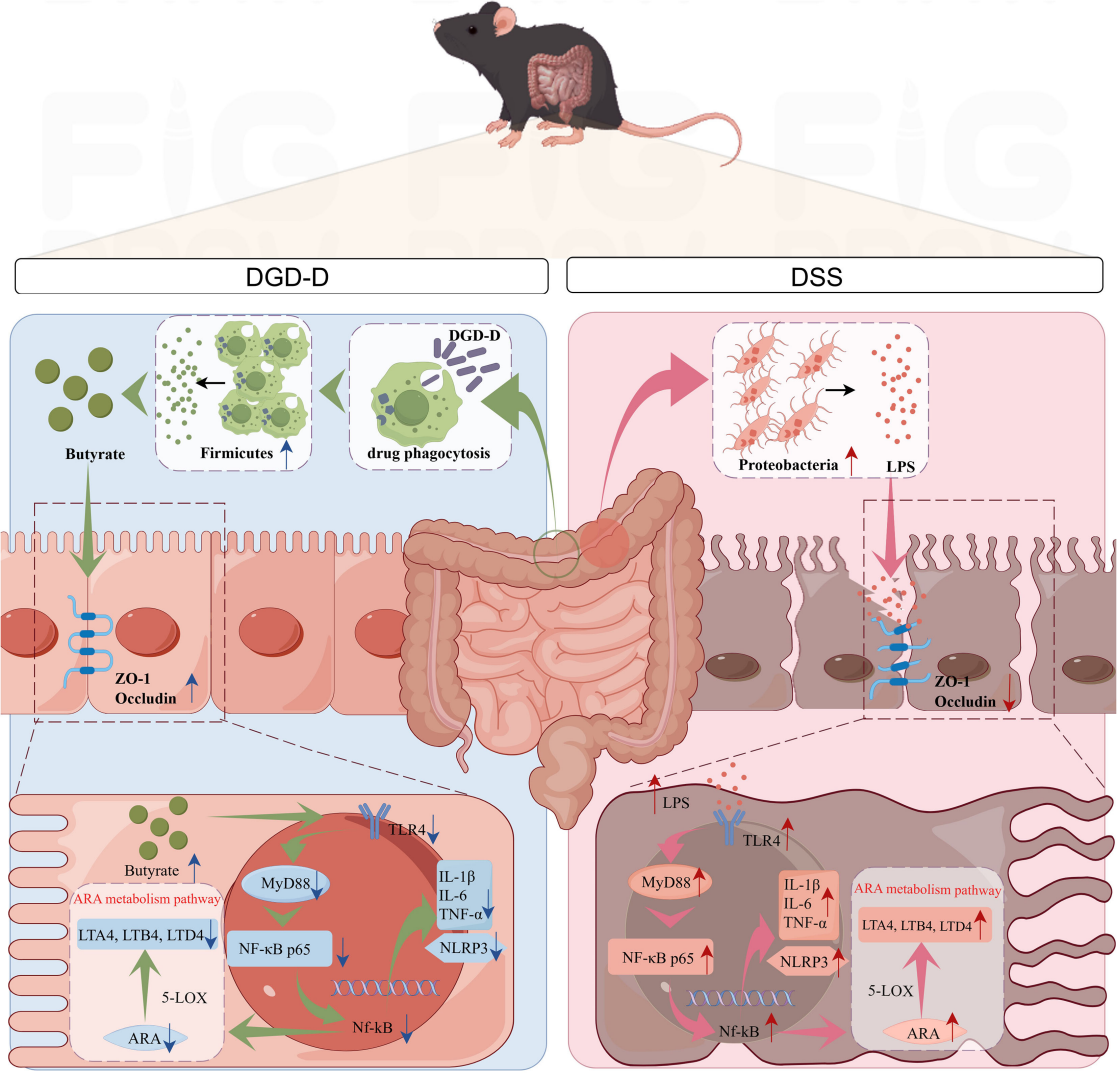
Mechanism of DGD-D repairing intestinal tract in mice with ulcerative colitis.

The development of UC not only disrupts the intestinal microbiota homeostasis but also causes abnormal changes in intestinal metabolites and metabolic pathways (33). To clarify the potential regulatory effect of DGD-D on intestinal metabolic substances and metabolic pathways, a comprehensive targeted metabolomics sequencing was conducted. During the inflammatory response, ARA is catalyzed by the pro-inflammatory enzyme 5-LOX into leukotriene A4 (LTA4), thereby increasing inflammatory mediators such as LTB4 and LTD4, and further promoting the development of inflammation (13, 34, 35). This study found that the expression of ARA, LTA4, LTB4, and LTD4 was significantly increased in the DSS group of mice, suggesting that DSS upregulated the expression of the ARA metabolism pathway through the 5-LOX pathway, increasing the inflammatory response (**Figures 8I–L**). However, after DGD-D treatment, the levels of these metabolites reversed, suggesting that DGD-D exerts its therapeutic effect in UC by suppressing the ARA metabolic pathway (**Figure 10**).

## 5 Conclusion

By improving intestinal barrier integrity, restoring gut microbial and metabolism balance, and blocking the NF-κB/ARA signaling pathway, DGD-D dramatically reduces DSS-induced UC in mice. These results establish DGD-D as a promising TCM-based treatment for ulcerative colitis (UC), providing TCM researchers with insightful information and laying the groundwork for further clinical study to clarify its molecular processes and therapeutic potential.

## Data Availability

The original contributions presented in the study are publicly available. This data can be found here: 10.6084/m9.figshare.30550928.
