# Supplementary material for: Dahuang gancao dandelion decoction regulates intestinal flora and inhibits NF-κB/ARA signaling pathway to alleviate ulcerative colitis
Source: Front Immunol. 2026 Jan 29;16:1735021. doi: 10.3389/fimmu.2025.1735021 (PMC12893983; doi:10.3389/fimmu.2025.1735021)
Supplement: Supplementary file 1 [file DataSheet1.zip › Ethical Certtificates/Application of Welfare and Ethics Approval for Research involving Animals.pdf]

附件 1

实验动物福利伦理审查申请书

Application of Welfare and Ethics Approval for Research involving Animals

申请日期：2022 年 3 月 20 日

课题名称，编号，来源：<br>刺糖多糖 Pickering 乳液佐剂活性及作用机理的研究，2021D01B48，新疆维吾尔自治区自然科学基金-青年科学基金项目			
项目负责人	职称	所在院所	
阿得力江·吾斯曼	副教授	新疆农业大学动物医学学院	
联系电话：17726793817		E-mail：1445955698@qq.com	
申请目的	初次申请（√） 延长（ ） 修改原申请（原批准号：________）（ ）		
动物实验名称（中文和英文）：多糖及纳米粒对动物的免疫增强作用（ Immune enhancement of polysaccharides and nanoparticles on animals ）			
拟实验日期	2022 年 4 月 25 日至 2022 年 12 月 29 日		

序号	实验执行人	职　称	所在院所	联系电话
1	赛福丁·阿不拉	教　授	动物医学学院	18699116432
2	阿得力江·吾斯曼	副教授	动物医学学院	17726793817
3	萨比热·热夏提	研究生	动物医学学院	15022956854
4	程昕珂	研究生	动物医学学院	15199136206

实验动物来源	新疆医科大学实验动物中心
实验动物许可证和质量合格证明编号	SCXK(新)-2018-0002

品种、品系	年龄或体重	等级	性别	数量	其它
雏鸡	0 日龄	普通	雌性	480 只	
小鼠	0 日龄	普通	雌性	100 只	
小鼠	0 日龄	普通	雄性	100 只	

一、概述本实验的目的及对人类、动物或科学的贡献

当前疫苗临床应用的佐剂主要是油佐剂和铝佐剂，油佐剂存在注射部位产生肉芽肿、结痂等损害，同时不能被机体代谢，易产生机体残留，严重影响禽类产品的品质和安全。铝佐剂安

全性好，能够产生强烈的体液免疫反应，但其缺陷是不能引起明显的细胞免疫反应。基于我省在特色畜禽养殖业中存在的有效疫苗佐剂缺乏的实际问题，急需开发佐剂活性高、作用时间长、毒副作用小的新型免疫佐剂，以满足畜禽类疫苗生产及食品安全的需要。植物多糖及纳米粒佐剂的研究不仅对新疆养殖业的发展具有重要意义，而且对保障畜产养殖业的健康发展有实用价值，也为今后维吾尔药的现代研究开发提供理论和实际依据，同时而且对新疆道地药材资源的充分开发利用及提高当地农民收入具有一定的帮助。

二、请以实验动物“3R”原则（实验动物替代、减少和优化）为考虑重点，说明进行动物实验的必要性，包括非动物模型不合适性及选择该动物品种的理由。

1、使用动物的理由（在括号内打“√”）：

（√）（1）一些生物学过程和机理不能在体外研究

（√）（2）已进行体外实验，现须进行体内实验

（ ）（3）体外实验需要动物组织

（ ）（4）其它

请具体说明：

2、请说明使用动物数量的充分理由：

将所制备的多糖及多糖纳米粒作为疫苗的佐剂，与灭活抗原结合后检测多糖纳米佐剂抗原负载率和体外缓释效果。将通过饲喂和注射两种方式免疫动物，观察免疫后动物体内产生的特异性抗体、体液免疫及细胞免疫的效果，免疫细胞能力影响，从而评价多糖及多糖纳米佐剂的免疫增强效果。

三、描述动物实验的设计

1、 简述实验过程（包括实验基本过程；说明动物保定的必要性，动物保定的方法，包括设备和药物；实验过程中动物有无疼痛，如果有强烈疼痛如何减轻：麻醉药名称、剂量、给药途径和维持时间；实验采集的标本用什么方法检测什么指标。）

取实验动物，采用异氟醚进行呼吸麻醉，麻醉后脱颈处死，收集胸腺、法氏囊、脾脏等免疫器官，进行免疫活性的测定。

3、实验之后动物如何处理？（在括号内打“√”）

（ ）实验后动物可以正常存活，回归正常饲喂

（√）实验结束时动物已经无痛死亡，尸体无公害化处理

（ ）动物不能正常存活，进行安乐死（安乐死方法、使用药物及其剂量）：

声明：

我承诺该申请使用表的内容准确无误。

我同意遵守中华人民共和国国家科学技术委员会制定的《实验动物管理条例》、中华人民共和国科学技术部发布的《关于善待实验动物的指导性意见》。

我承诺包括我自己在内的该申请使用表中提及的与实验动物有接触的人员，已经参加了新疆农业大学实验动物中心要求的相关培训，掌握了申请使用表中涉及的动物实验方法，都有能力完成动物实验，并且深知使用这些活体动物及动物组织所存在的风险。

项目负责人签字：[illegible] 2022 年 3 月 20 日

申报单位意见：

同意

主管领导签字（单位章）：[illegible] 2022 年 3 月 20 日

审查主要依据：

1、该项目是否必须用实验动物进行实验，即能否用计算机模拟和细胞培养等非生命方法替代实验动物或用低等实验动物替代高等实验动物进行实验。

2、表中所填实验相关人员资格和实验相关单位是否合适。

3、表中所填实验所用实验动物的品种品系、质量等级、规格是否合适，能否通过改良设计方案或用高质量的实验动物来减少所用实验动物的数量。

4、能否通过改进实验方法、调整实验观测指标、改良处死实验动物的方法，来优化实验方案、善待实验动物。

审查	实验执行人员资格： 符合要求（√） 不符合要求（ ）
	动物实验方案： 适当（√） 不适当（ ）

审批意见：

（√）同意

（ ）稍作修改，同意

（ ）修改后，再次会议讨论

（ ）不同意

主任委员（签名）：[illegible]

新疆农业大学实验动物福利伦理委员会

Animal Welfare and Ethics Committee of Xinjiang Agricultural University

2022 年 3 月 20 日

批准号<br>Animal protocol number	2022 016

说明：

1、请在实验开始前 1-2 个月提交电子版申请材料到新疆农业大学实验动物福利伦理委员会，接受审查；

2、得到审查反馈意见并修改后，递交电子版和纸质版申请书 2 份（项目负责人签字，申请院所主管科研领导签字、盖章，双面打印）。

3、动物实验程序审核批准后将给予批准号。

Annex 1

Application for Ethical Review of Laboratory animal welfare

Application of Welfare and Ethics Approval for Research involving Animals

Date of application: March 20, 2023

**Project name, Number, Source: Study on the activity and mechanism of Acanthopogon Pickering Emulsion Adjuvant, 2021D01B48, Natural Science Foundation of Xinjiang Uygur Autonomous Region - Youth Science Fund Project**

Project Leader	Job title	Institution
Adelijiang Wusiman	Associate Professor	College of Veterinary Medicine, Xinjiang Agricultural University

**Contact number: 17726793817**	**E-mail: 1445955698@qq.com**
**Purpose of application**	**Initial Application (√) Extension () Modification of original application (original Approval number:) ()**

**Name of animal experiment (Chinese and English) :**<br>**Immune enhancement of polysaccharides and nanoparticles on animals**

**Date of the proposed experiment**	**April 25, 2022 to December 29, 2022**

Serial number	Experimenter	Job title	Institution	Contact number
1	Saifuding Abula	Professor	College of Veterinary Medicine, Xinjiang Agricultural University	18699116432
2	Adelijiang Wusiman	Associate Professor	College of Veterinary Medicine, Xinjiang Agricultural University	17726793817
3	Sabire Rexiati	Graduate student	College of Veterinary Medicine, Xinjiang Agricultural University	15022956854
4	Cheng Xinke	Graduate student	College of Veterinary Medicine, Xinjiang Agricultural University	15199136206

**Laboratory animal sources**	**Experimental Animal Center, Xinjiang Medical University**
**Experimental animal license and quality Certificate number**	**SCXK(New)-2018-0002**

Varieties and strains	Age or weight	Grade	Gender	Quantity	Other
Chick	0 days of age	Normal	Female	480	
Mice	0 days of age	Normal	Female	100	
Mice	0 days of age	Normal	Male	100	

I. A summary of the purpose of the experiment and its contribution to humans, animals, or science

At present, oil adjuvant and aluminum adjuvant are the main adjuvants used in vaccine clinical

application. Oil adjuvant has the presence of granuloma, scab and other damage at the injection site, and can not be metabolized by the body, which is easy to produce body residue, seriously affecting the quality and safety of poultry products. Aluminum adjuvant has good safety and can produce strong humoral immune response, but its defect is that it can not cause obvious cellular immune response. Based on the shortage of effective vaccine adjuvants in the characteristic livestock and poultry industry in our province, it is urgent to develop new immune adjuvants with high activity, long action time and small toxic side effects to meet the needs of livestock and poultry vaccine production and food safety. The research on plant polysaccharides and nanoparticle adjuvants is not only of great significance to the development of Xinjiang's livestock industry, but also has practical value to ensure the healthy development of livestock and poultry industry, and also provides theoretical and practical basis for the modern research and development of Uygur medicine in the future, but also has certain help for the full exploitation and utilization of Xinjiang's authentic medicinal materials and the improvement of local farmers' income.

II. Please take the "3R" principle (replacement, reduction and optimization of laboratory animals) as the focus of consideration, and explain the necessity of conducting animal experiments, including the inadequacy of non-animal models and the reasons for choosing the animal variety.

1. Reasons for using animals (√ in parentheses) :

(√) (1) Some biological processes and mechanisms cannot be studied in vitro

(√) (2) Having performed in vitro experiments, in vivo experiments are now required

() (3) Animal tissues are required for in vitro experiments

() (4) Others

Please specify:

2、Please state a good reason for the number of animals used:

The polysaccharide and polysaccharide nanoparticles prepared were used as vaccine adjuvants. After binding with inactivated antigen, the antigen loading rate and in vitro sustained release effect of polysaccharide nano-adjuvant were detected. Animals were immunized by feeding and injecting to observe the effects of specific antibodies, humoral immunity and cellular immunity, and the effect of immune cell capacity, so as to evaluate the immunological enhancement effect of polysaccharide and polysaccharide nanoadjuvant.

III. Describe the design of animal experiments

1. Briefly describe the experimental process (including the basic process of the experiment; Explain the necessity of animal preservation, the method of animal preservation, including equipment and drugs; Whether the animal is in pain during the experiment, and how to relieve it if there is intense pain: the name of the anesthetic, dose, route of administration and maintenance time; And what measures were used to measure the

specimens collected during the experiment.)

The experimental animals were taken for respiratory anesthesia with isoflurane, and were killed by neck removal after anesthesia. The immune organs such as thymus, bursa of Farcilla and spleen were collected for the determination of immune activity.

3. How to deal with the animals after the experiment? (Put a "√" in brackets)

() Animals can survive normally after the experiment and return to normal feeding

(√) The animal died painlessly at the end of the experiment, and the carcass was disposed of pollution-free

() The animal cannot survive normally and is euthanized (method of euthanasia, drugs used and dosages) :

Declaration:

I promise that the contents of this application form are accurate.

I agree to abide by the Regulations on the Administration of Laboratory Animals formulated by the State Science and Technology Commission of the People's Republic of China and the Guiding Opinions on the Good Treatment of Laboratory Animals issued by the Ministry of Science and Technology of the People's Republic of China.

I promise that all the personnel who have contact with experimental animals mentioned in the application form, including myself, have participated in the relevant training required by the Experimental Animal Center of Xinjiang Agricultural University, have mastered the animal testing methods mentioned in the application form, are capable of completing animal experiments, and are well aware of the risks associated with using these live animals and animal tissues.

Signature of the person in charge of the project: December 2022

Comments of the applicant:

Signature of the competent leader (unit seal) : December 2022

Main basis of review:

1. Whether the project must use experimental animals for experiments, that is, whether non-living methods such as computer simulation and cell culture can be used to replace experimental animals or lower experimental animals can be used to replace higher experimental animals for experiments.
2. Whether the qualifications of experiment-related personnel and experiment-related units are appropriate.
3. Whether the varieties, quality grades and specifications of experimental animals used in the table are appropriate, and whether the number of experimental animals used can be reduced by improving the design scheme or using high-quality experimental animals.
4, whether by improving the experimental method, adjusting the experimental observation indicators, improving the method of killing experimental animals, to optimize the experimental program, treat experimental animals well.

**Review**	**Experimental Executive Qualifications: Meet requirements (√) Do not meet requirements ()**

	**Animal testing protocol: appropriate (√) Inappropriate ()**

**Approval comments:**<br>**(√) Agree**<br>**() Slightly amended, agree**<br>**() After modification, meet again to discuss**<br>**() Disagree**<br>**(Signed) by the Chairman:**<br>**Experimental Animal Welfare Ethics Committee, Xinjiang Agricultural University**<br>**Animal Welfare and Ethics Committee of Xinjiang Agricultural University**<br>**Year Month Day**	
**Approval Number**<br>**Animal protocol number**	**2022016**

Instructions:

1. Please submit the electronic application materials to the Experimental Animal Welfare Ethics Committee of Xinjiang Agricultural University 1-2 months before the start of the experiment for review;
2. After receiving the review feedback and revising, submit two electronic and paper versions of the application (signed by the project leader, signed and stamped by the scientific research leader of the applicant institute, printed on both sides).
3. The approval number will be given after the examination and approval of the animal experiment procedure.
