# Supplementary figures and images for: Dahuang gancao dandelion decoction regulates intestinal flora and inhibits NF-κB/ARA signaling pathway to alleviate ulcerative colitis
Source: Front Immunol. 2026 Jan 29;16:1735021. doi: 10.3389/fimmu.2025.1735021 (PMC12893983; doi:10.3389/fimmu.2025.1735021)

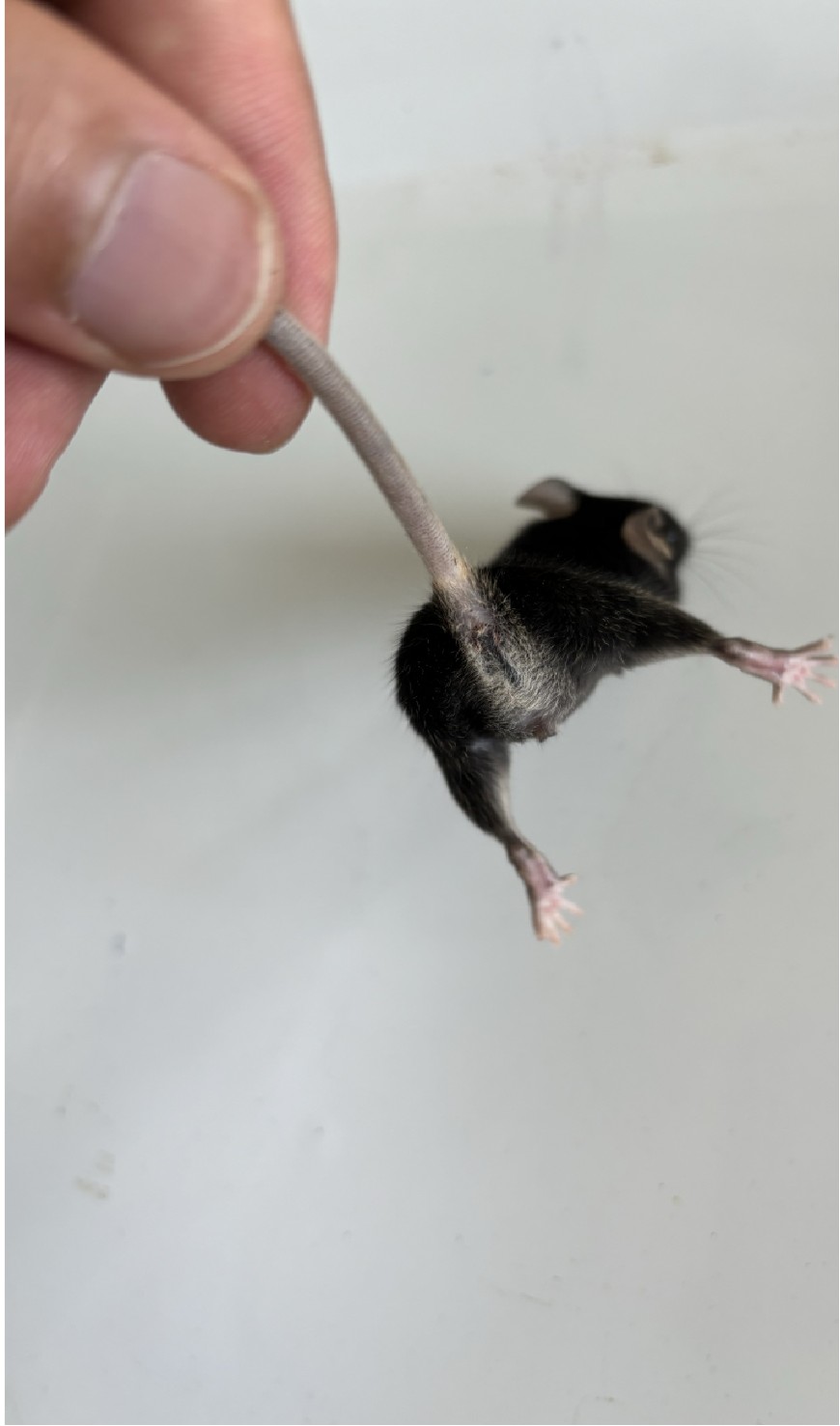

Supplement: Supplementary file 4 [file DataSheet4.zip › Figure-3/fig3-B(Original image of mouse colon)/5-ASA.jpg]

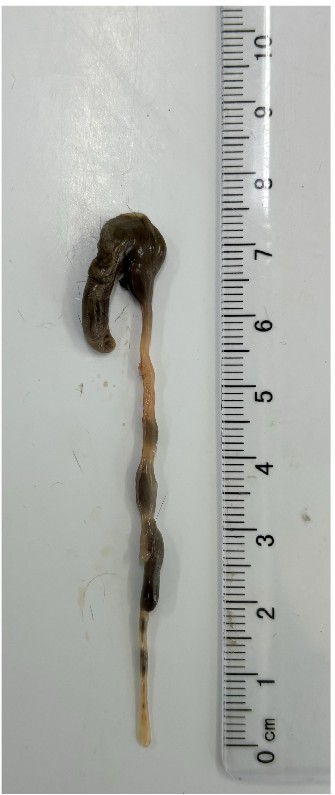

Supplement: Supplementary file 4 [file DataSheet4.zip › Figure-3/fig3-B(Original image of mouse colon)/5-ASA(1).jpg]

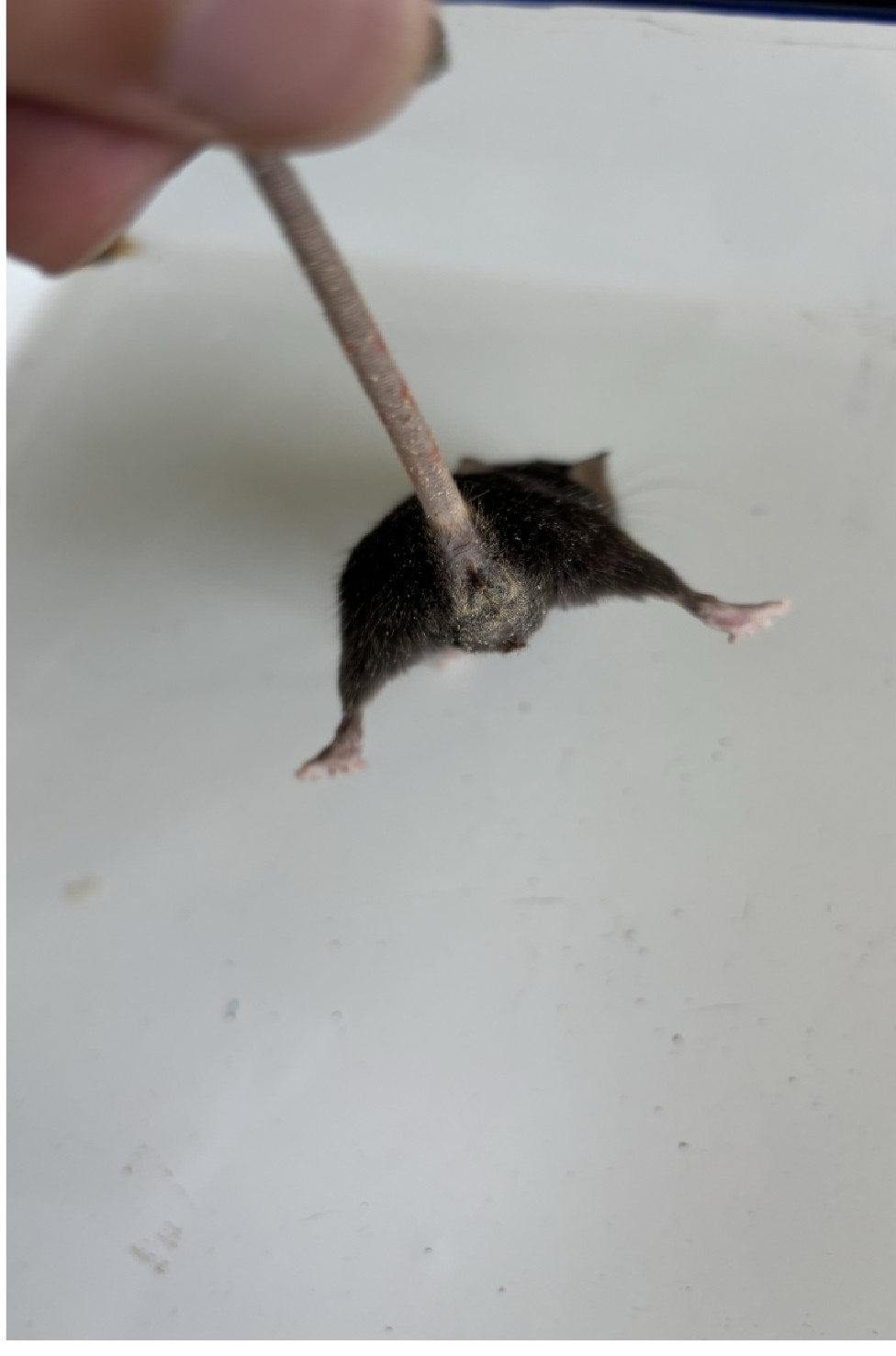

Supplement: Supplementary file 4 [file DataSheet4.zip › Figure-3/fig3-B(Original image of mouse colon)/Control.jpg]

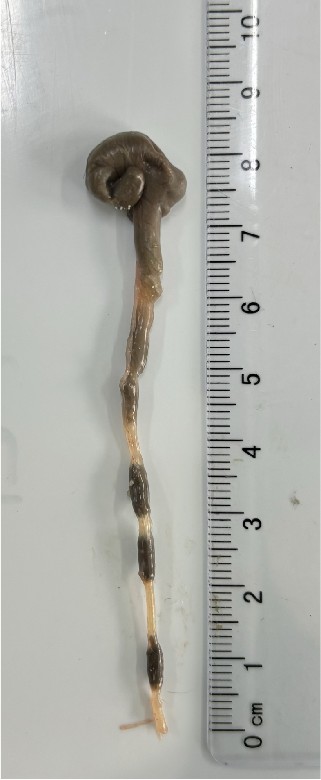

Supplement: Supplementary file 4 [file DataSheet4.zip › Figure-3/fig3-B(Original image of mouse colon)/Control(1).jpg]

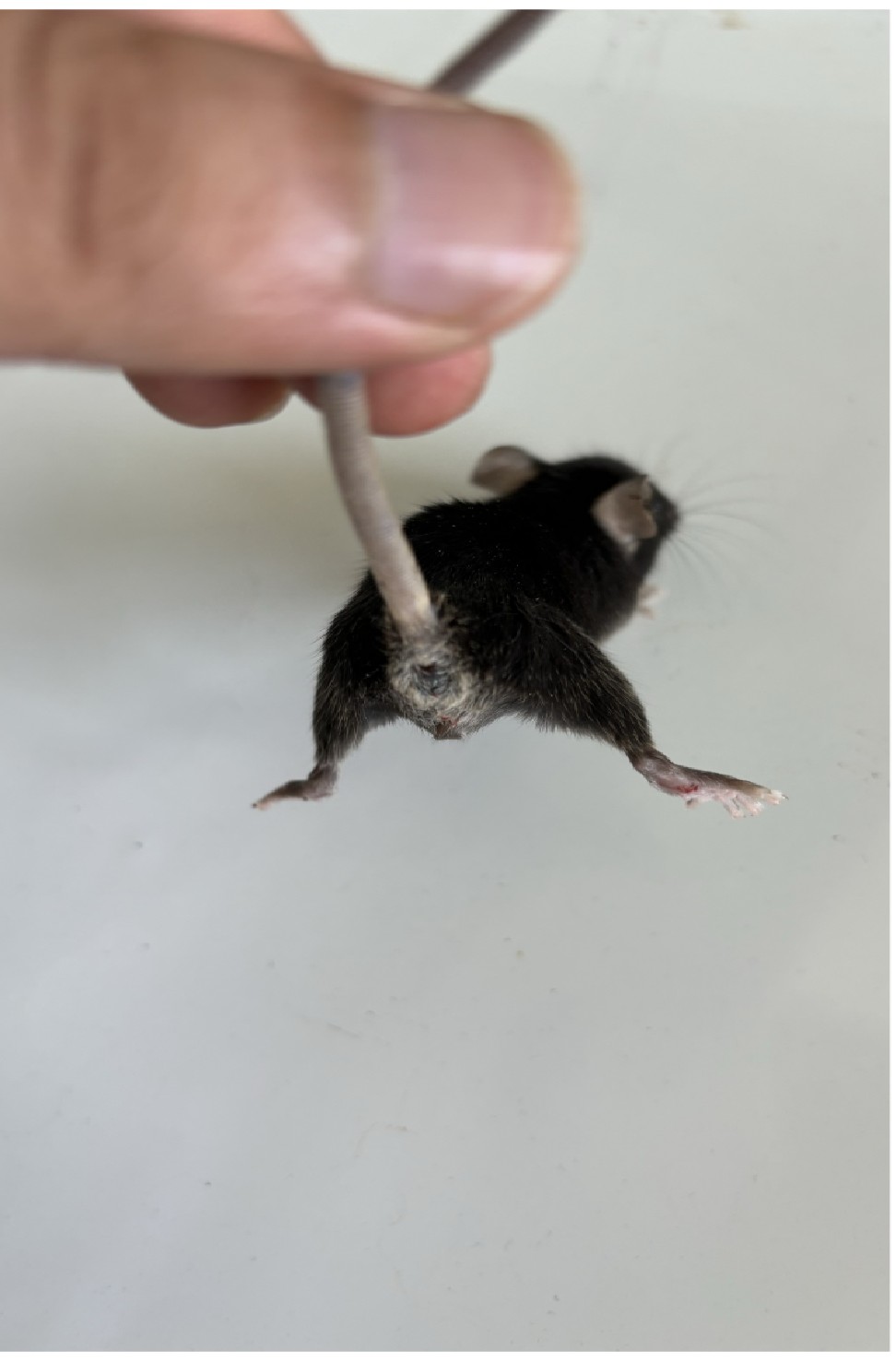

Supplement: Supplementary file 4 [file DataSheet4.zip › Figure-3/fig3-B(Original image of mouse colon)/DGD-D.jpg]

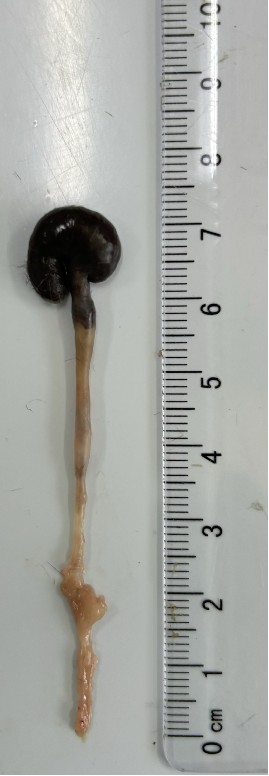

Supplement: Supplementary file 4 [file DataSheet4.zip › Figure-3/fig3-B(Original image of mouse colon)/DGD-D(1).jpg]

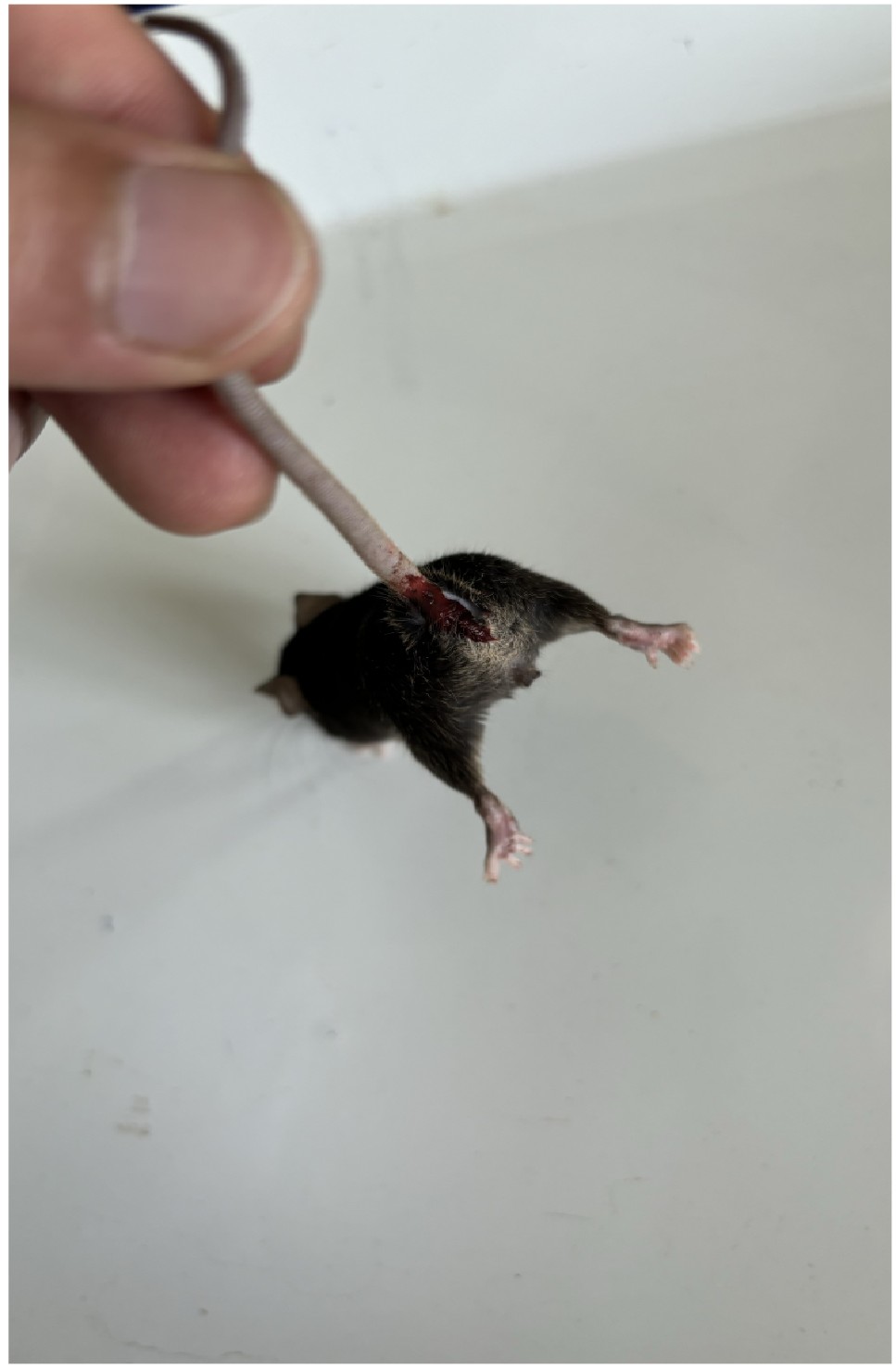

Supplement: Supplementary file 4 [file DataSheet4.zip › Figure-3/fig3-B(Original image of mouse colon)/DSS.jpg]

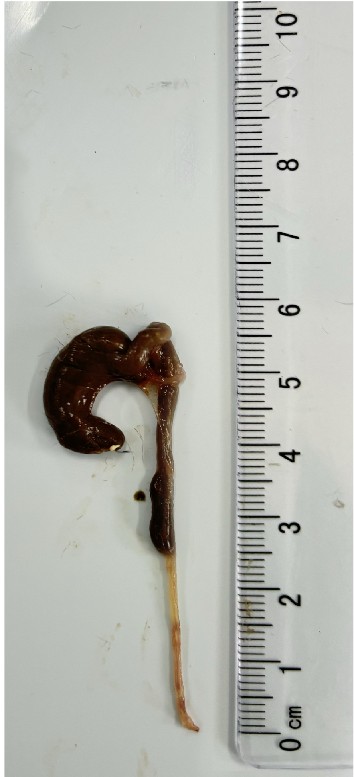

Supplement: Supplementary file 4 [file DataSheet4.zip › Figure-3/fig3-B(Original image of mouse colon)/DSS(1).jpg]

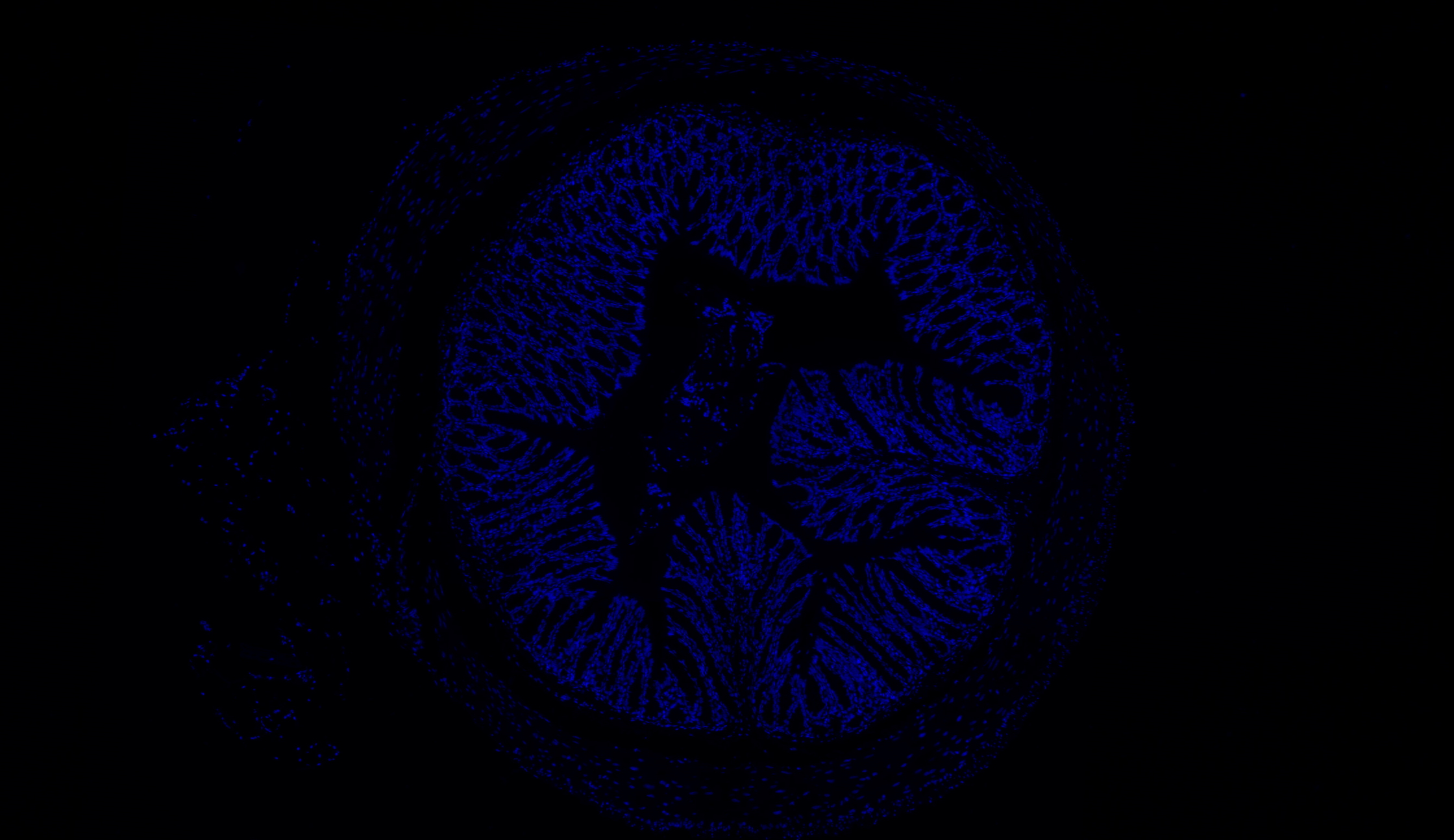

Supplement: Supplementary file 6 [file DataSheet6.zip › Figure-6/fig6-A(IF Original image)/DAPI/B2 结肠 OCCLUDIN(CY3)+ZO-1(488)+MUC2(CY5)_7.7x.jpg]

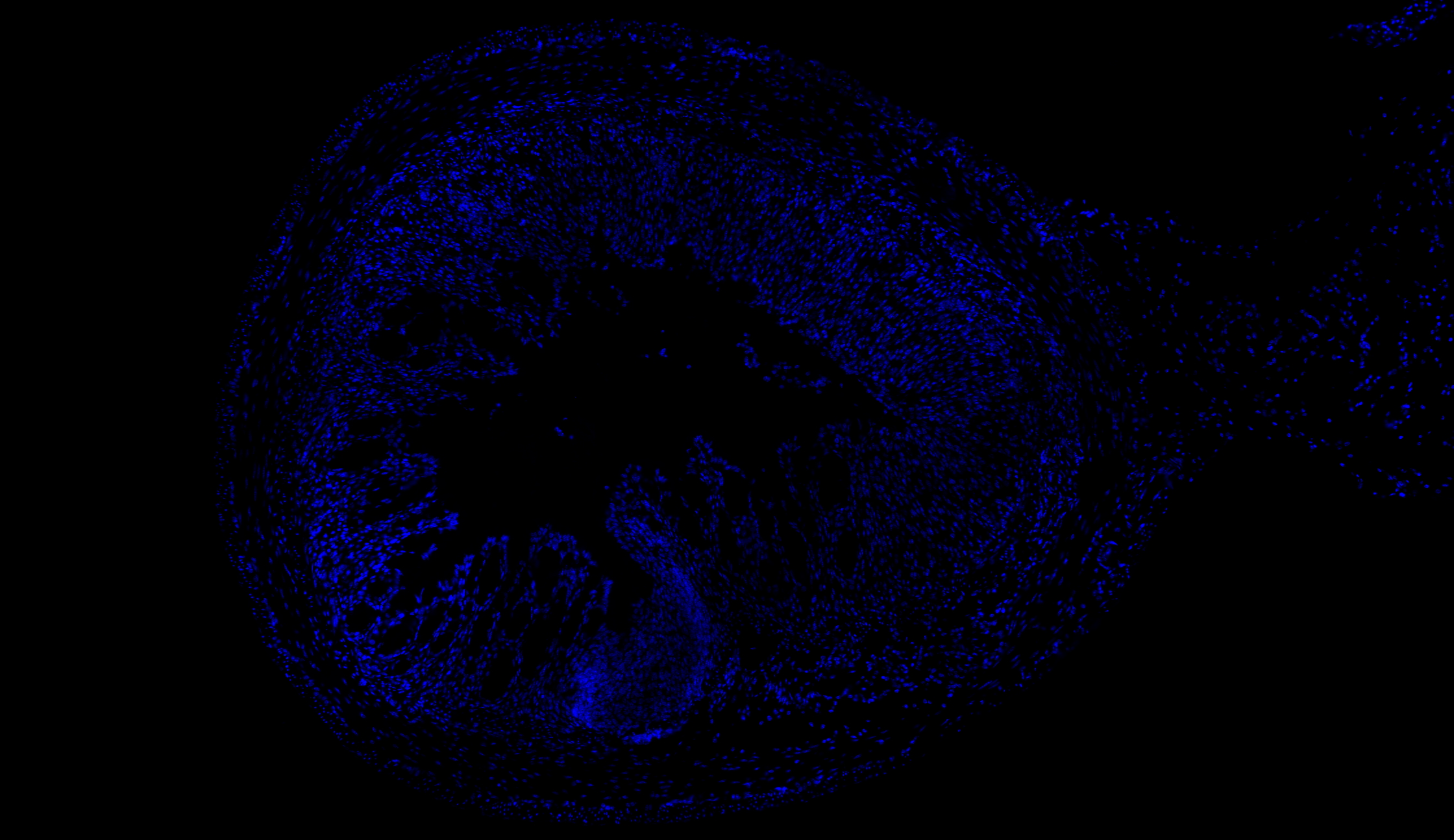

Supplement: Supplementary file 6 [file DataSheet6.zip › Figure-6/fig6-A(IF Original image)/DAPI/DSS-6结肠 ZO-1(488)+OCCLUDIN(CY3)+MUC2(CY5)_9.0x.jpg]

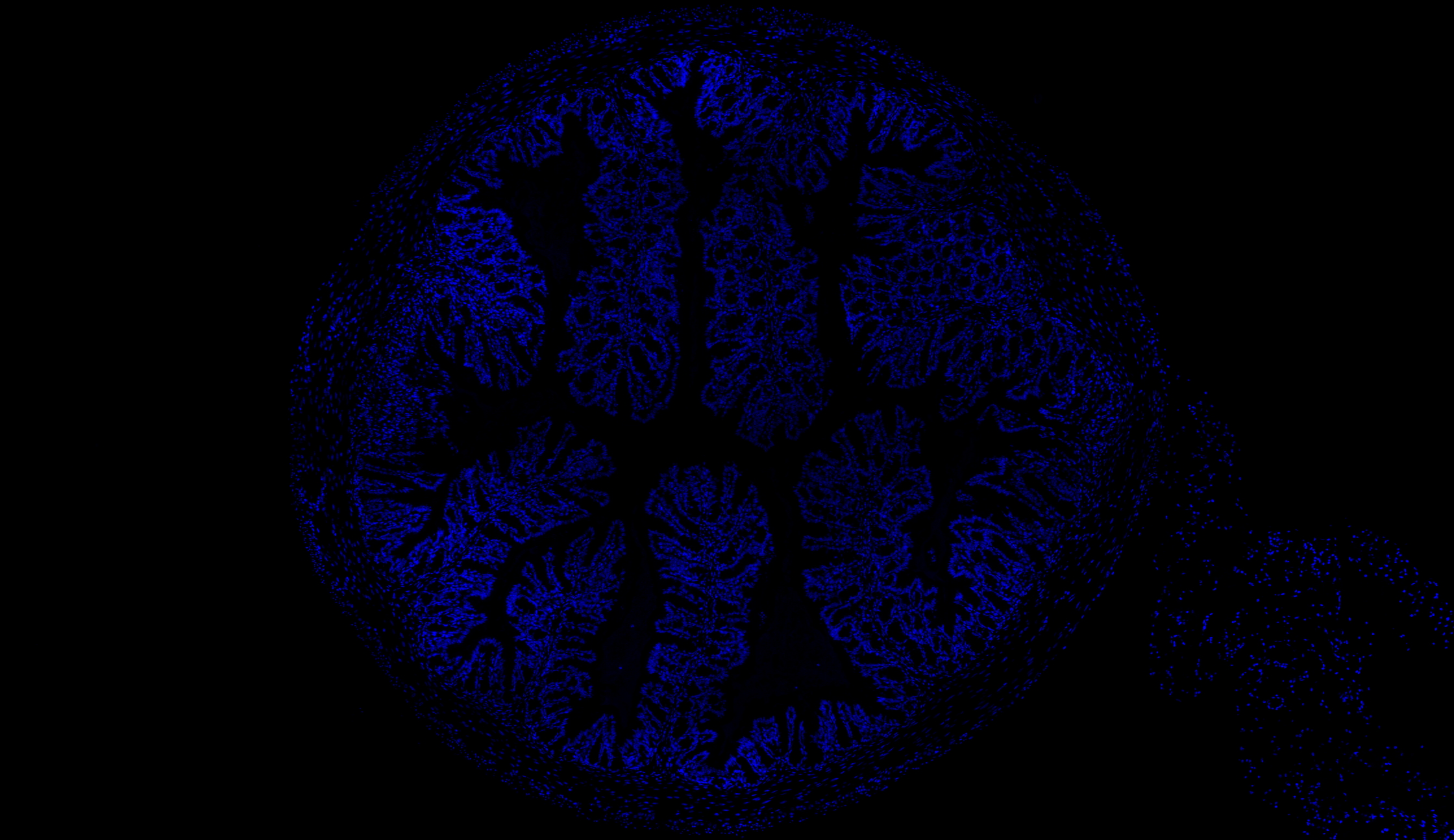

Supplement: Supplementary file 6 [file DataSheet6.zip › Figure-6/fig6-A(IF Original image)/DAPI/H-J-5 ZO-1(488)+OCCLUDIN(CY3)+MUC2(CY5)_6.5x.jpg]

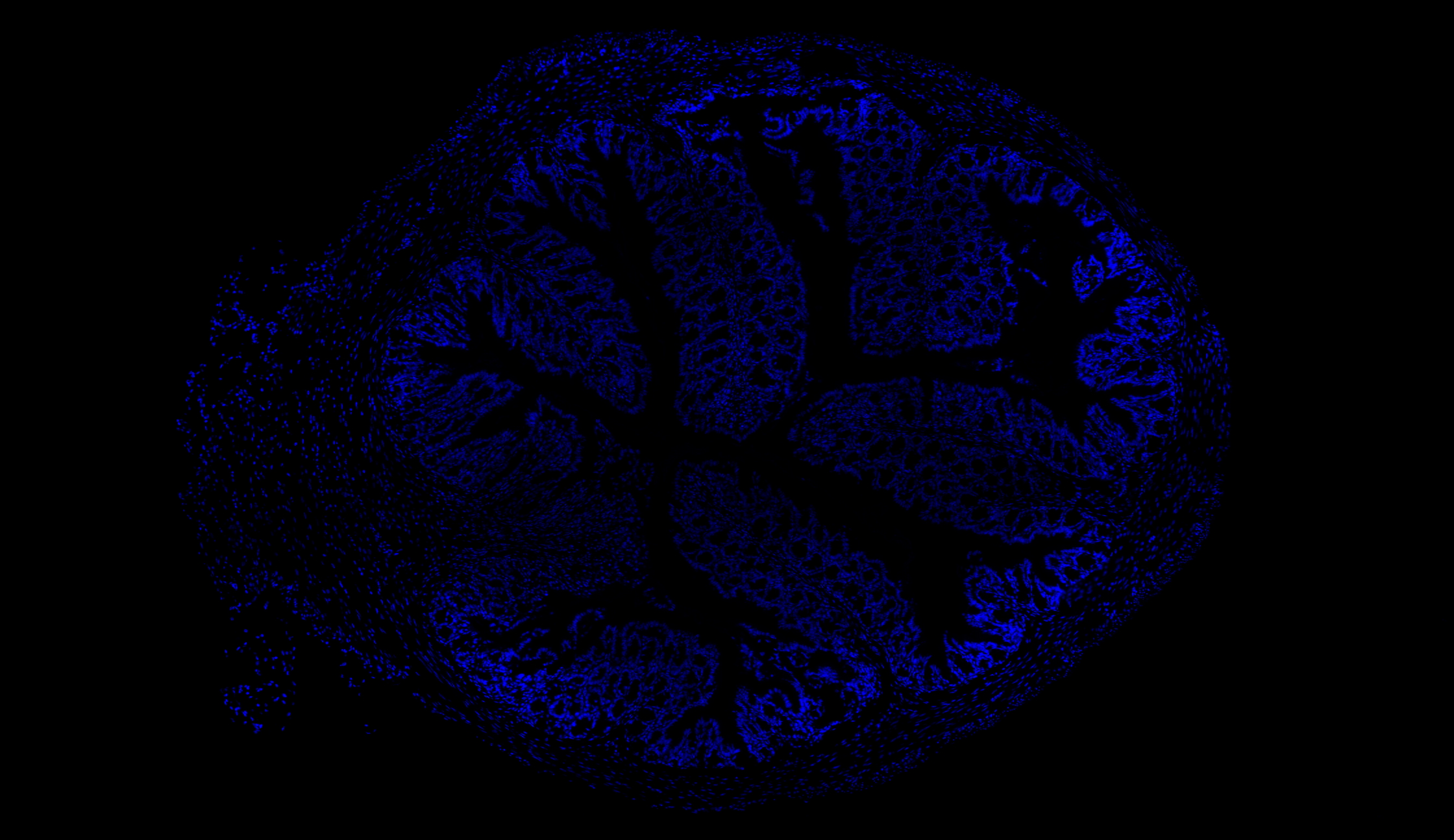

Supplement: Supplementary file 6 [file DataSheet6.zip › Figure-6/fig6-A(IF Original image)/DAPI/Y1-J-5 ZO-1(488)+OCCLUDIN(CY3)+MUC2(CY5)_7.2x.jpg]

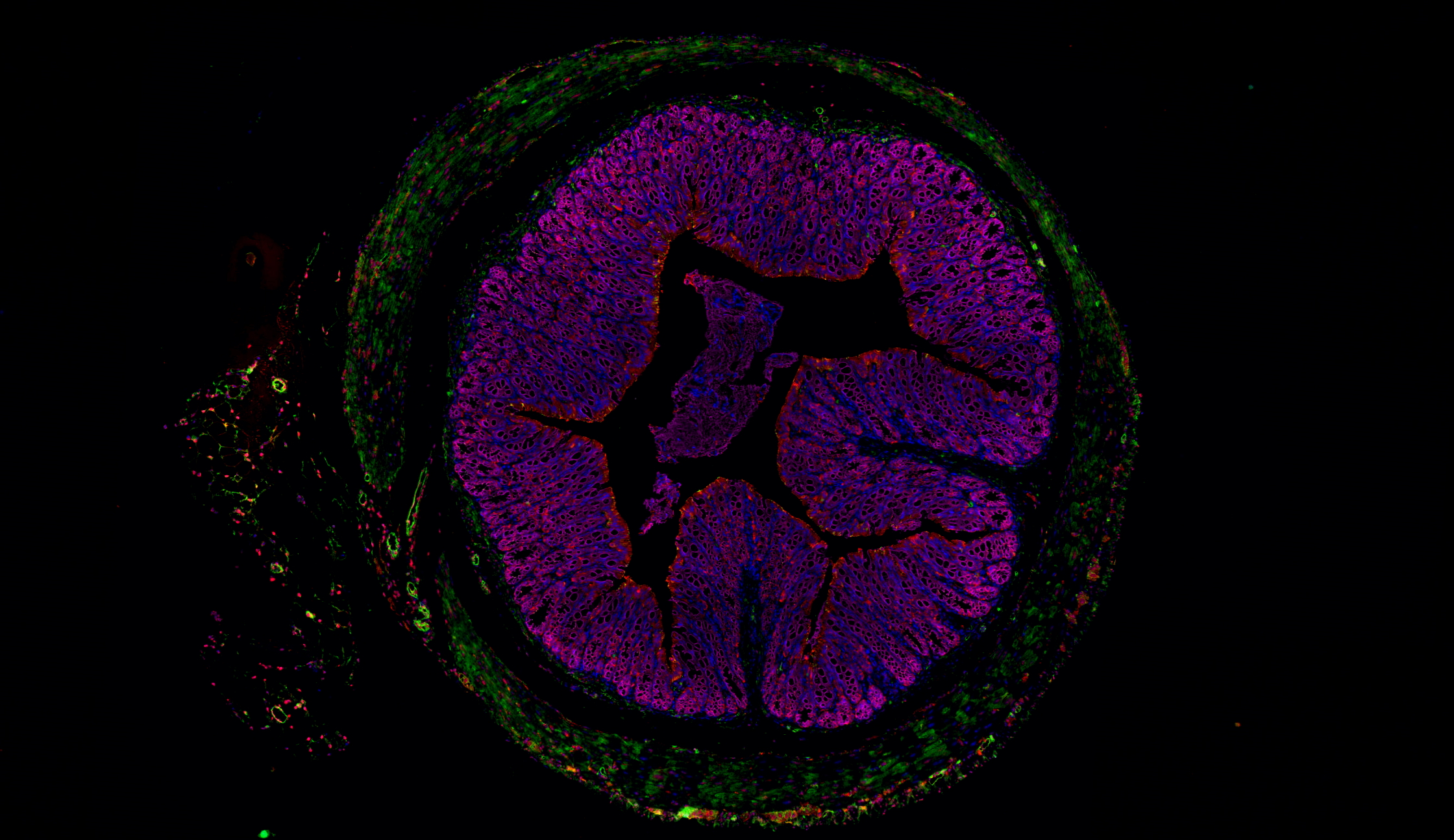

Supplement: Supplementary file 6 [file DataSheet6.zip › Figure-6/fig6-A(IF Original image)/mergy/B2 结肠 OCCLUDIN(CY3)+ZO-1(488)+MUC2(CY5)_8.1x.jpg]

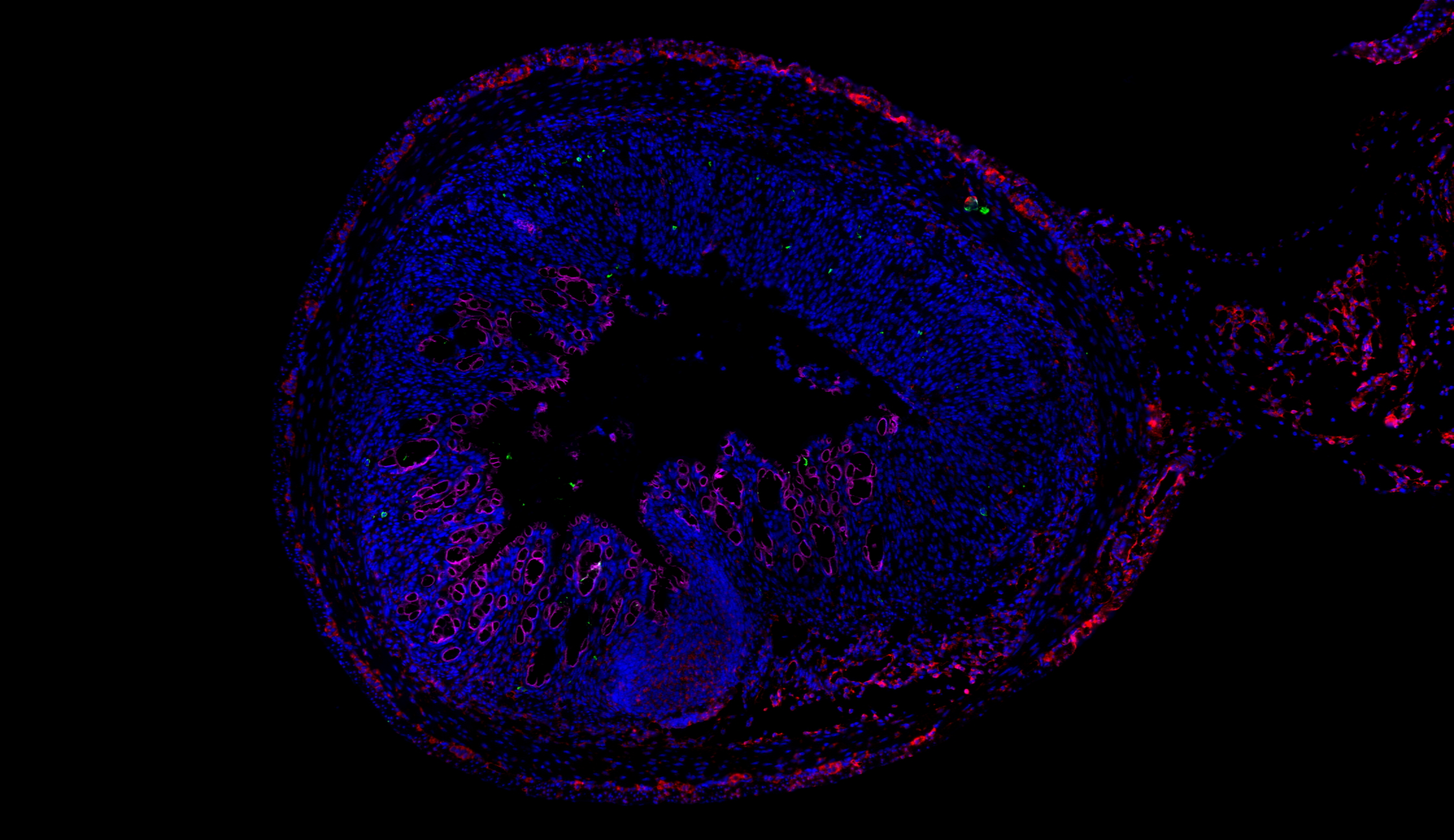

Supplement: Supplementary file 6 [file DataSheet6.zip › Figure-6/fig6-A(IF Original image)/mergy/DSS-6结肠 ZO-1(488)+OCCLUDIN(CY3)+MUC2(CY5)_8.8x.jpg]

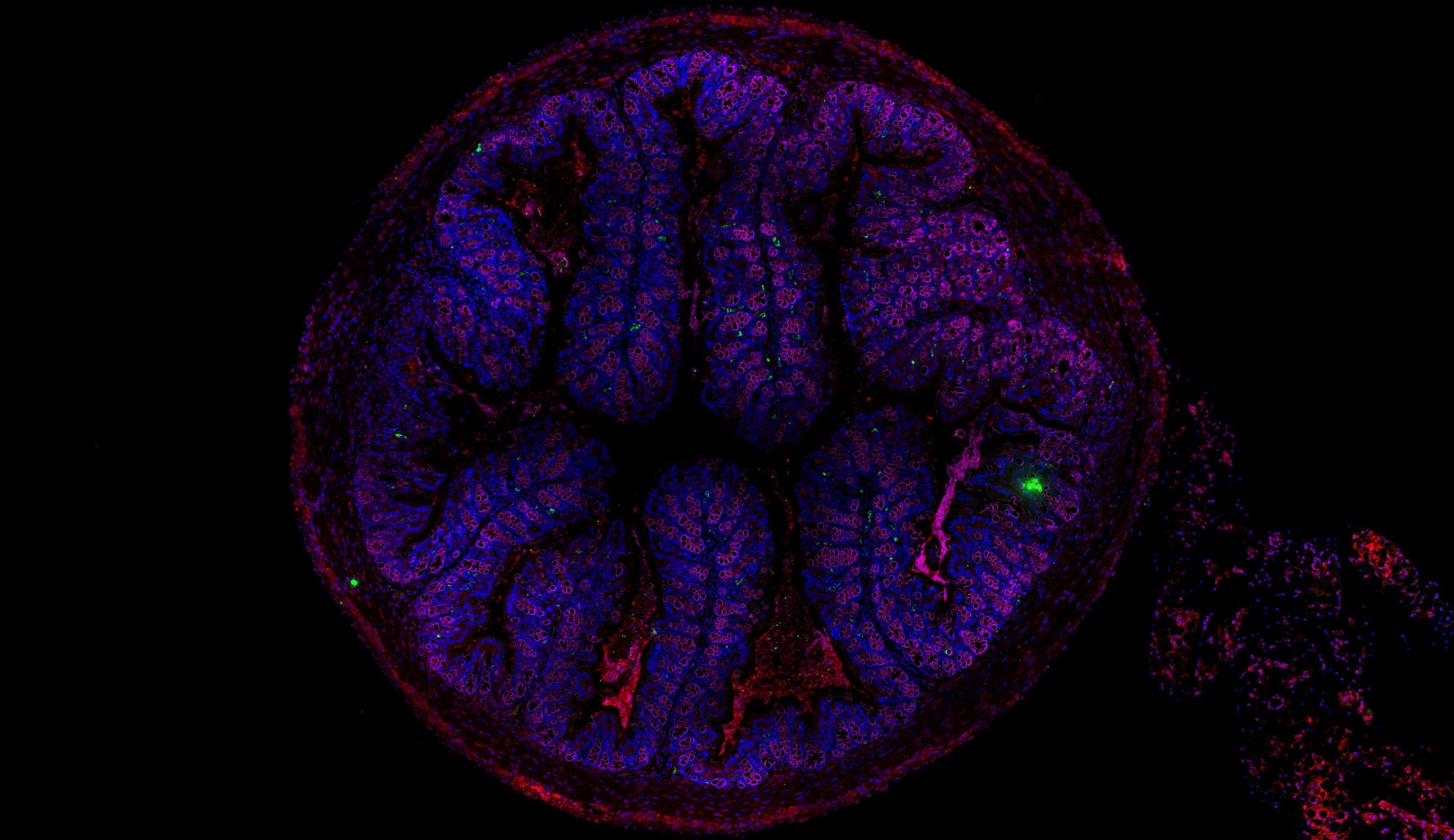

Supplement: Supplementary file 6 [file DataSheet6.zip › Figure-6/fig6-A(IF Original image)/mergy/H-J-5 ZO-1(488)+OCCLUDIN(CY3)+MUC2(CY5)_6.5x.jpg]

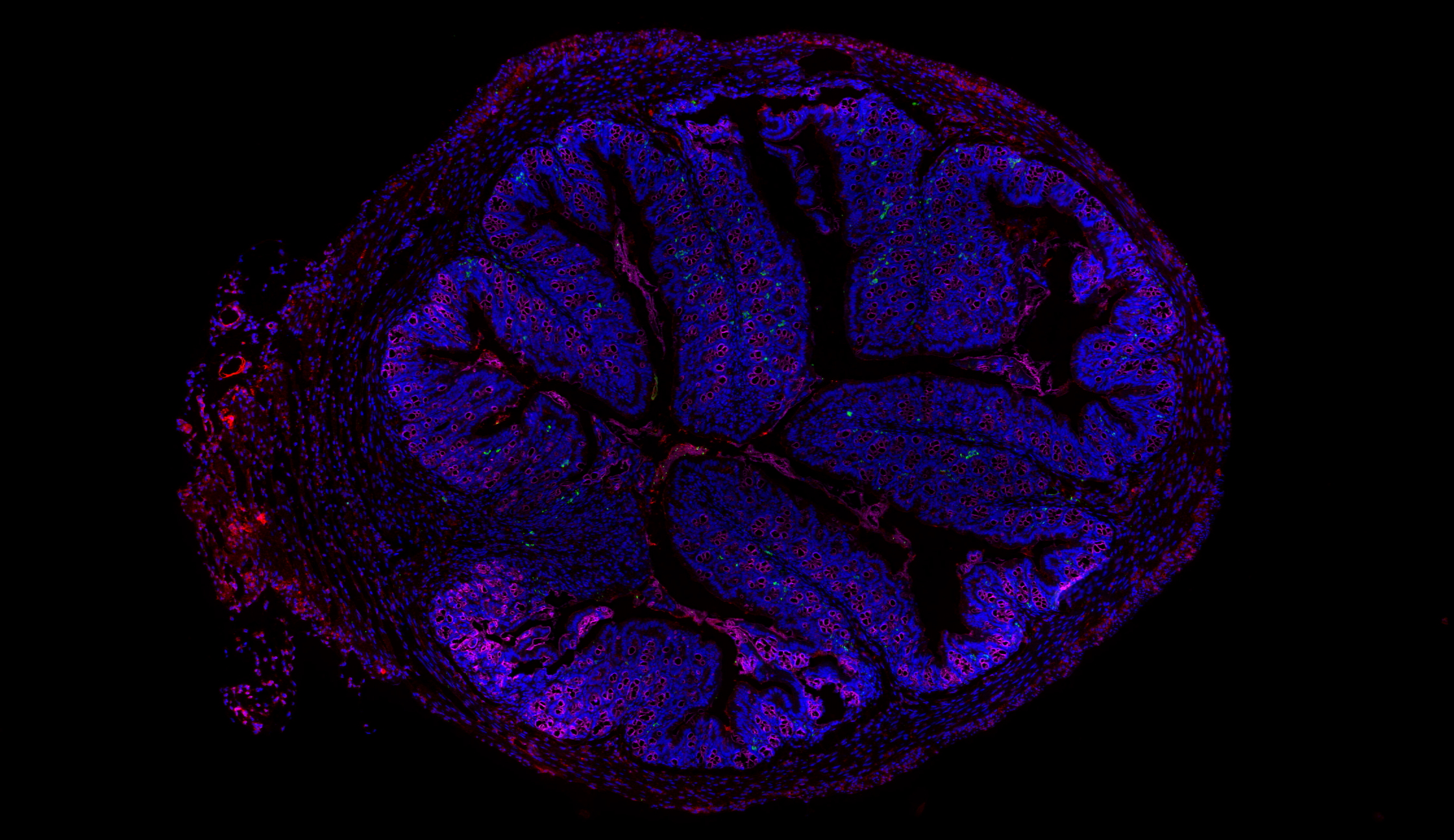

Supplement: Supplementary file 6 [file DataSheet6.zip › Figure-6/fig6-A(IF Original image)/mergy/Y1-J-5 ZO-1(488)+OCCLUDIN(CY3)+MUC2(CY5)_7.2x.jpg]

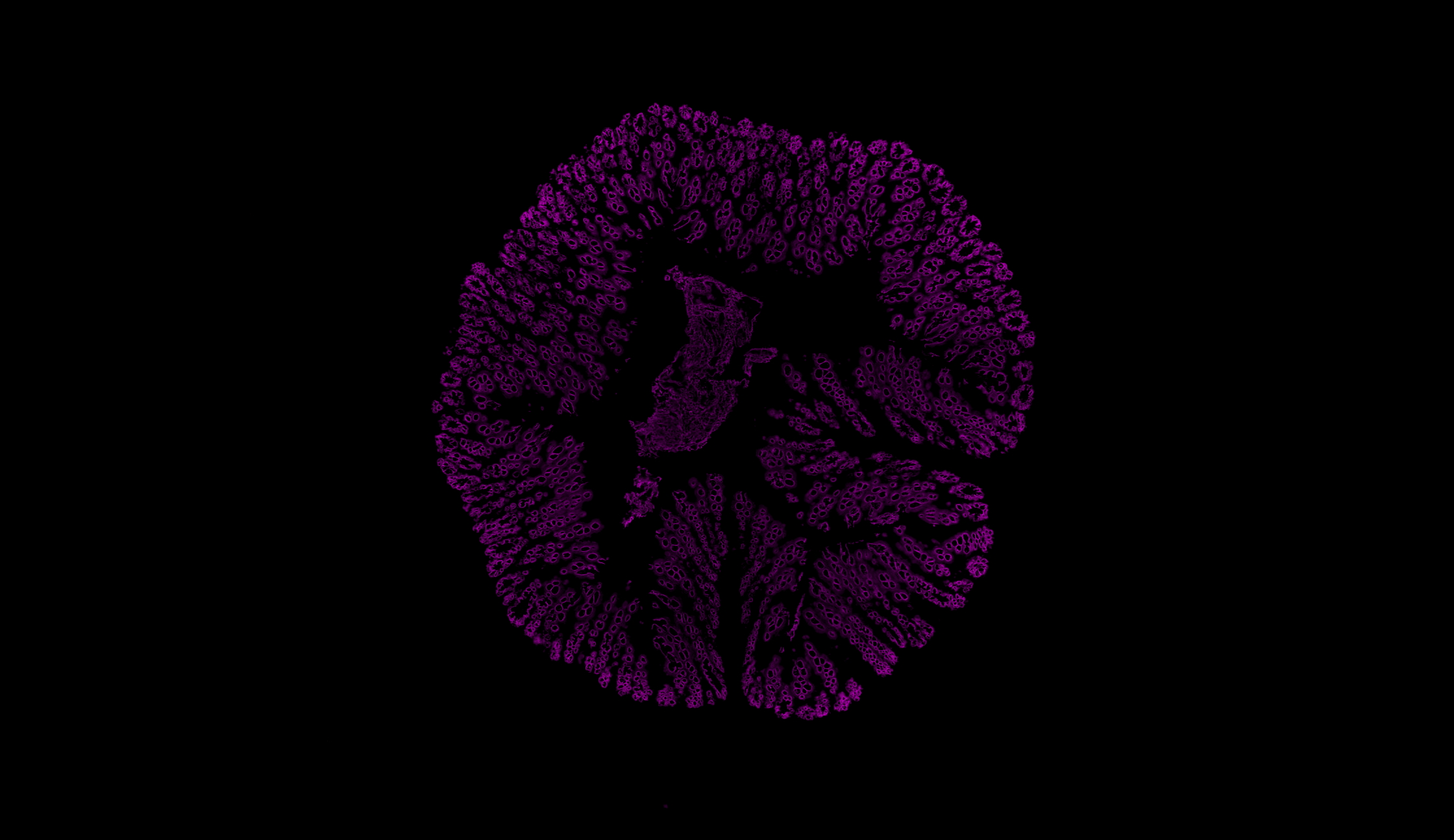

Supplement: Supplementary file 6 [file DataSheet6.zip › Figure-6/fig6-A(IF Original image)/muc2/B2 结肠 OCCLUDIN(CY3)+ZO-1(488)+MUC2(CY5)_8.1x.jpg]

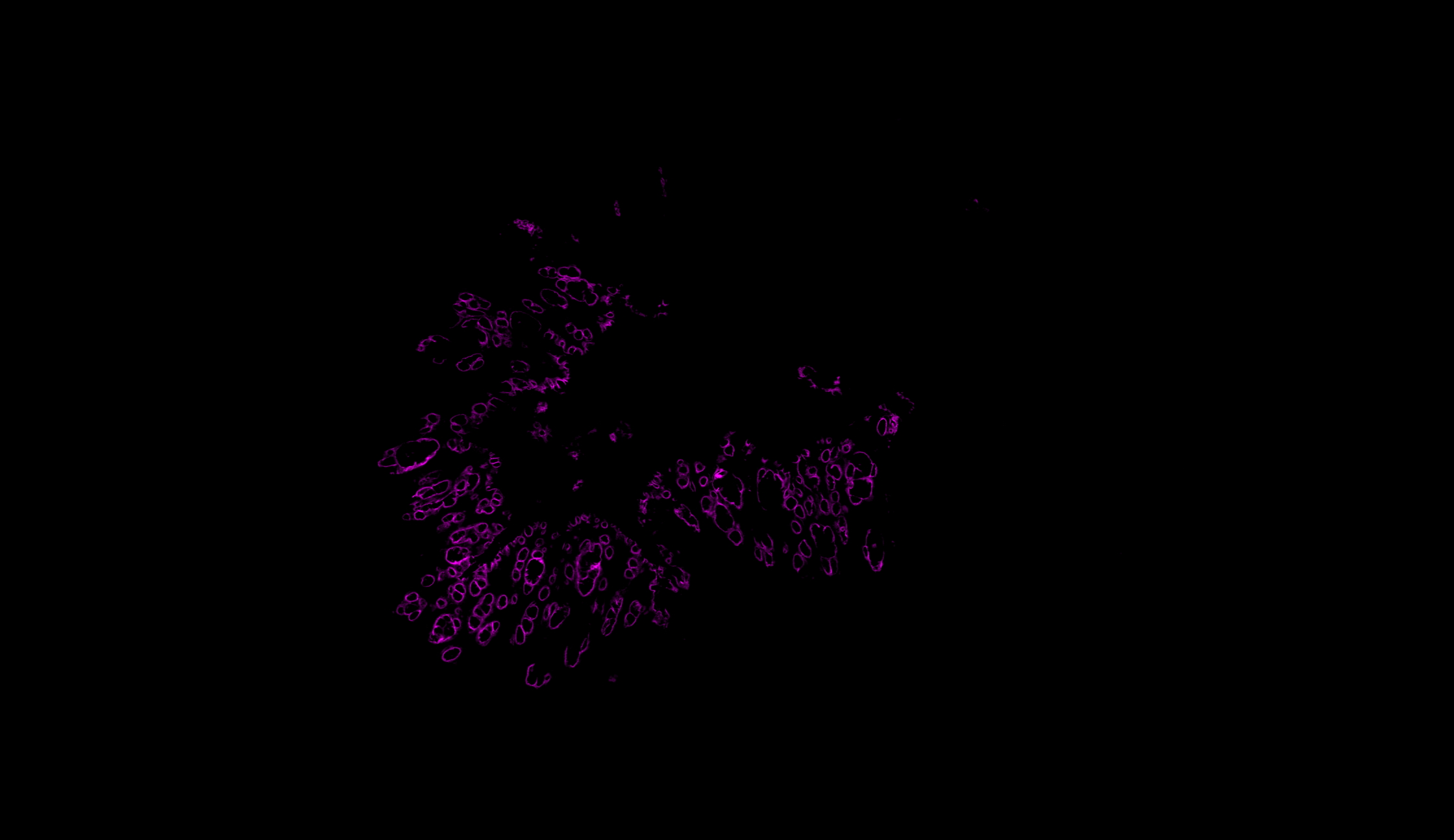

Supplement: Supplementary file 6 [file DataSheet6.zip › Figure-6/fig6-A(IF Original image)/muc2/DSS-6结肠 ZO-1(488)+OCCLUDIN(CY3)+MUC2(CY5)_8.8x.jpg]

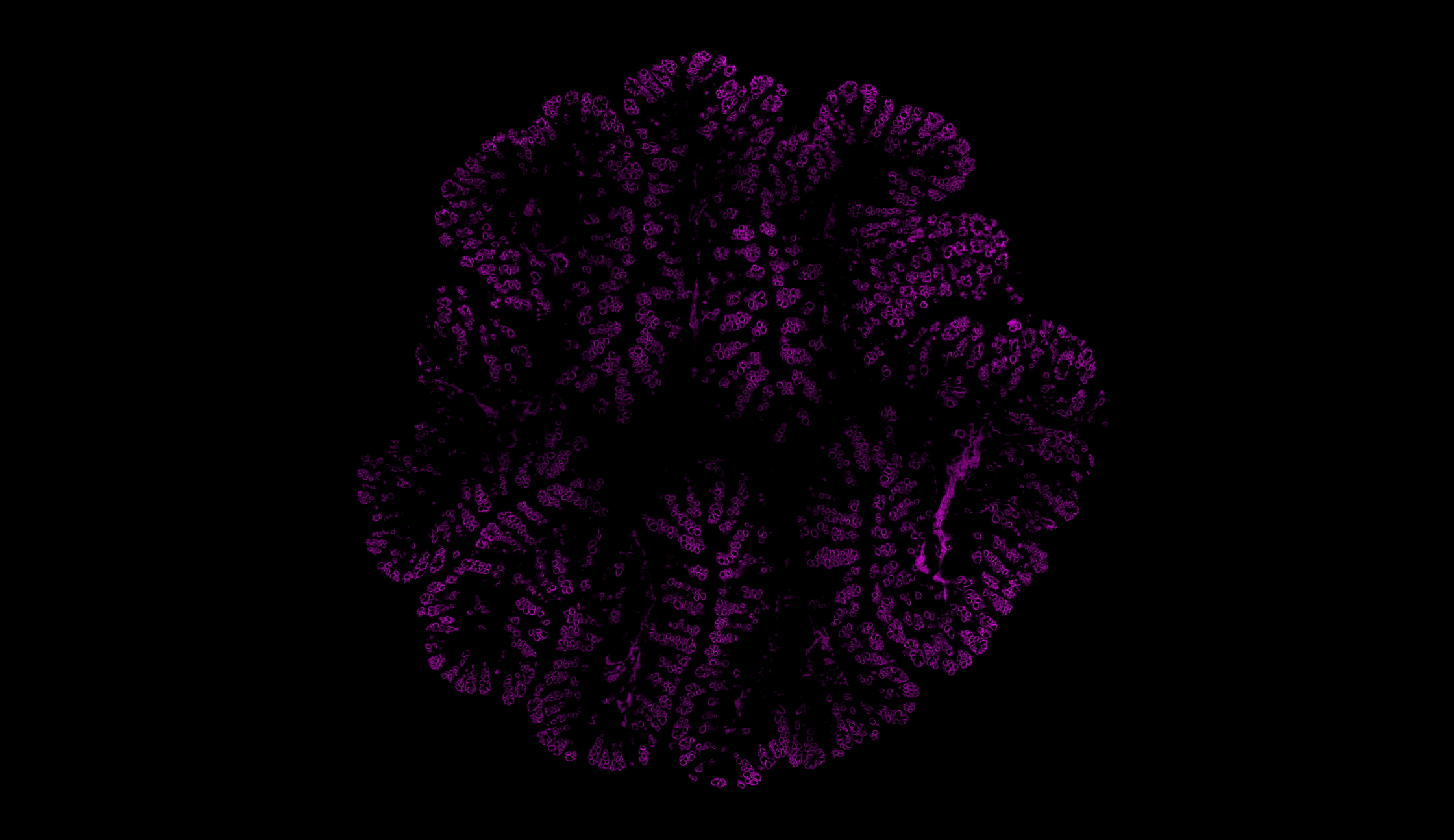

Supplement: Supplementary file 6 [file DataSheet6.zip › Figure-6/fig6-A(IF Original image)/muc2/H-J-5 ZO-1(488)+OCCLUDIN(CY3)+MUC2(CY5)_6.5x.jpg]

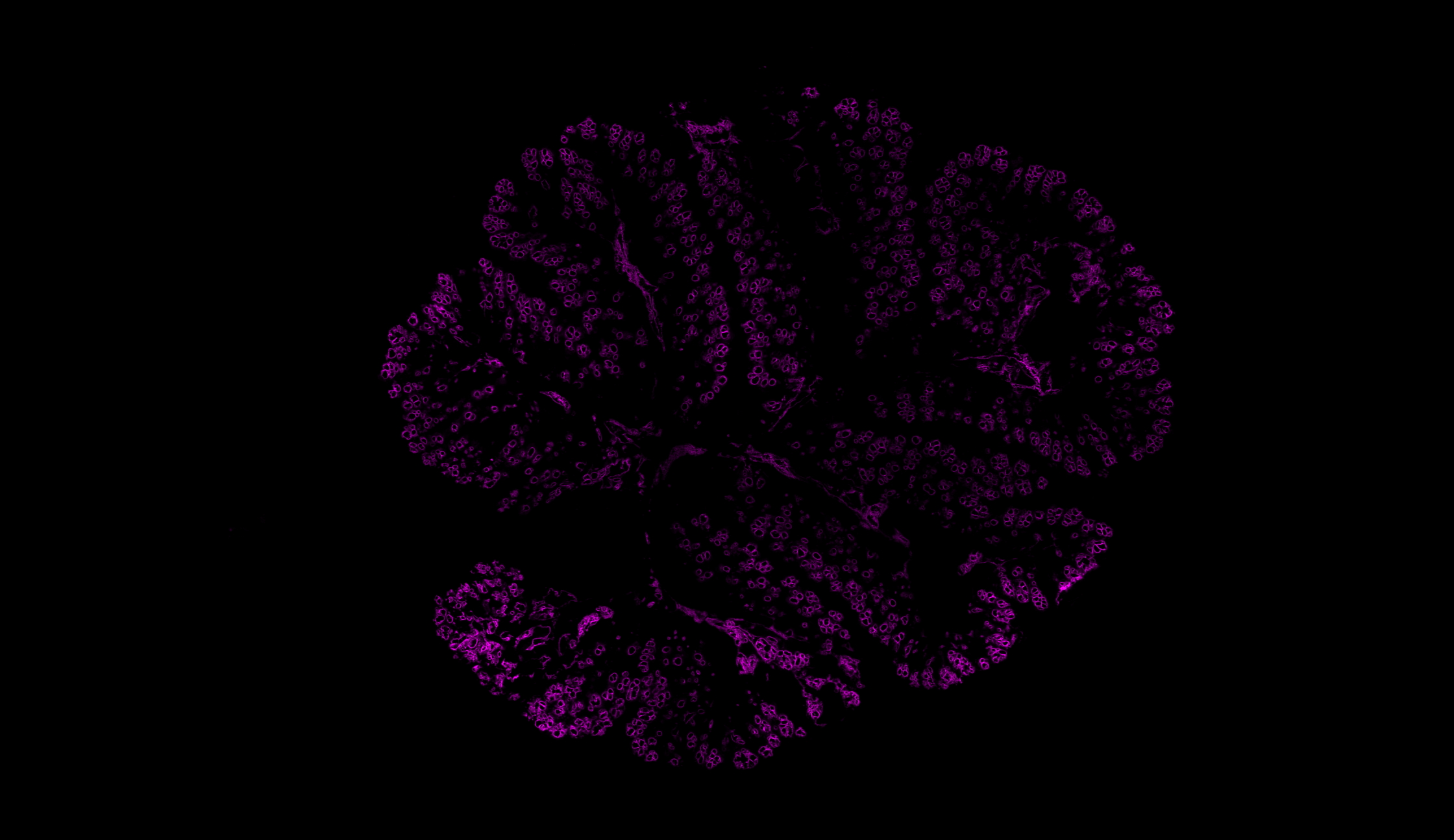

Supplement: Supplementary file 6 [file DataSheet6.zip › Figure-6/fig6-A(IF Original image)/muc2/Y1-J-5 ZO-1(488)+OCCLUDIN(CY3)+MUC2(CY5)_7.2x.jpg]

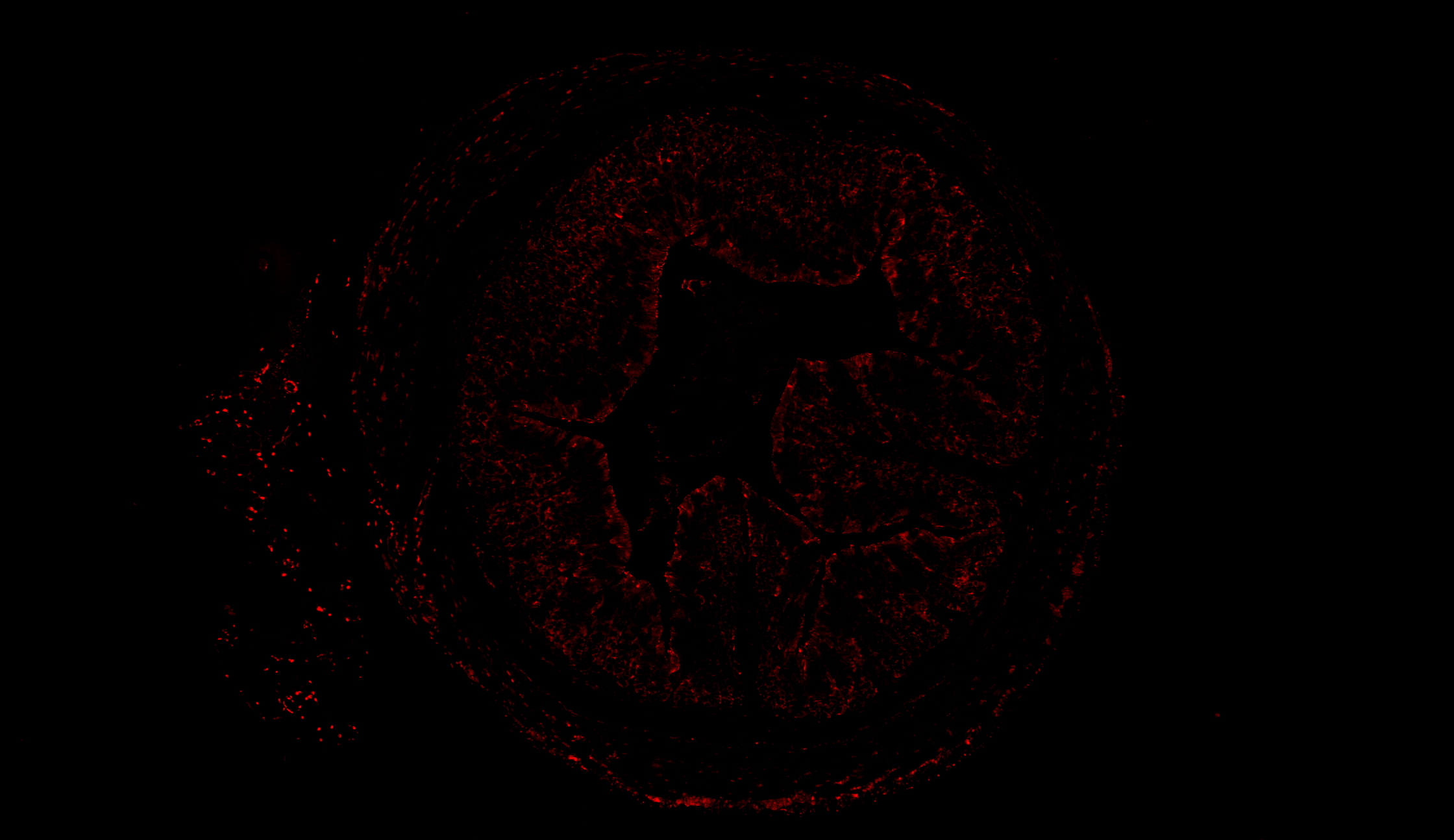

Supplement: Supplementary file 6 [file DataSheet6.zip › Figure-6/fig6-A(IF Original image)/occludin/B2 结肠 OCCLUDIN(CY3)+ZO-1(488)+MUC2(CY5)_8.0x.jpg]

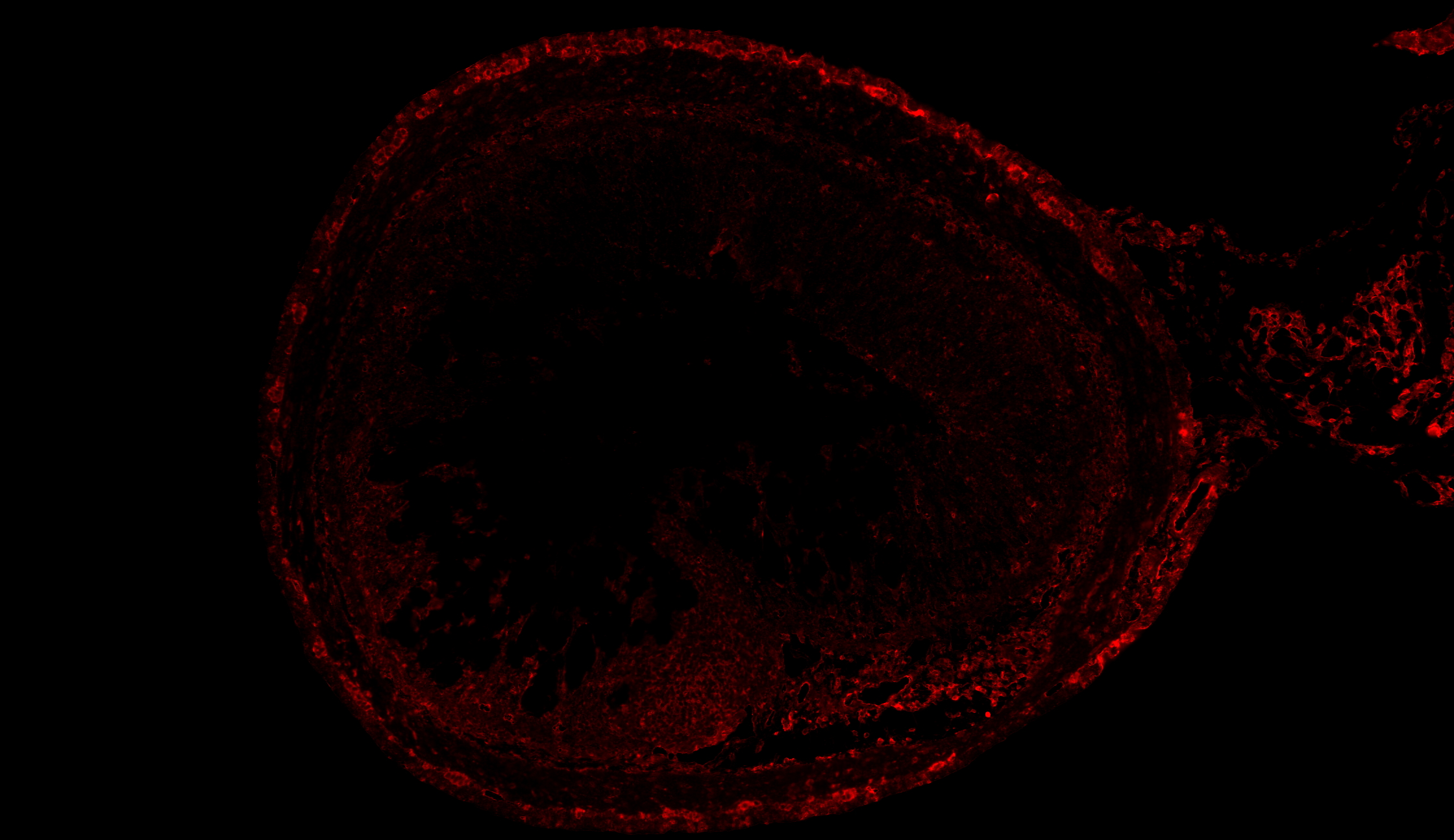

Supplement: Supplementary file 6 [file DataSheet6.zip › Figure-6/fig6-A(IF Original image)/occludin/DSS-6结肠 ZO-1(488)+OCCLUDIN(CY3)+MUC2(CY5)_8.8x.jpg]

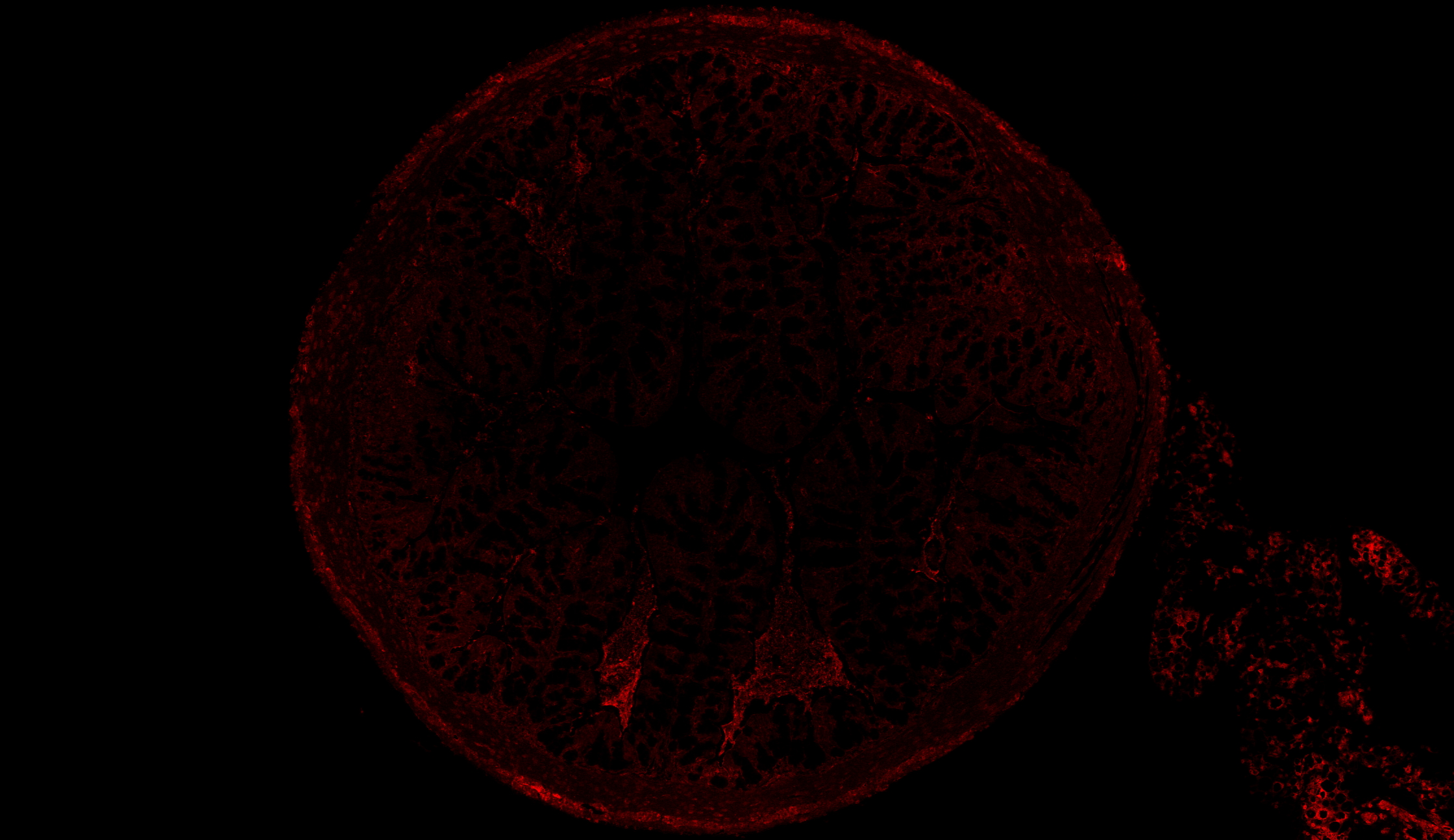

Supplement: Supplementary file 6 [file DataSheet6.zip › Figure-6/fig6-A(IF Original image)/occludin/H-J-5 ZO-1(488)+OCCLUDIN(CY3)+MUC2(CY5)_6.5x.jpg]

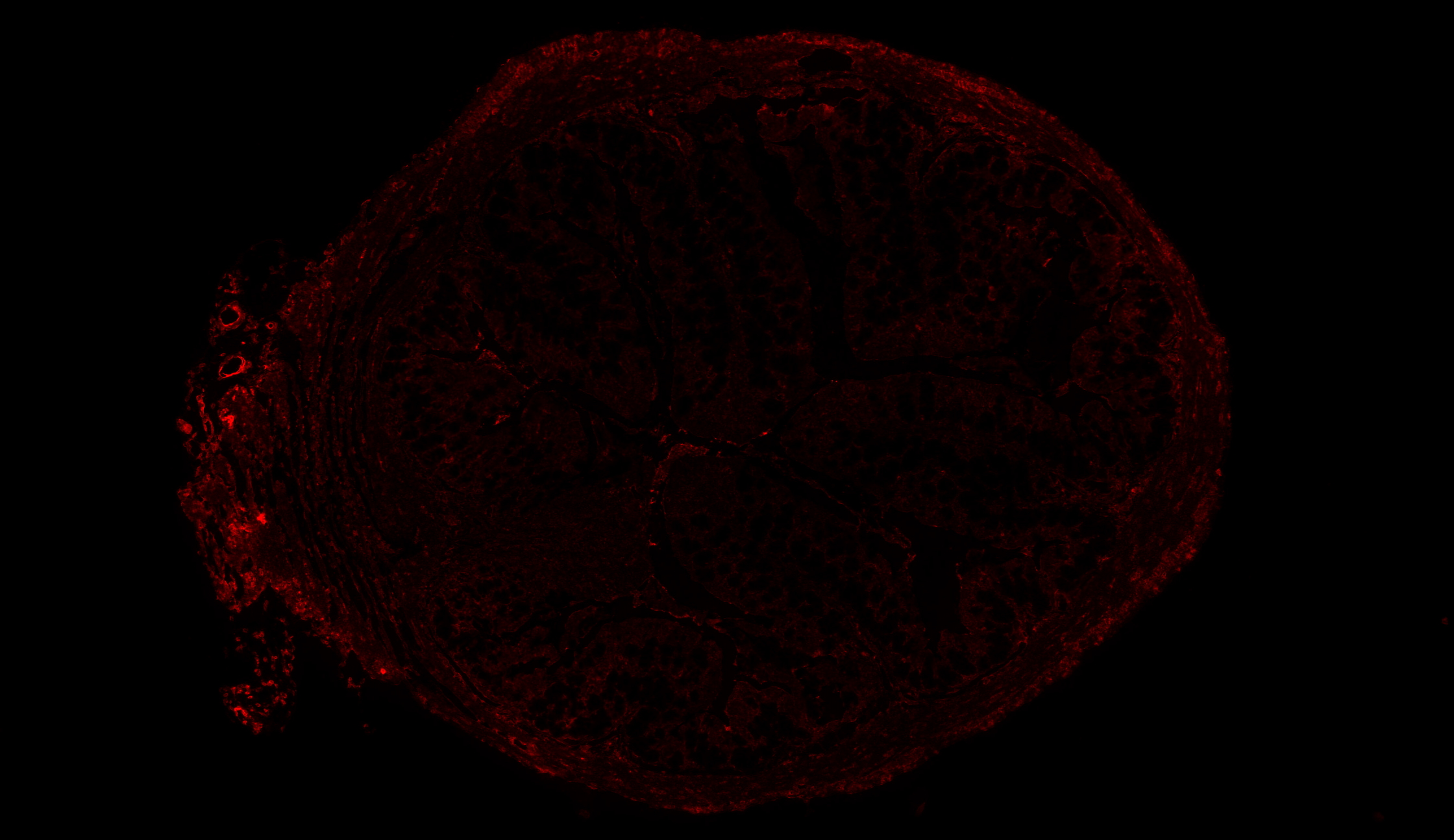

Supplement: Supplementary file 6 [file DataSheet6.zip › Figure-6/fig6-A(IF Original image)/occludin/Y1-J-5 ZO-1(488)+OCCLUDIN(CY3)+MUC2(CY5)_7.2x.jpg]

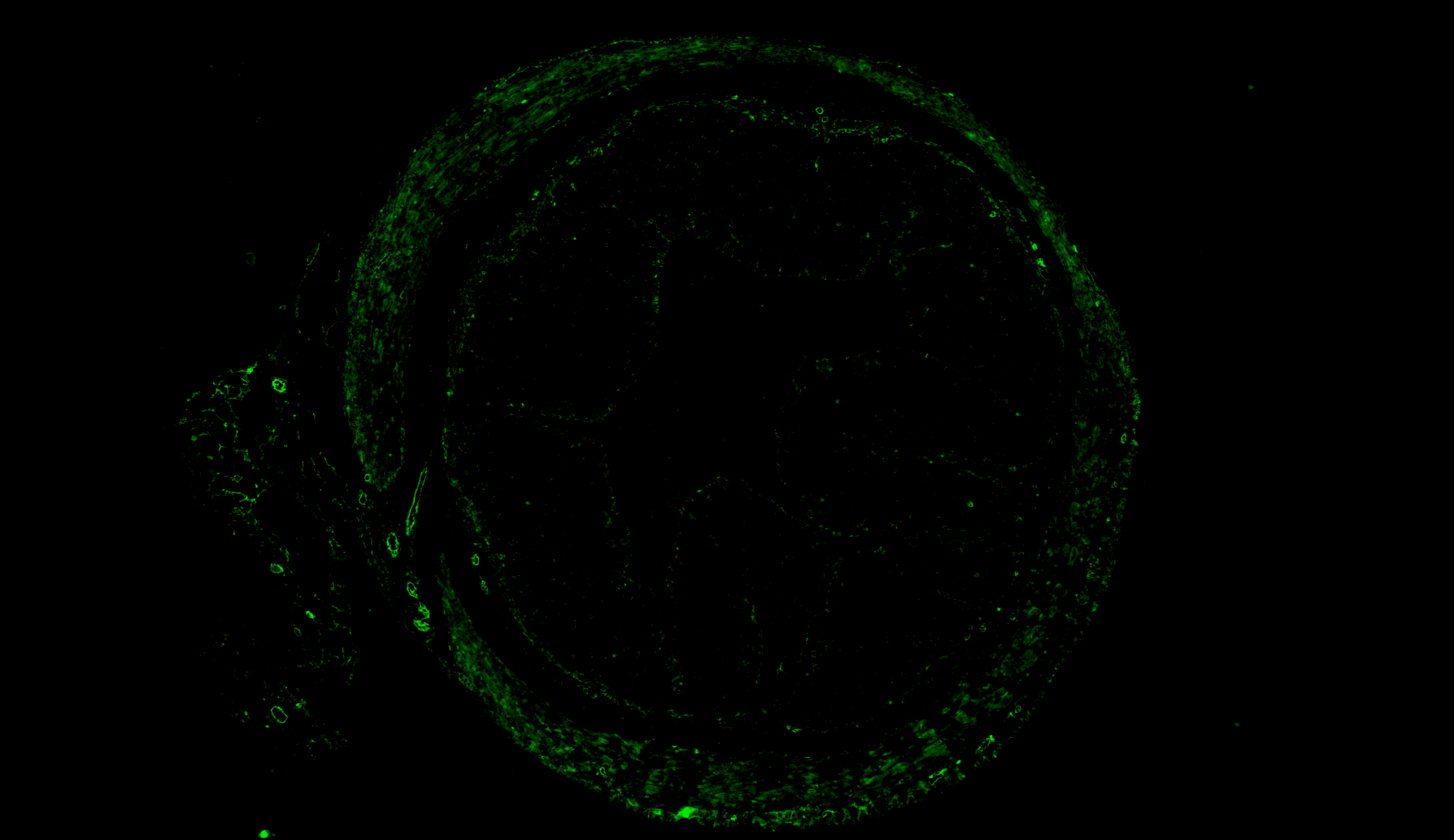

Supplement: Supplementary file 6 [file DataSheet6.zip › Figure-6/fig6-A(IF Original image)/zo-1/B2 结肠 OCCLUDIN(CY3)+ZO-1(488)+MUC2(CY5)_8.1x.jpg]

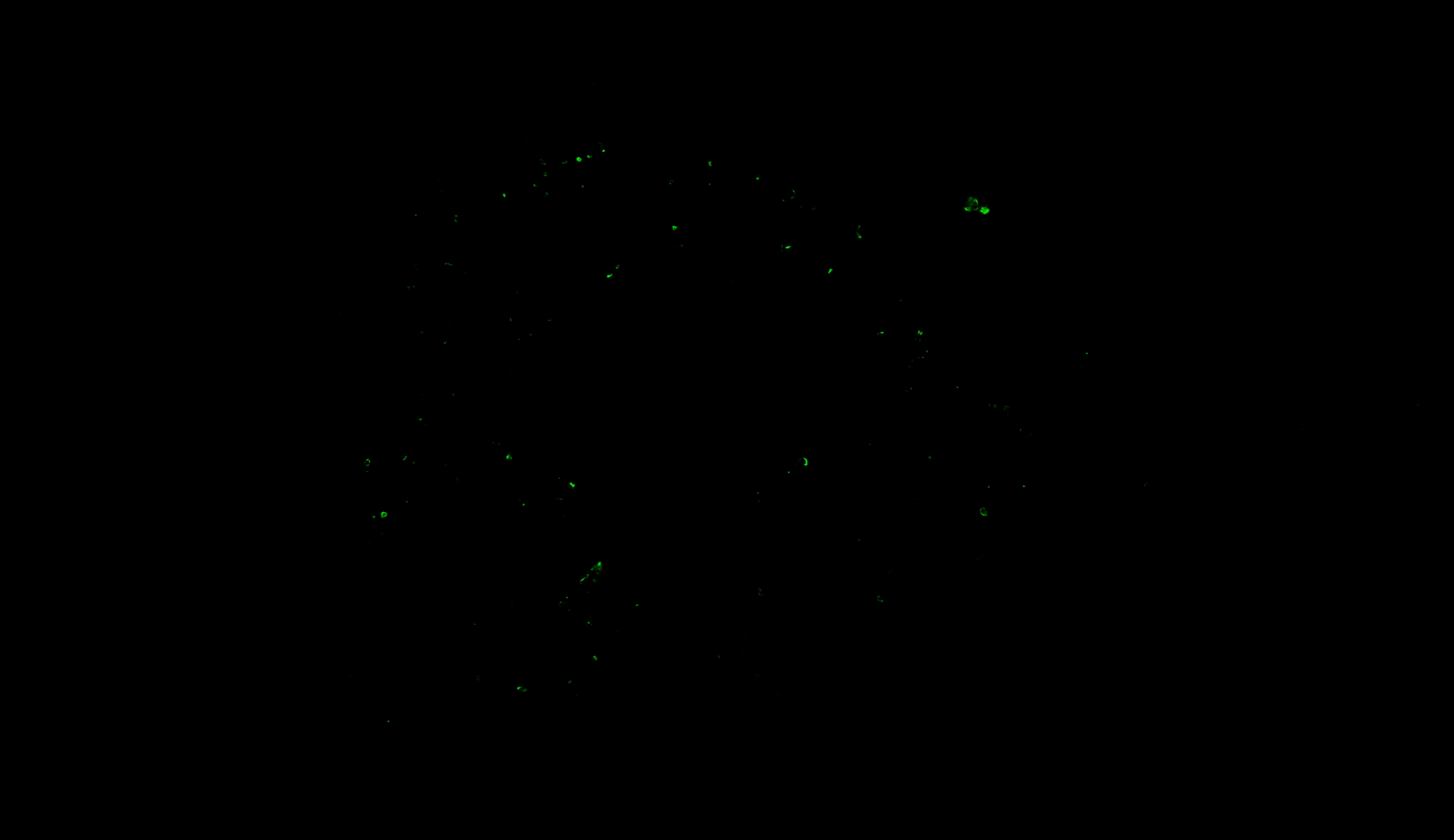

Supplement: Supplementary file 6 [file DataSheet6.zip › Figure-6/fig6-A(IF Original image)/zo-1/DSS-6结肠 ZO-1(488)+OCCLUDIN(CY3)+MUC2(CY5)_9.0x.jpg]

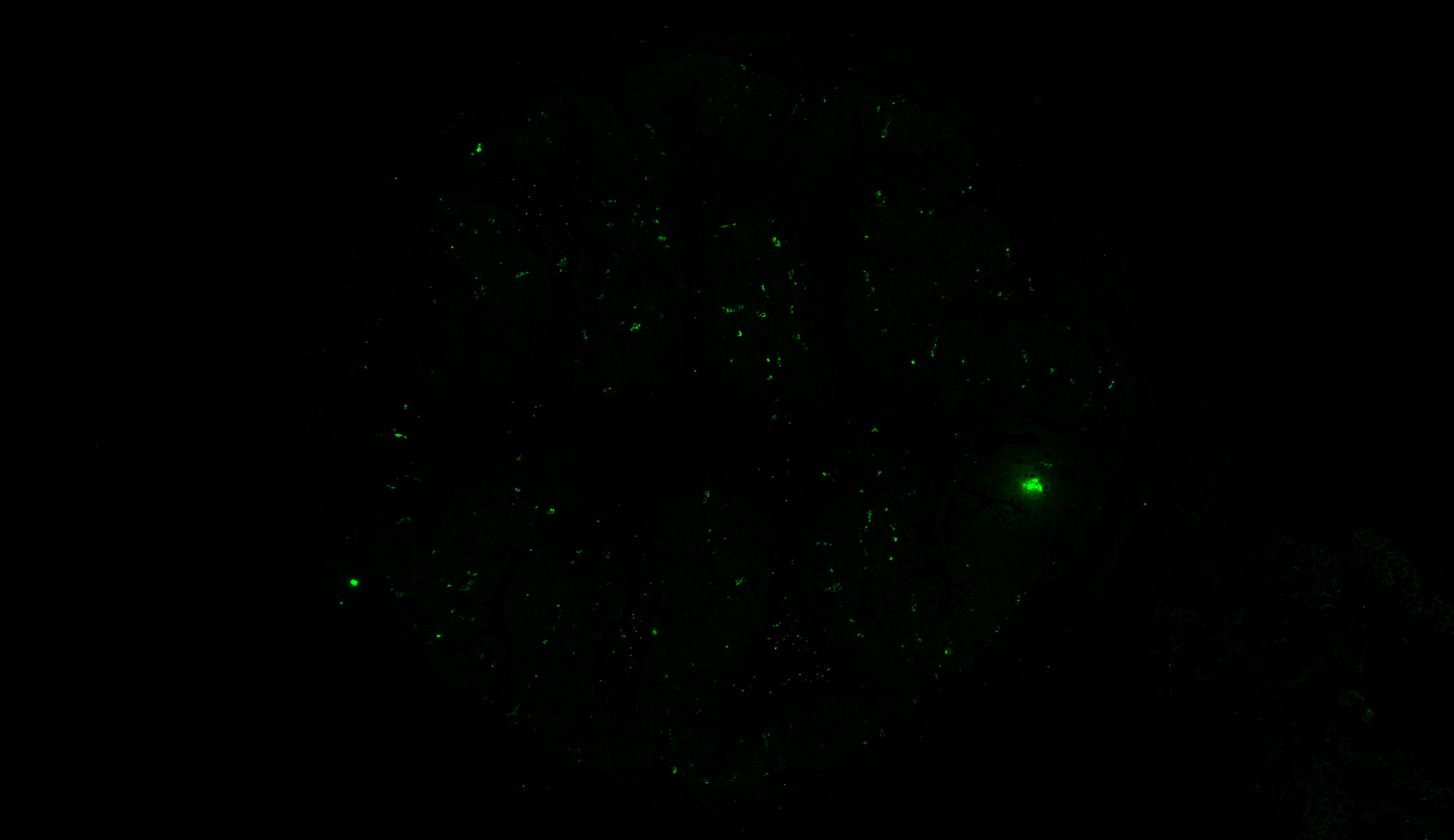

Supplement: Supplementary file 6 [file DataSheet6.zip › Figure-6/fig6-A(IF Original image)/zo-1/H-J-5 ZO-1(488)+OCCLUDIN(CY3)+MUC2(CY5)_6.5x.jpg]

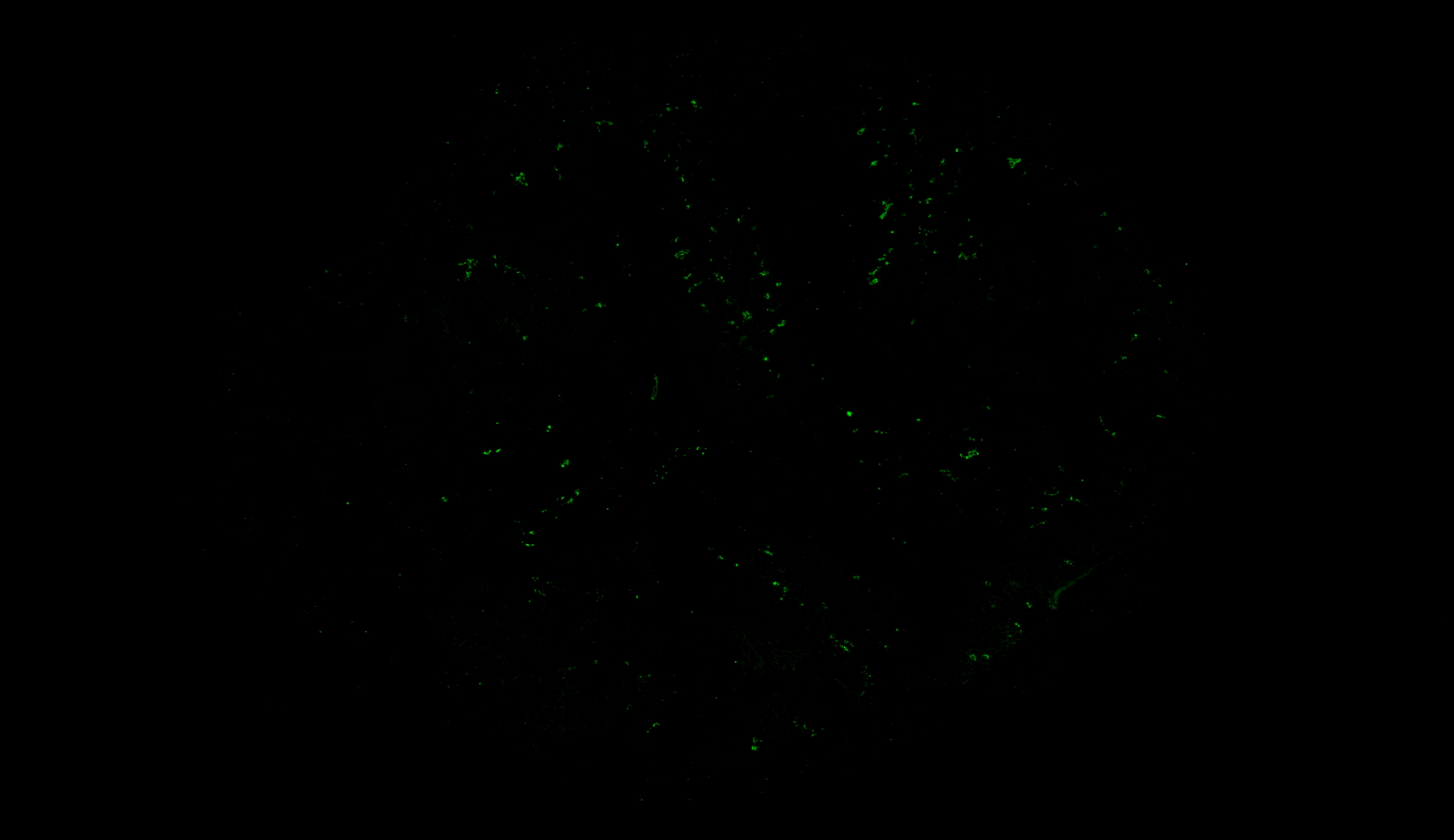

Supplement: Supplementary file 6 [file DataSheet6.zip › Figure-6/fig6-A(IF Original image)/zo-1/Y1-J-5 ZO-1(488)+OCCLUDIN(CY3)+MUC2(CY5)_7.2x.jpg]

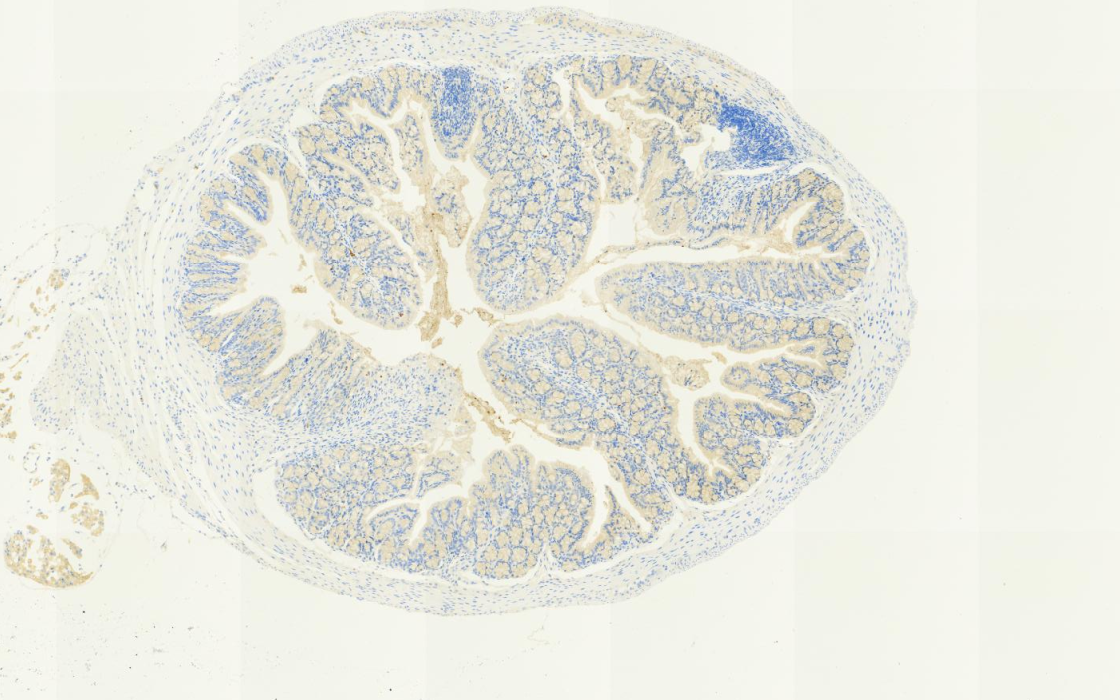

Supplement: Supplementary file 6 [file DataSheet6.zip › Figure-6/fig6-E(IHC Original image)/5-ASA-MUC2_viewcapture.png]

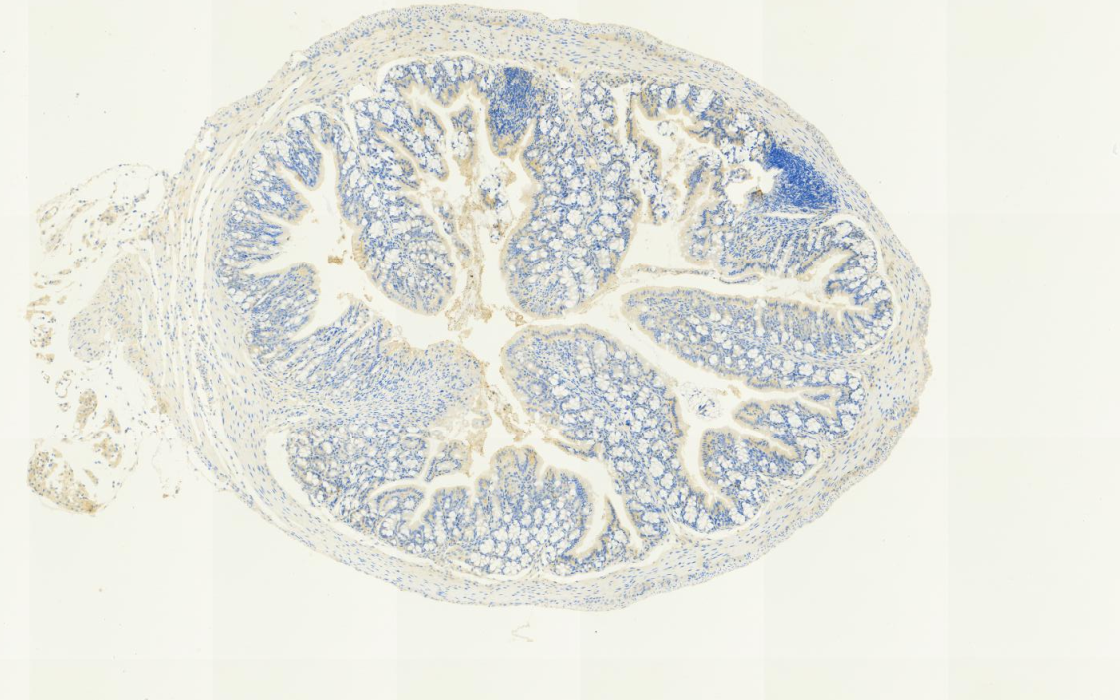

Supplement: Supplementary file 6 [file DataSheet6.zip › Figure-6/fig6-E(IHC Original image)/5-ASA-occludin_viewcapture.png]

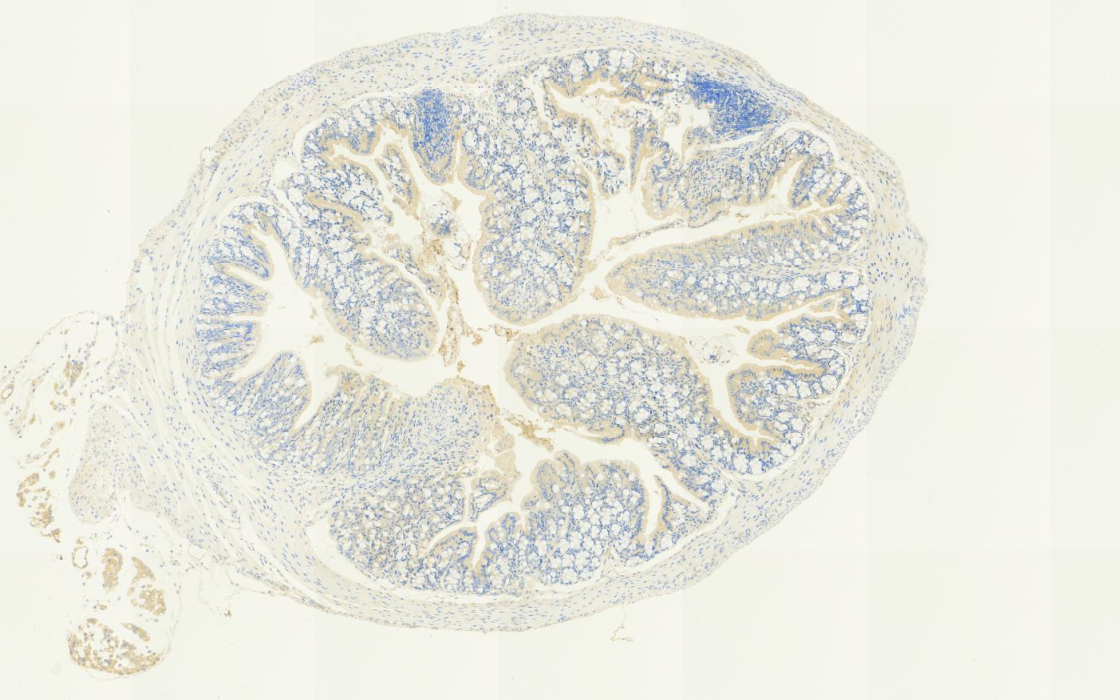

Supplement: Supplementary file 6 [file DataSheet6.zip › Figure-6/fig6-E(IHC Original image)/5-ASA-ZO-1_viewcapture.png]

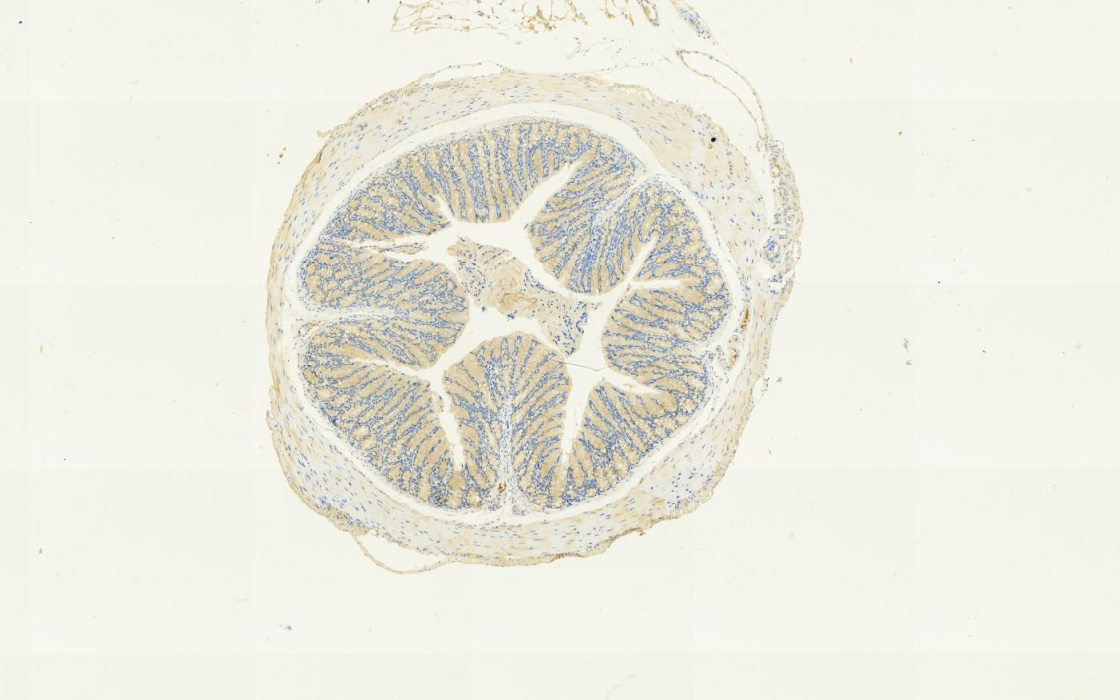

Supplement: Supplementary file 6 [file DataSheet6.zip › Figure-6/fig6-E(IHC Original image)/control-MUC2_viewcapture.png]

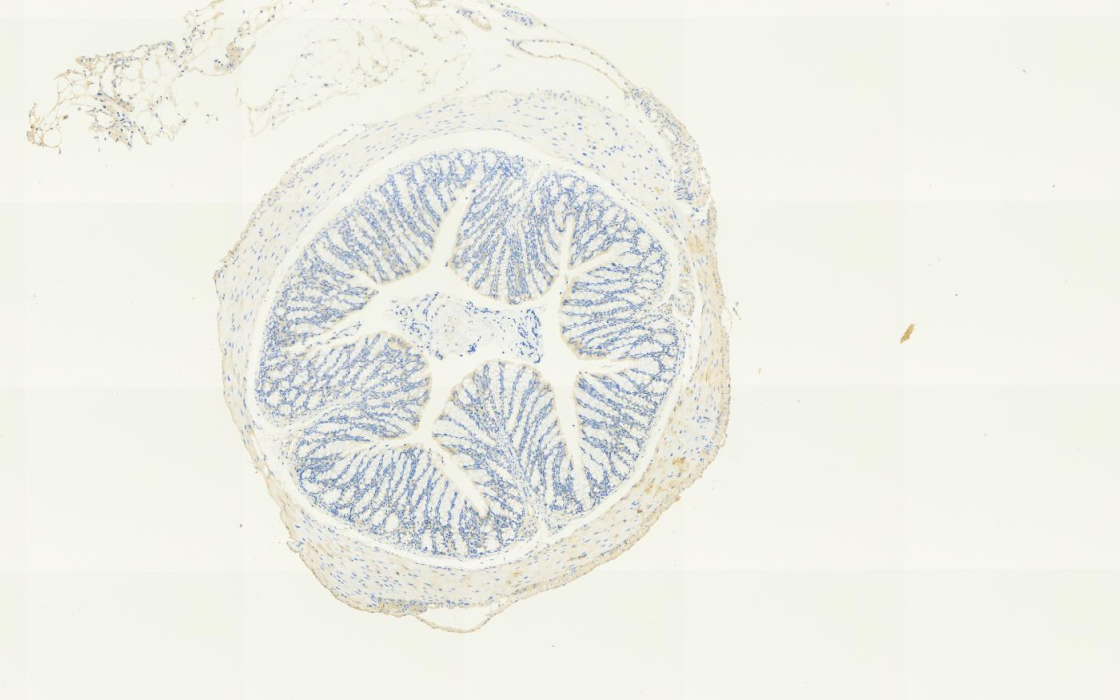

Supplement: Supplementary file 6 [file DataSheet6.zip › Figure-6/fig6-E(IHC Original image)/control-occludin_viewcapture.png]

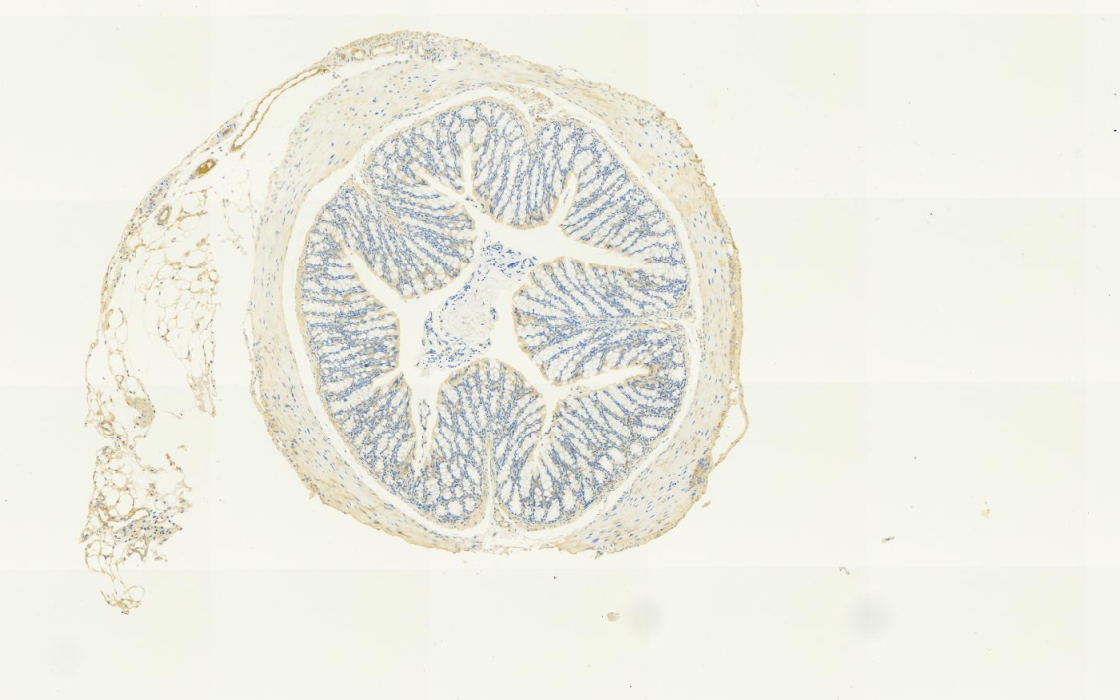

Supplement: Supplementary file 6 [file DataSheet6.zip › Figure-6/fig6-E(IHC Original image)/control-ZO-1_viewcapture.png]

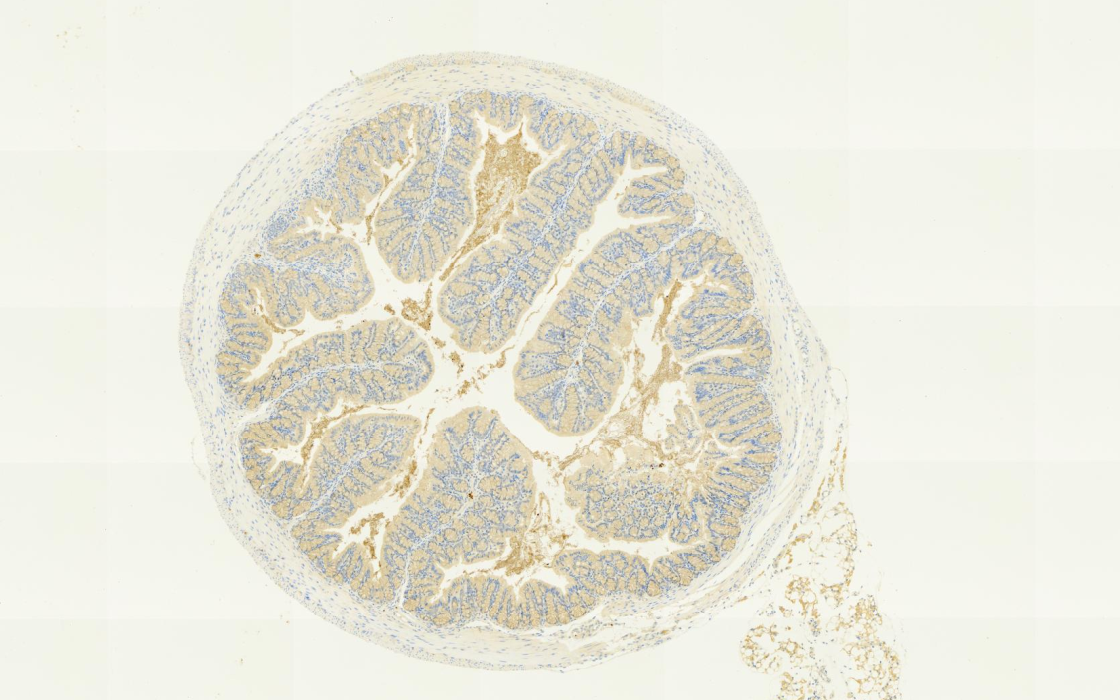

Supplement: Supplementary file 6 [file DataSheet6.zip › Figure-6/fig6-E(IHC Original image)/DGD-D-MUC2_viewcapture.png]

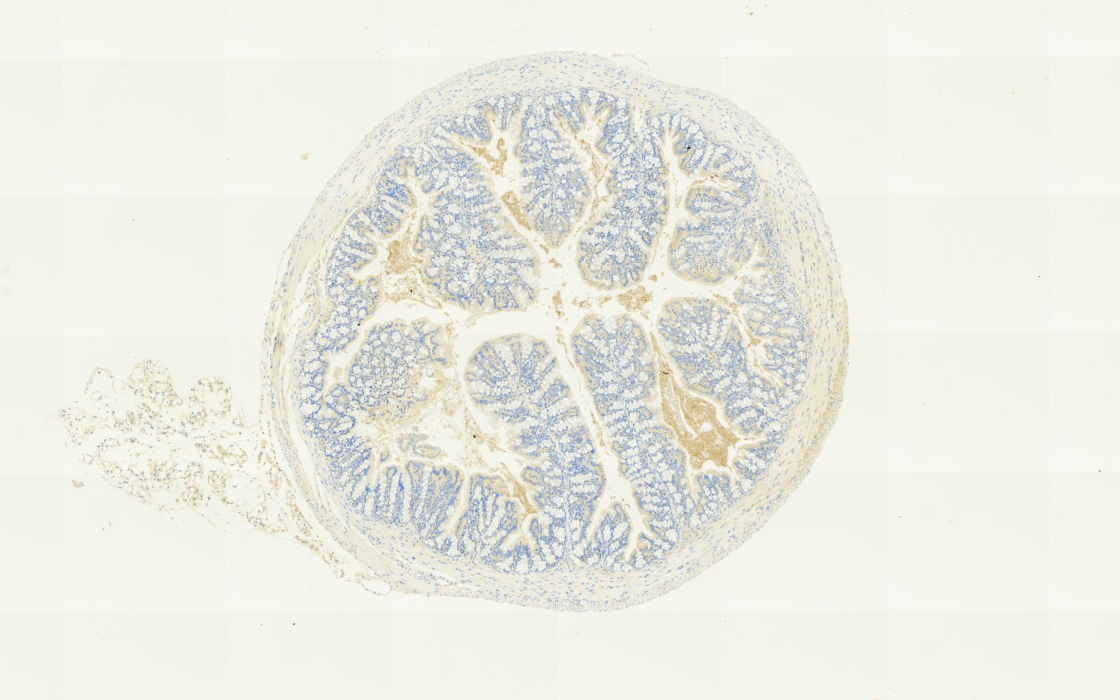

Supplement: Supplementary file 6 [file DataSheet6.zip › Figure-6/fig6-E(IHC Original image)/DGD-D-occludin_viewcapture.png]

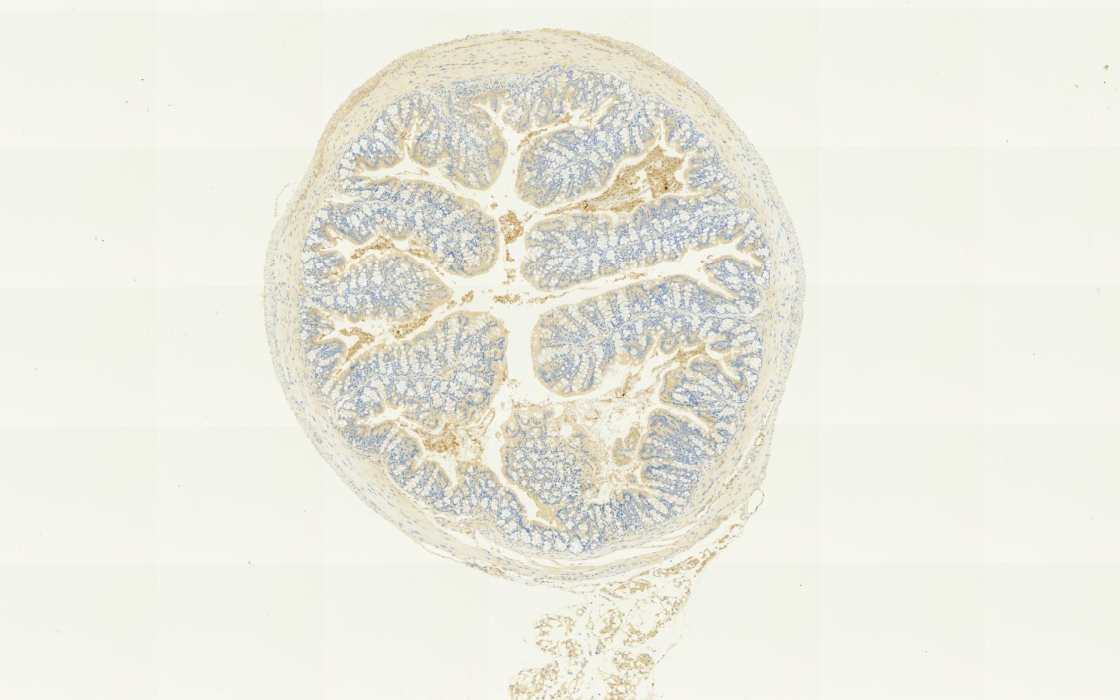

Supplement: Supplementary file 6 [file DataSheet6.zip › Figure-6/fig6-E(IHC Original image)/DGD-D-ZO-1_viewcapture.png]

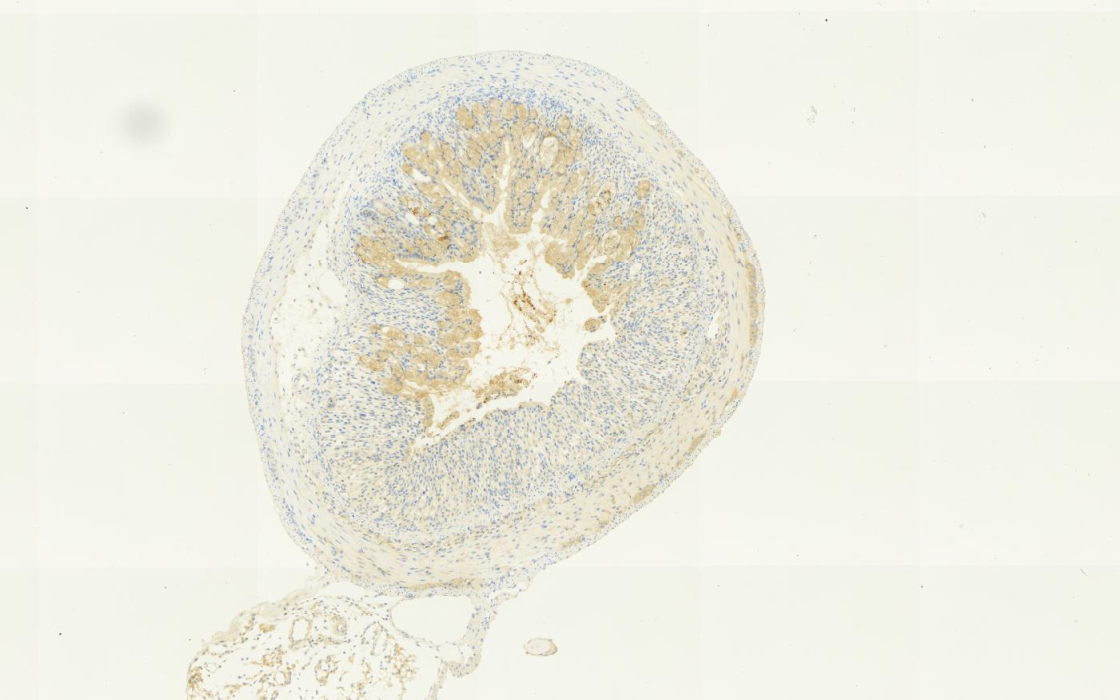

Supplement: Supplementary file 6 [file DataSheet6.zip › Figure-6/fig6-E(IHC Original image)/DSS-MUC2_viewcapture.png]

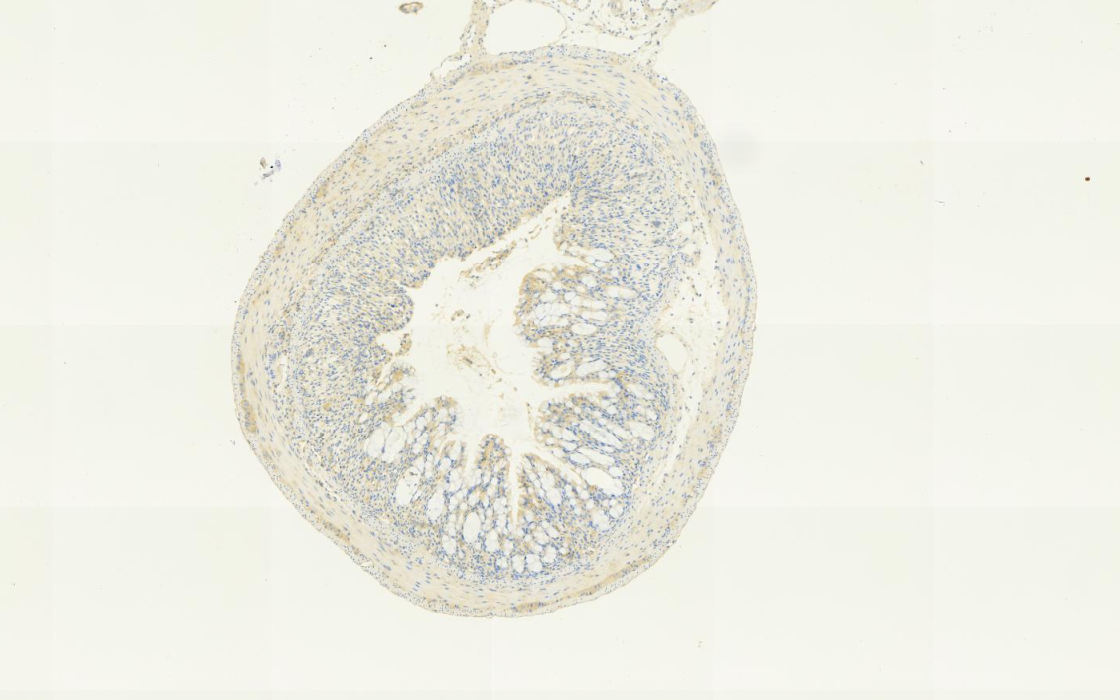

Supplement: Supplementary file 6 [file DataSheet6.zip › Figure-6/fig6-E(IHC Original image)/DSS-occludin_viewcapture.png]

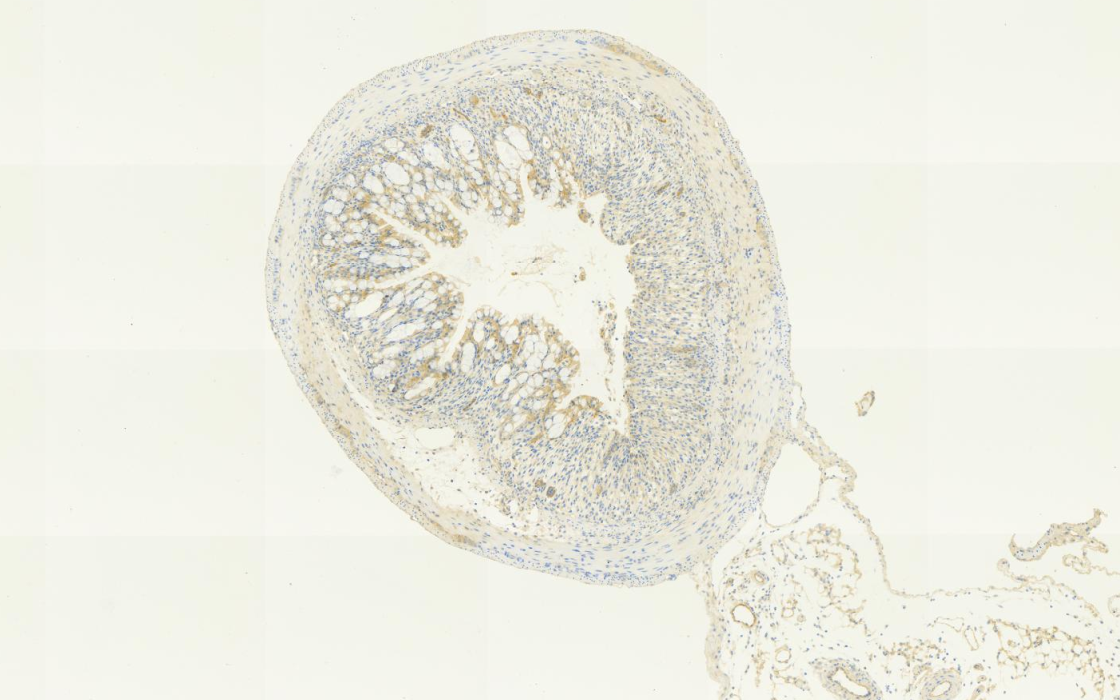

Supplement: Supplementary file 6 [file DataSheet6.zip › Figure-6/fig6-E(IHC Original image)/DSS-ZO1_viewcapture.png]

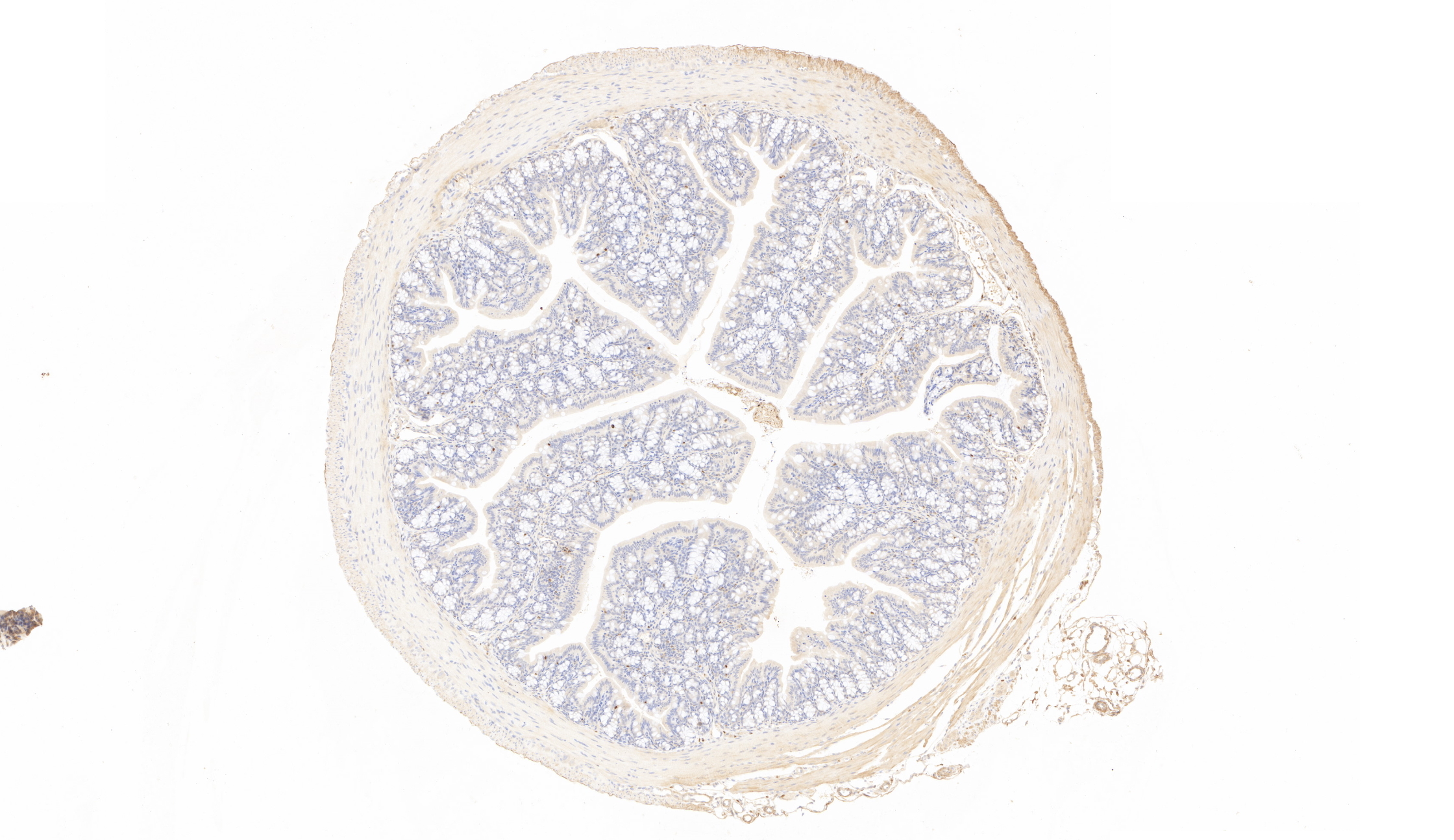

Supplement: Supplementary file 9 [file DataSheet9.zip › Figure-9/fig9-A(IHC Original image)/control-5-LOX.jpg]

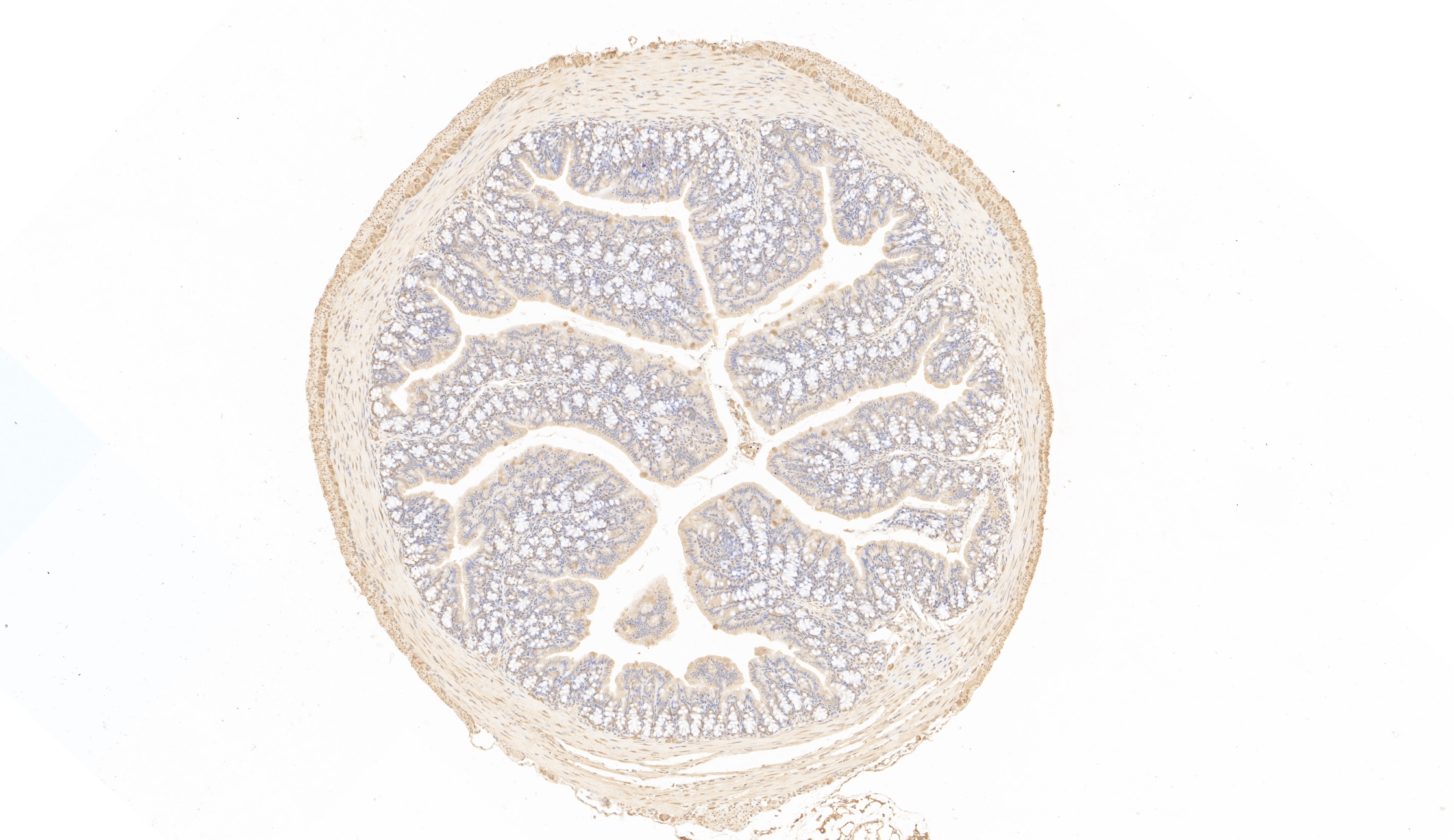

Supplement: Supplementary file 9 [file DataSheet9.zip › Figure-9/fig9-A(IHC Original image)/control-MYD88.jpg]

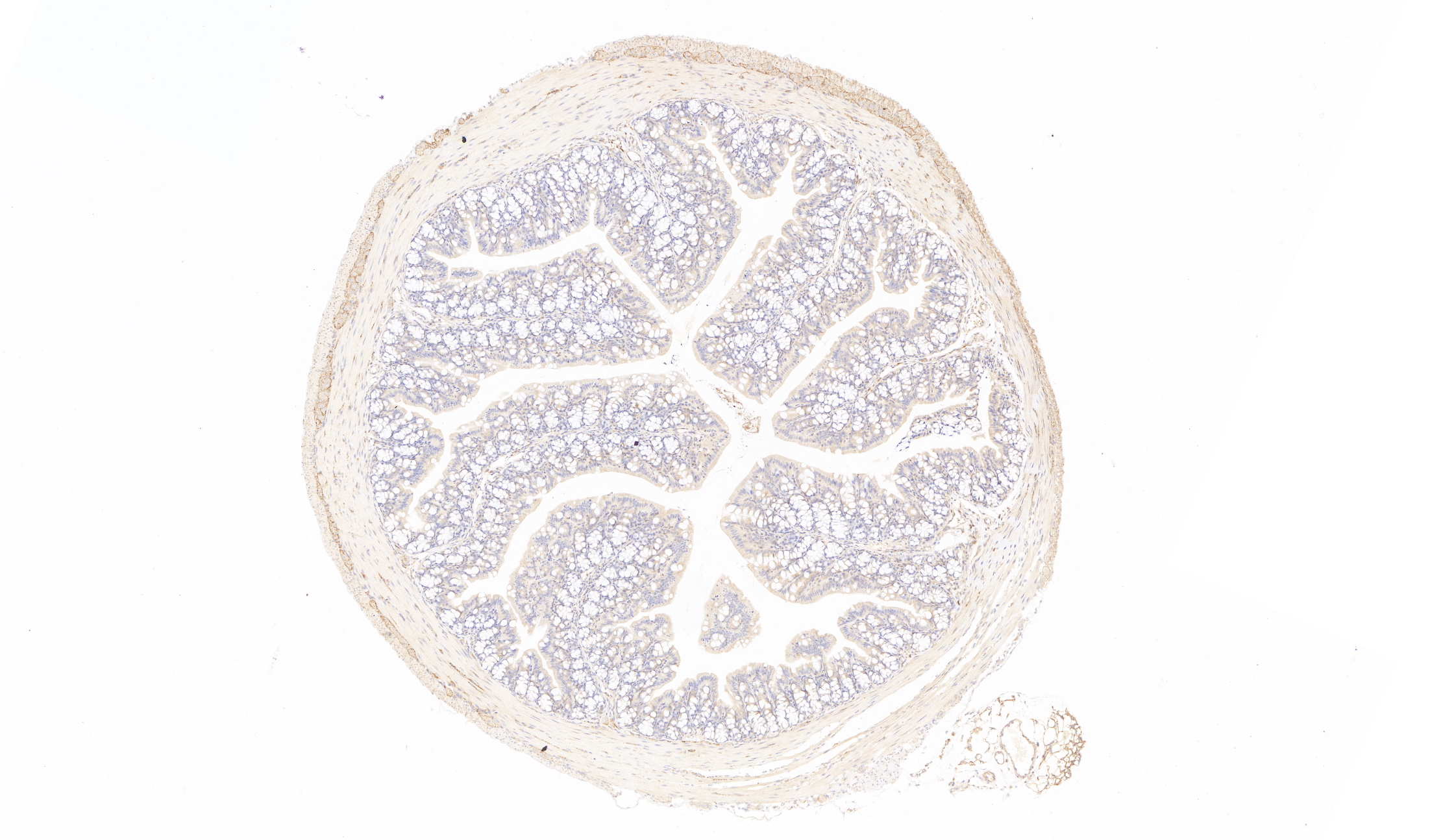

Supplement: Supplementary file 9 [file DataSheet9.zip › Figure-9/fig9-A(IHC Original image)/control-NF-KB p65.jpg]

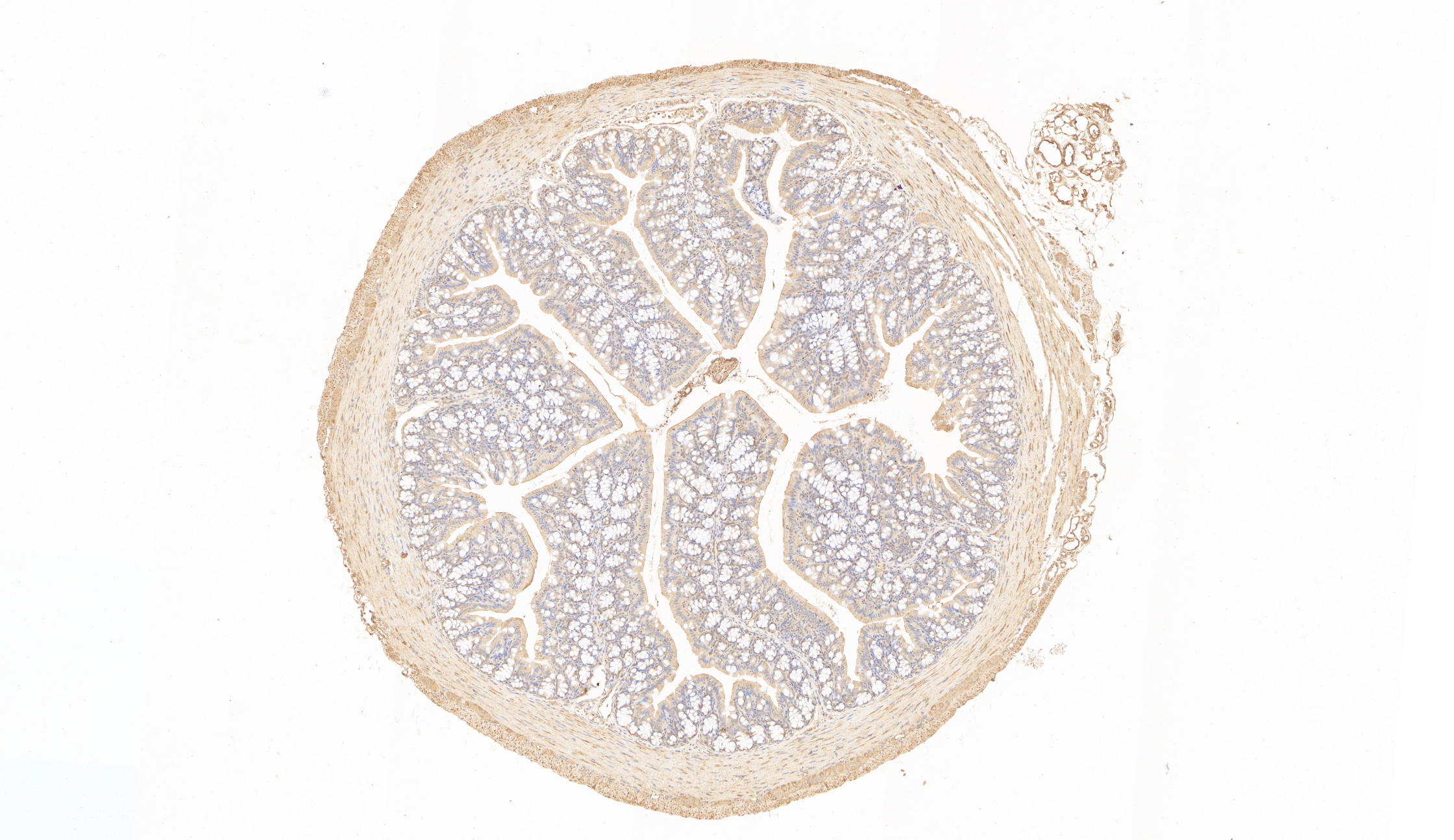

Supplement: Supplementary file 9 [file DataSheet9.zip › Figure-9/fig9-A(IHC Original image)/control-NLRP3.jpg]

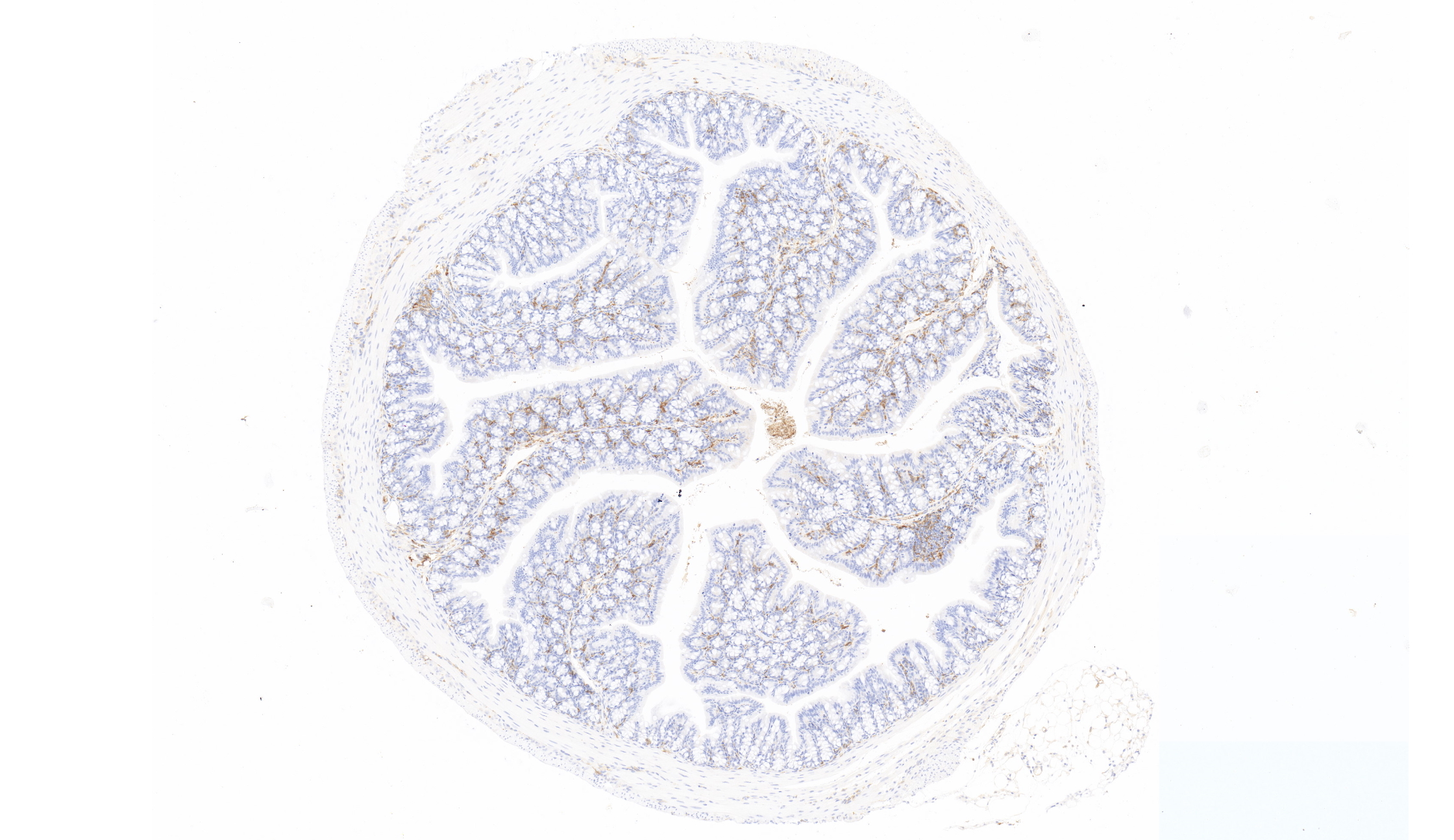

Supplement: Supplementary file 9 [file DataSheet9.zip › Figure-9/fig9-A(IHC Original image)/control-TLR4.jpg]

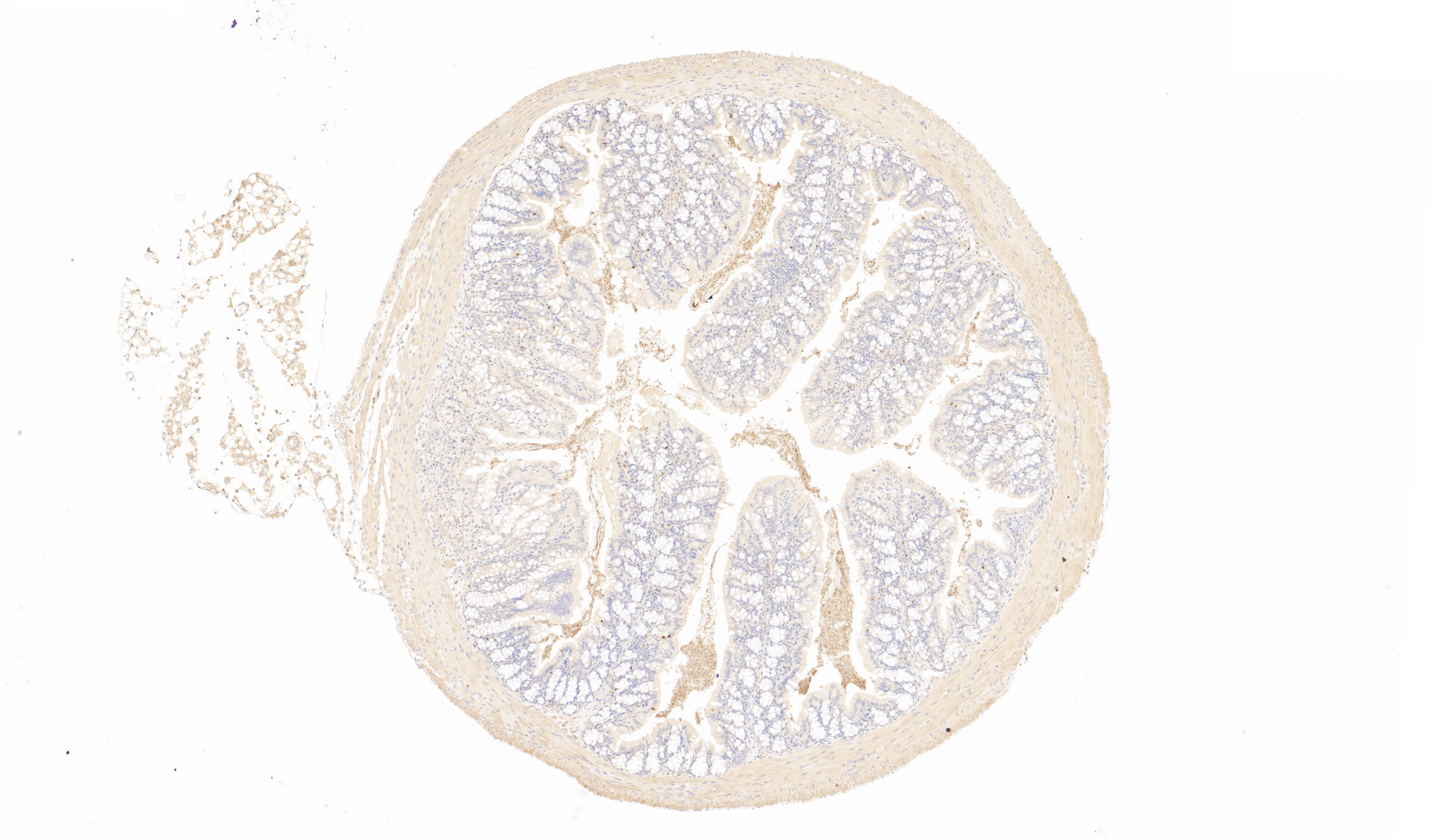

Supplement: Supplementary file 9 [file DataSheet9.zip › Figure-9/fig9-A(IHC Original image)/DGD-D-5-LOX.jpg]

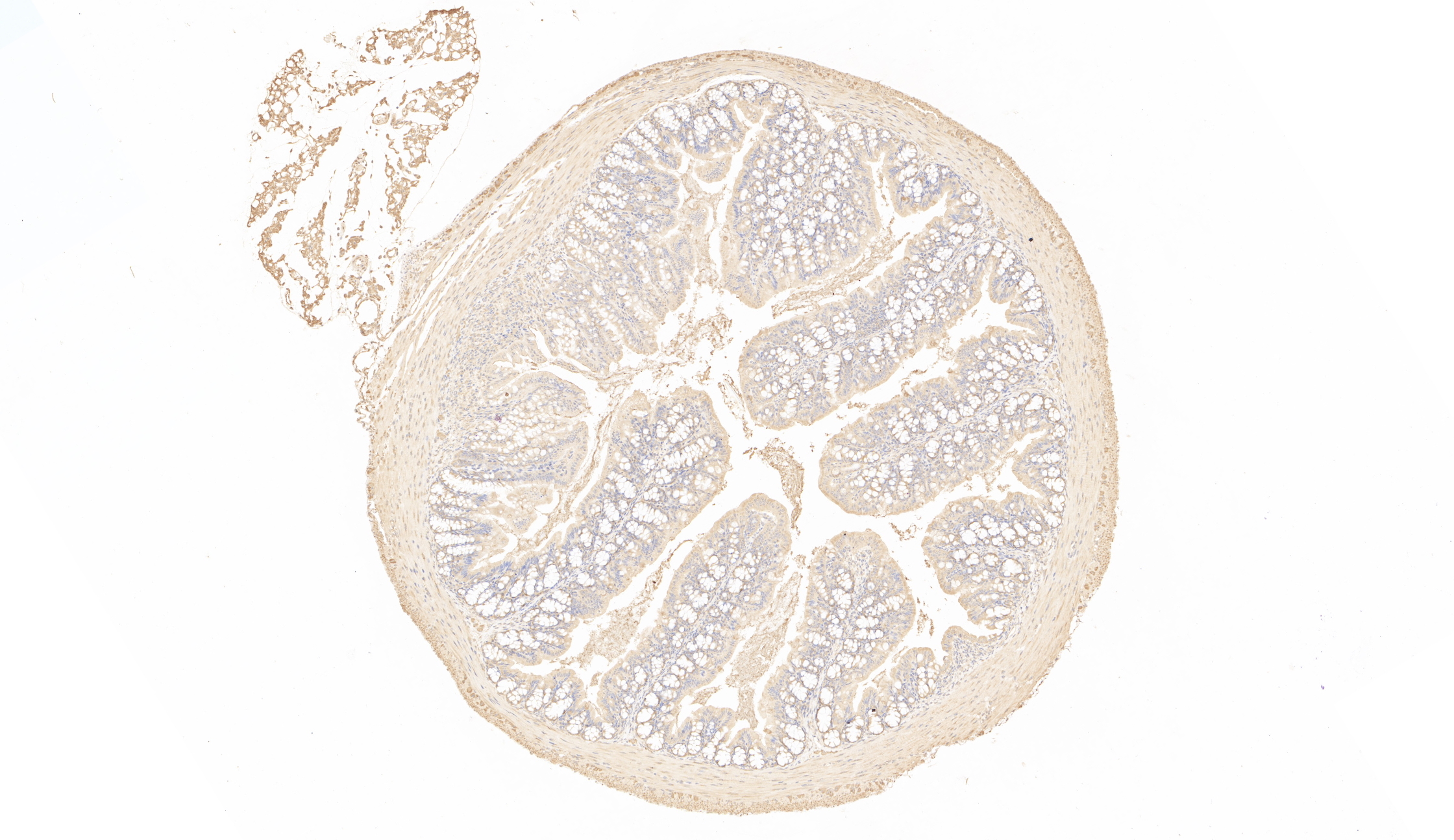

Supplement: Supplementary file 9 [file DataSheet9.zip › Figure-9/fig9-A(IHC Original image)/DGD-D-MYD88.jpg]

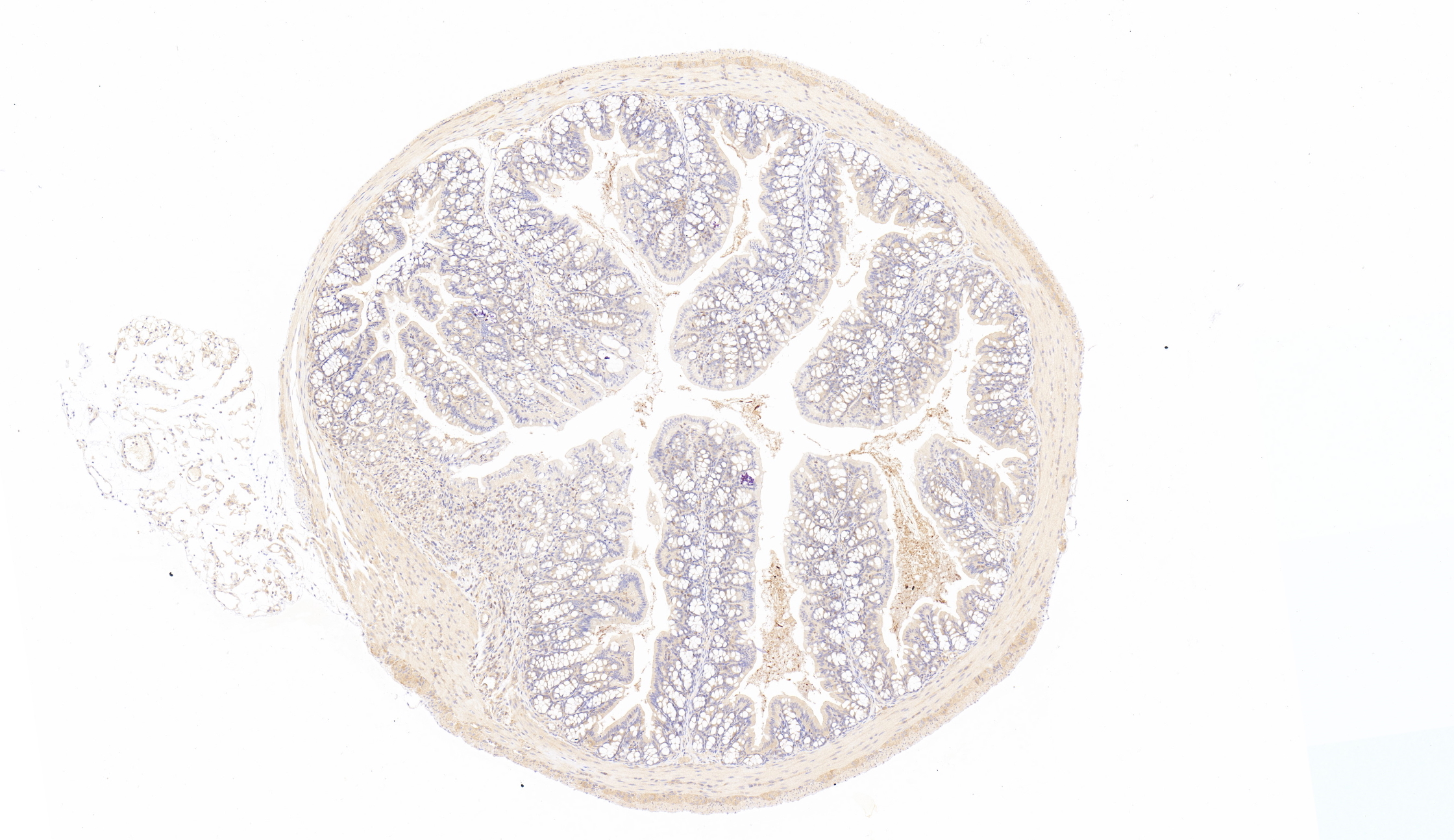

Supplement: Supplementary file 9 [file DataSheet9.zip › Figure-9/fig9-A(IHC Original image)/DGD-D-NF-KB-P65.jpg]

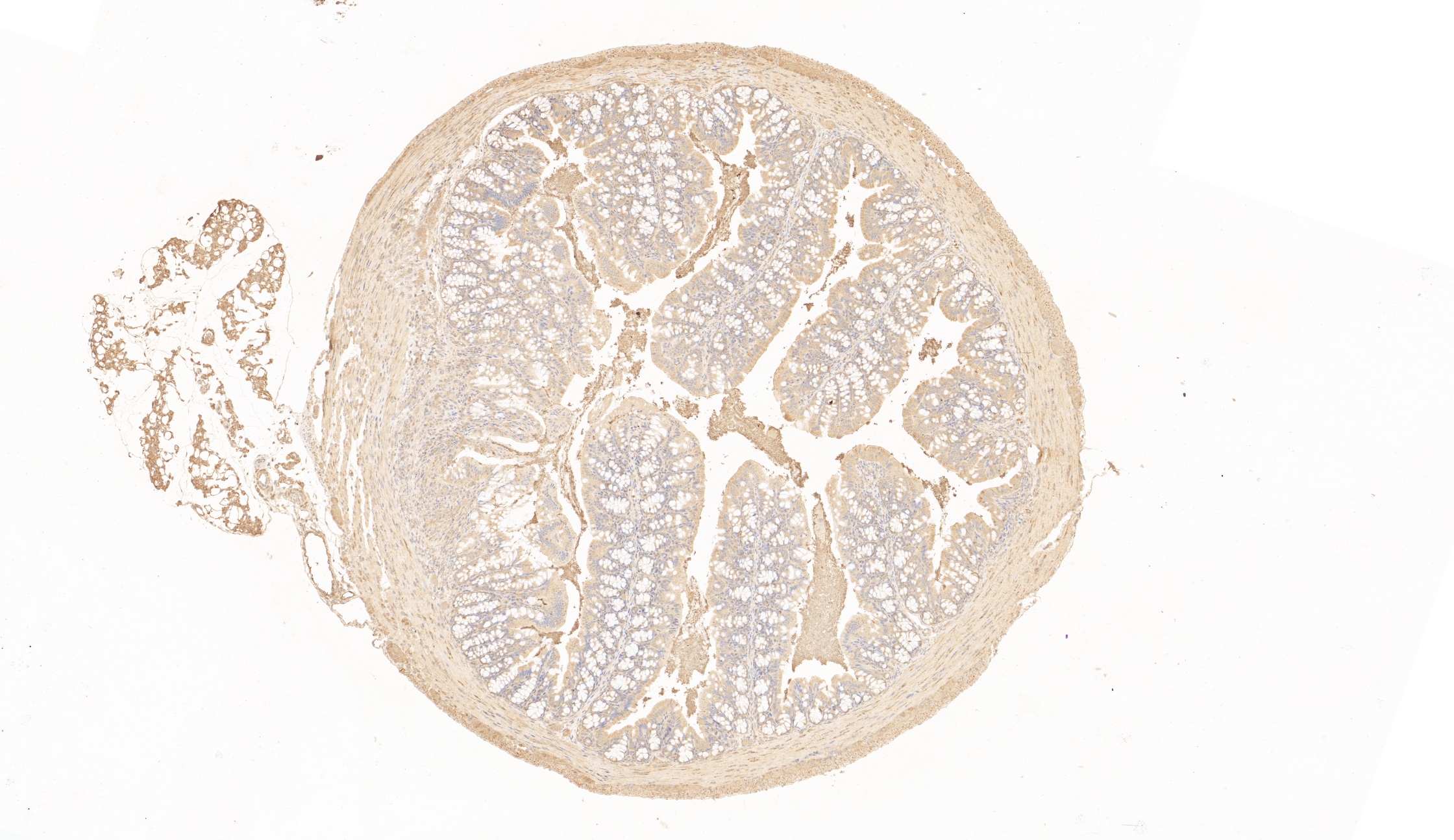

Supplement: Supplementary file 9 [file DataSheet9.zip › Figure-9/fig9-A(IHC Original image)/DGD-D-NLRP3.jpg]

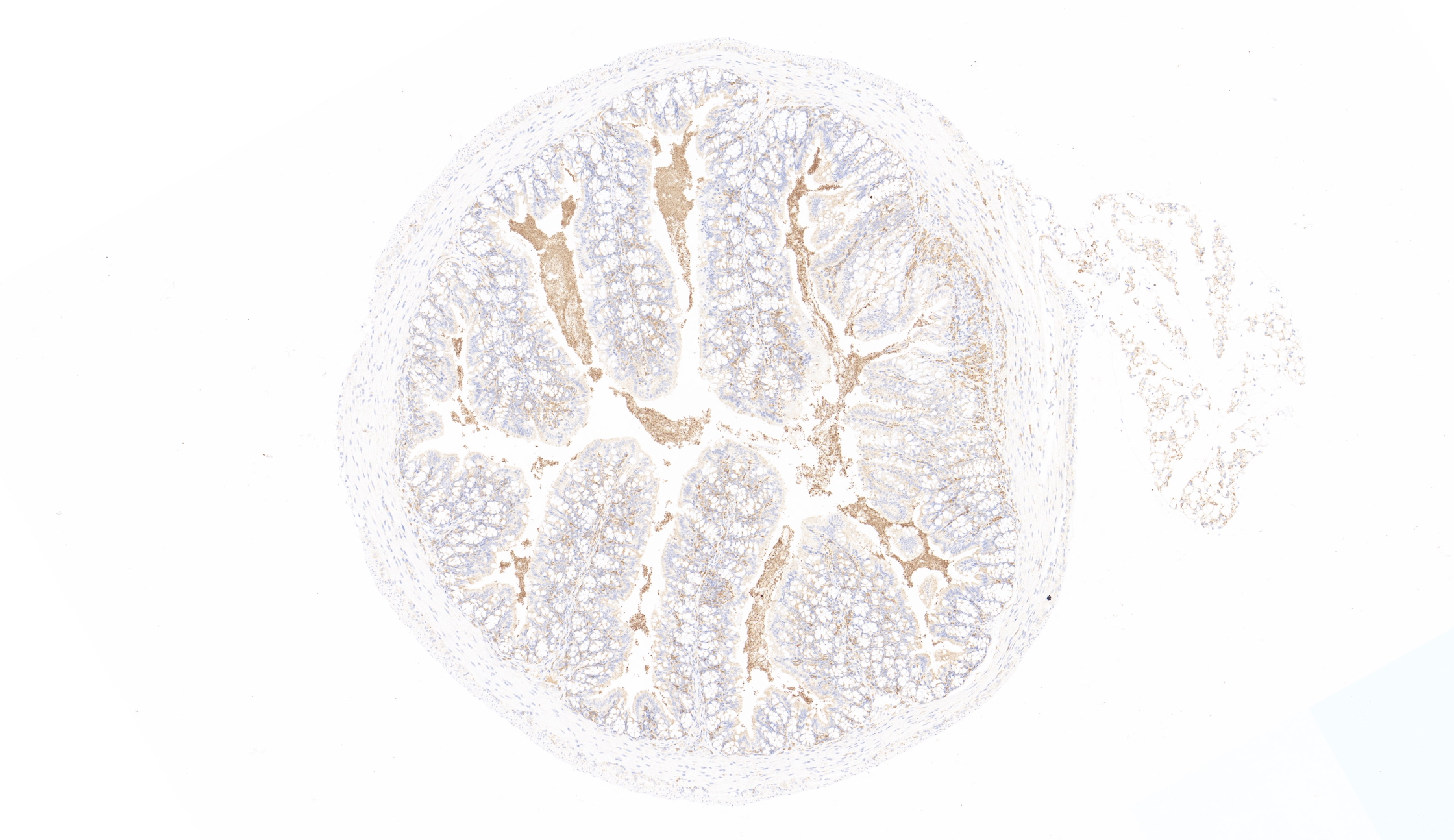

Supplement: Supplementary file 9 [file DataSheet9.zip › Figure-9/fig9-A(IHC Original image)/DGD-D-TLR4.jpg]

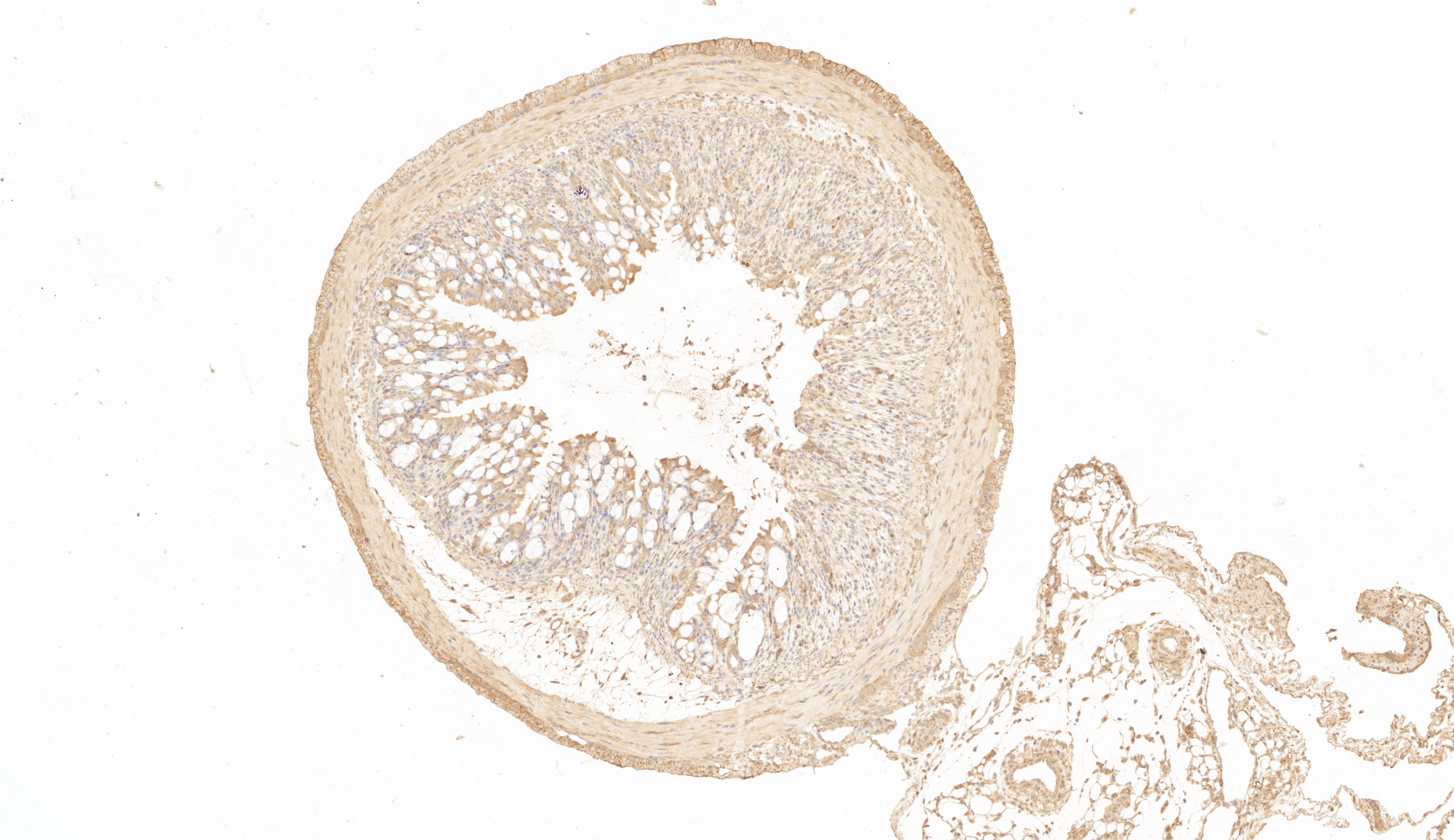

Supplement: Supplementary file 9 [file DataSheet9.zip › Figure-9/fig9-A(IHC Original image)/DSS - NLRP3.jpg]

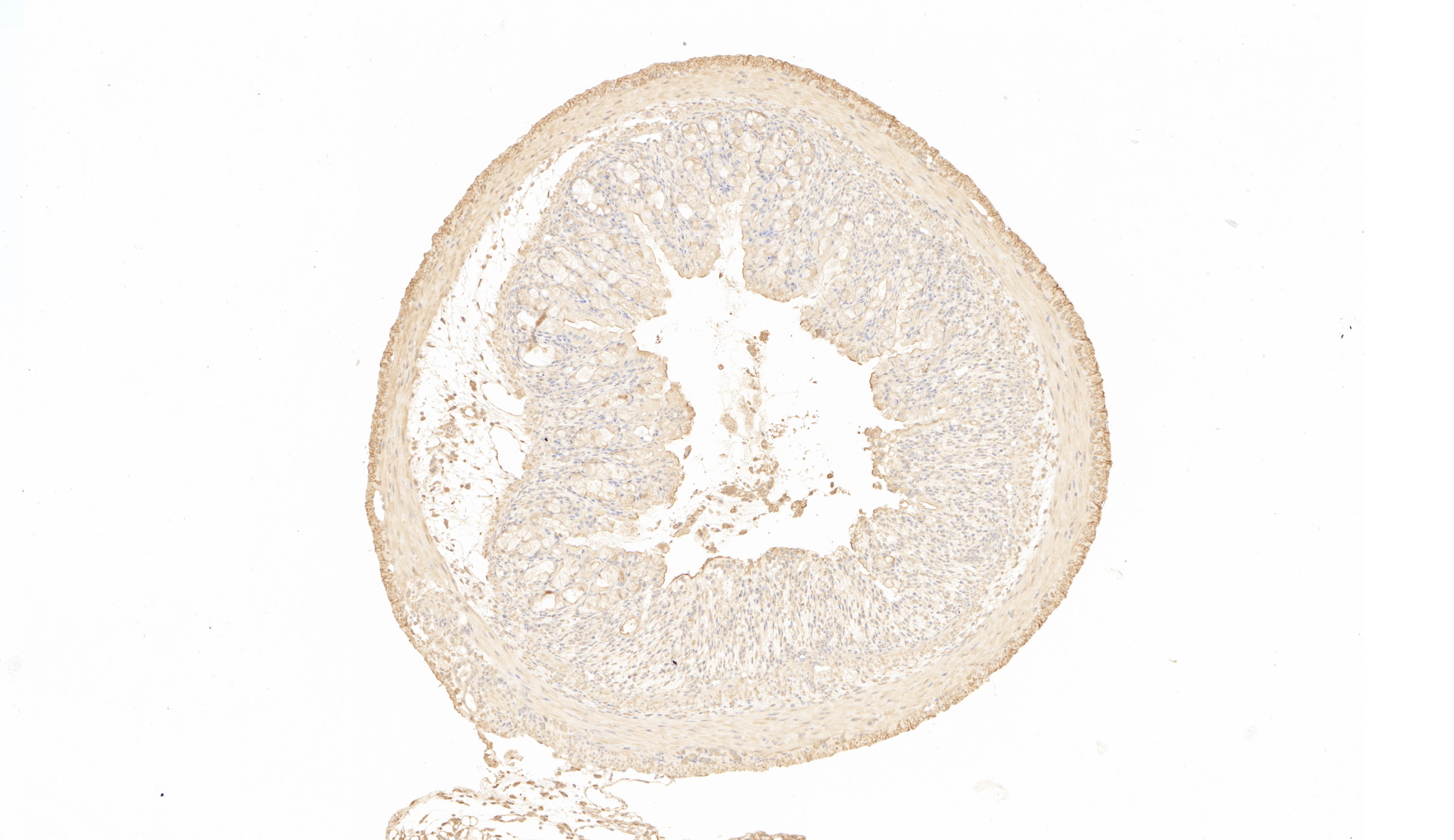

Supplement: Supplementary file 9 [file DataSheet9.zip › Figure-9/fig9-A(IHC Original image)/DSS- MYD88.jpg]

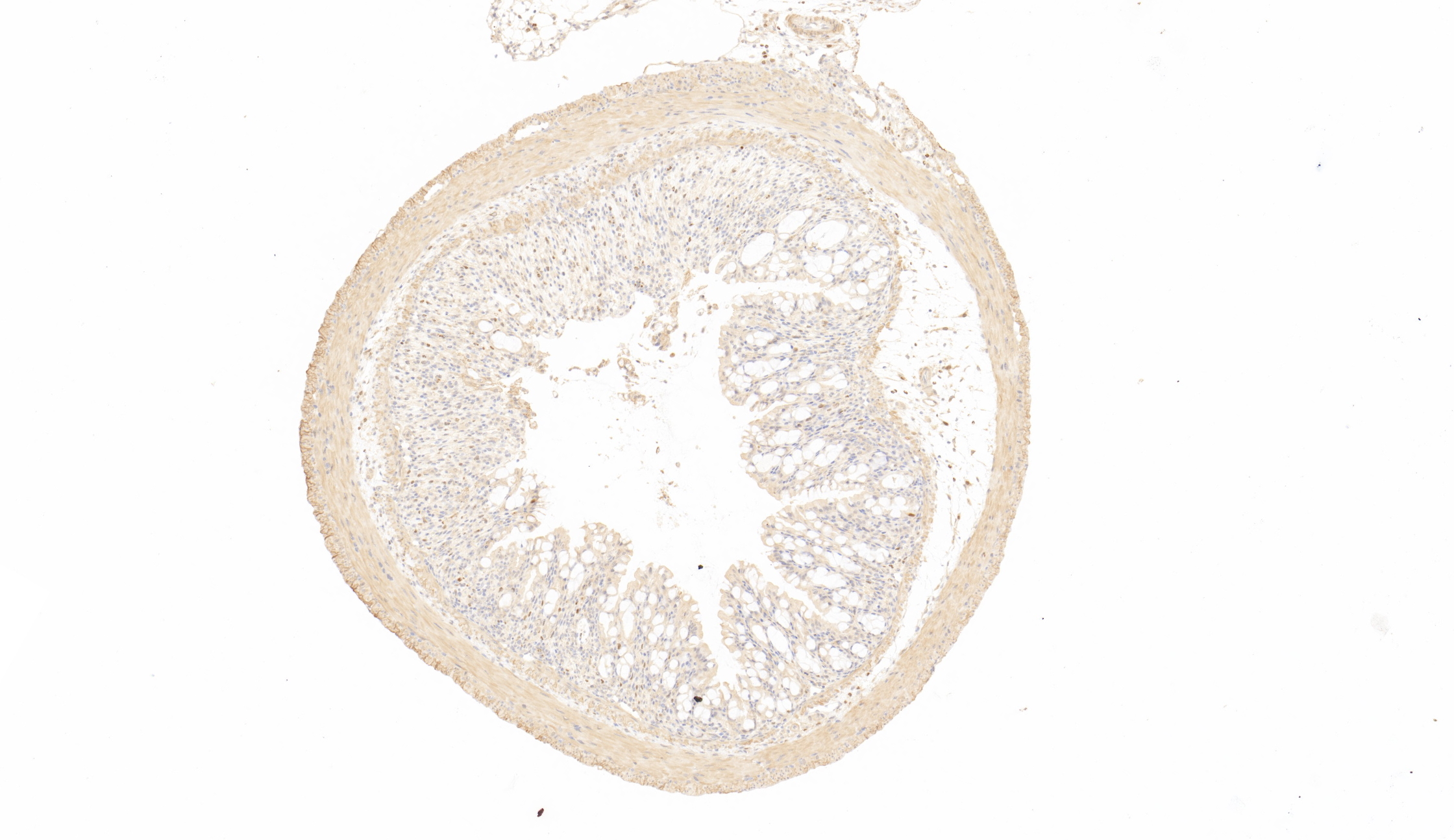

Supplement: Supplementary file 9 [file DataSheet9.zip › Figure-9/fig9-A(IHC Original image)/DSS-5-LOX.jpg]

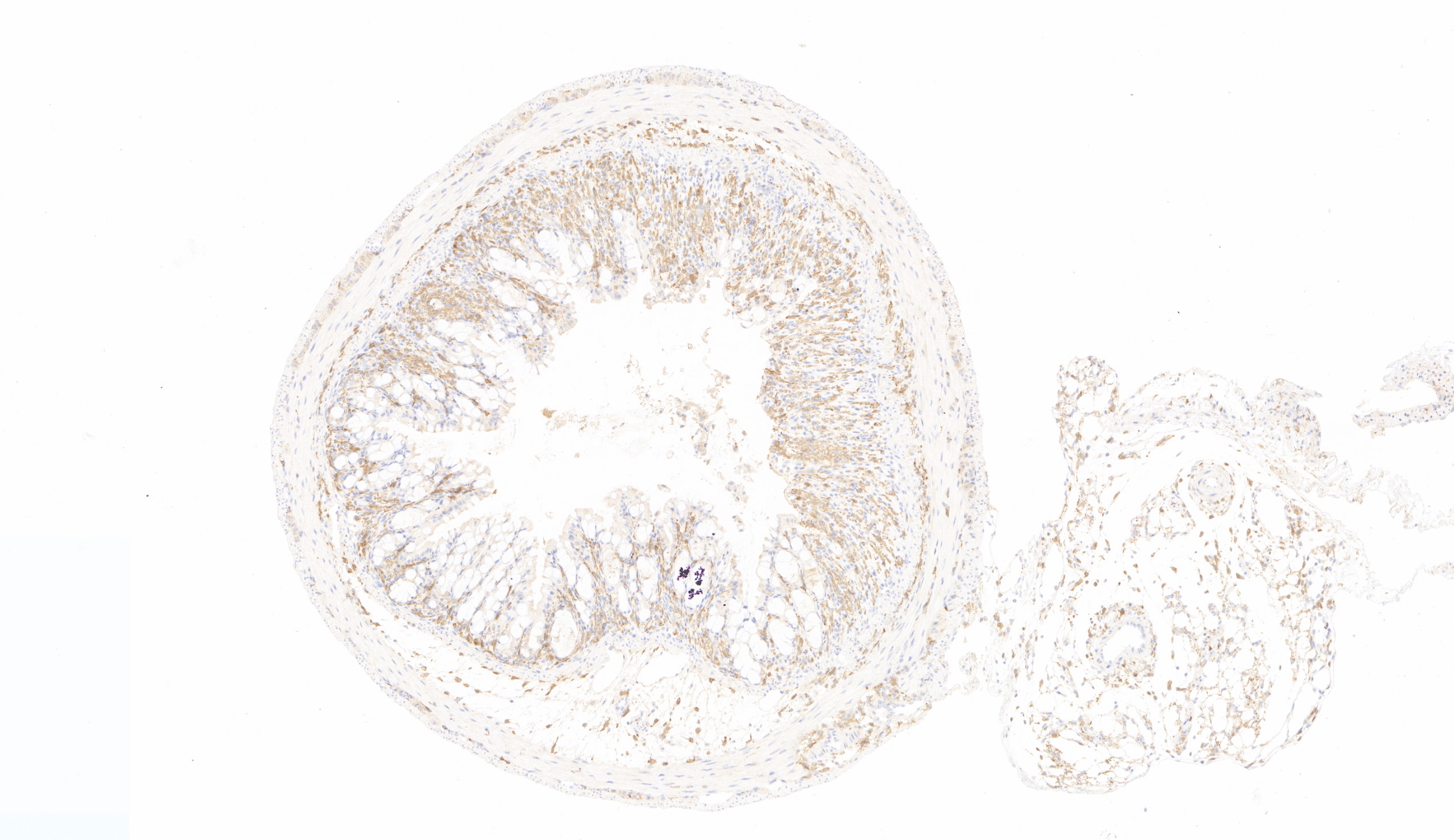

Supplement: Supplementary file 9 [file DataSheet9.zip › Figure-9/fig9-A(IHC Original image)/DSS-TLR4.jpg]

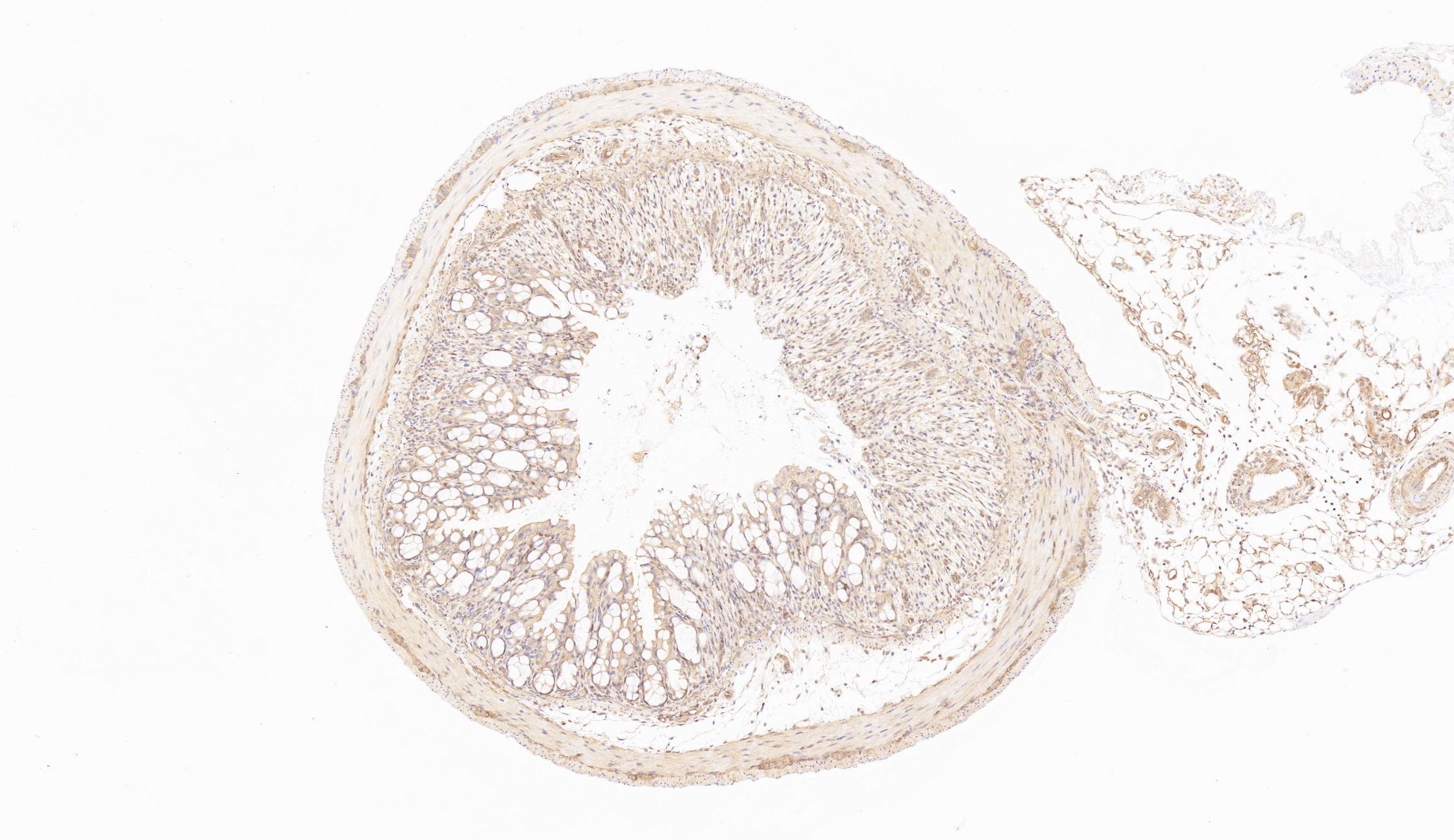

Supplement: Supplementary file 9 [file DataSheet9.zip › Figure-9/fig9-A(IHC Original image)/DSSNF-KB-P65.jpg]

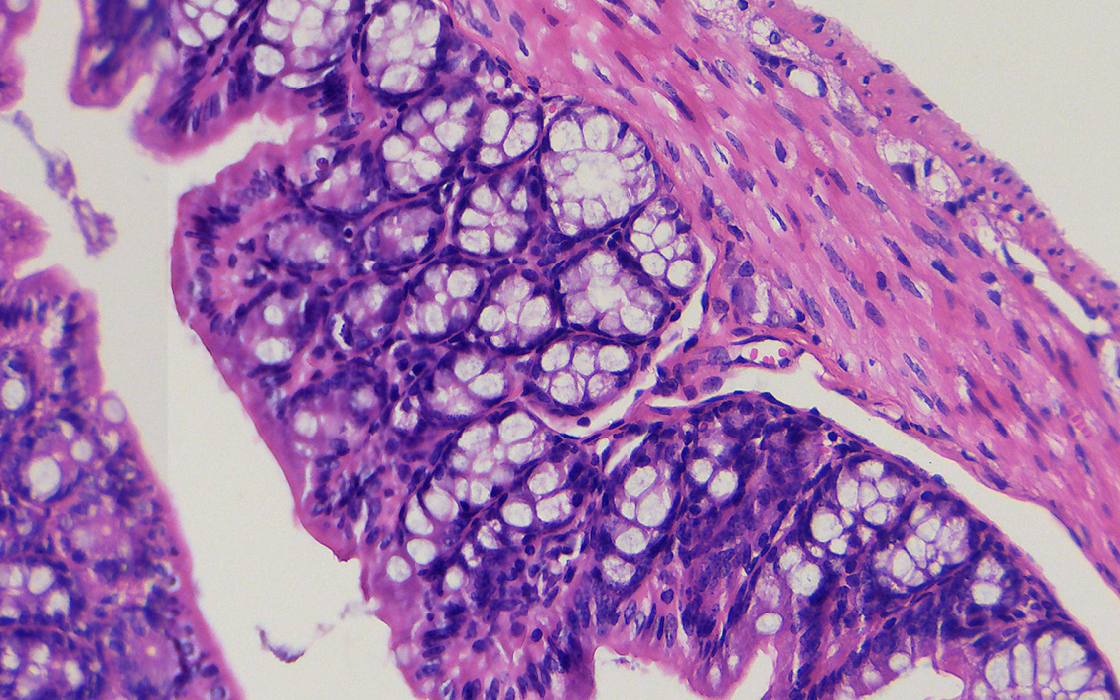

Supplement: Supplementary file 11 [file DataSheet11.zip › fig5-A(HE Original image)/5-ASA(1).png]

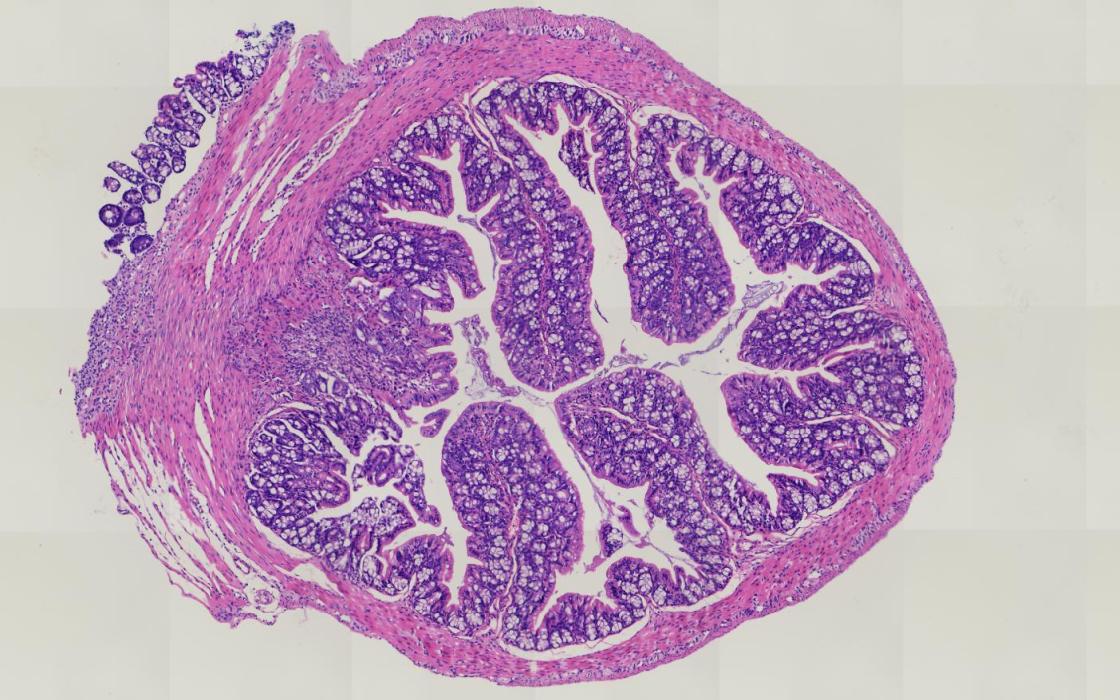

Supplement: Supplementary file 11 [file DataSheet11.zip › fig5-A(HE Original image)/5-ASA.png]

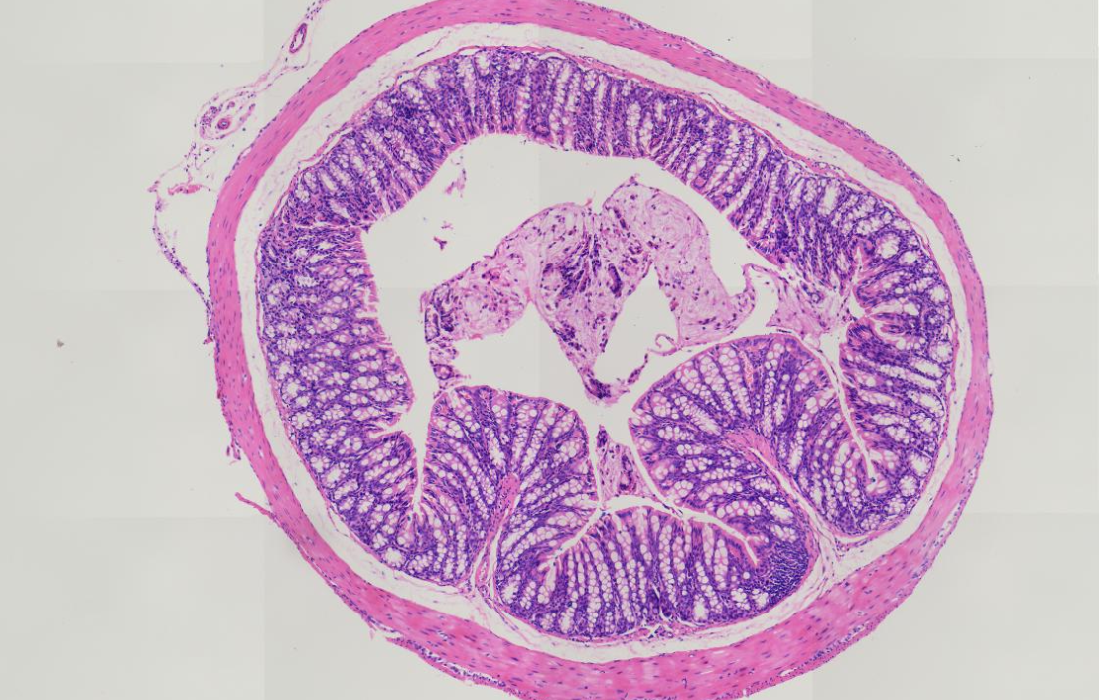

Supplement: Supplementary file 11 [file DataSheet11.zip › fig5-A(HE Original image)/control.png]

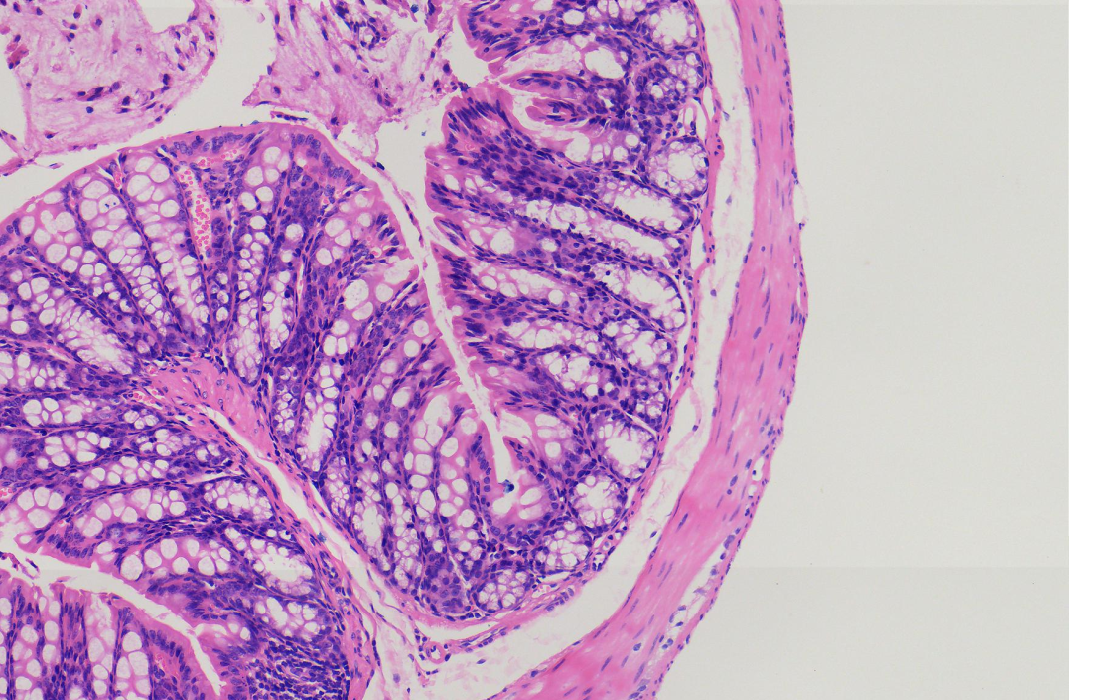

Supplement: Supplementary file 11 [file DataSheet11.zip › fig5-A(HE Original image)/control(1).png]

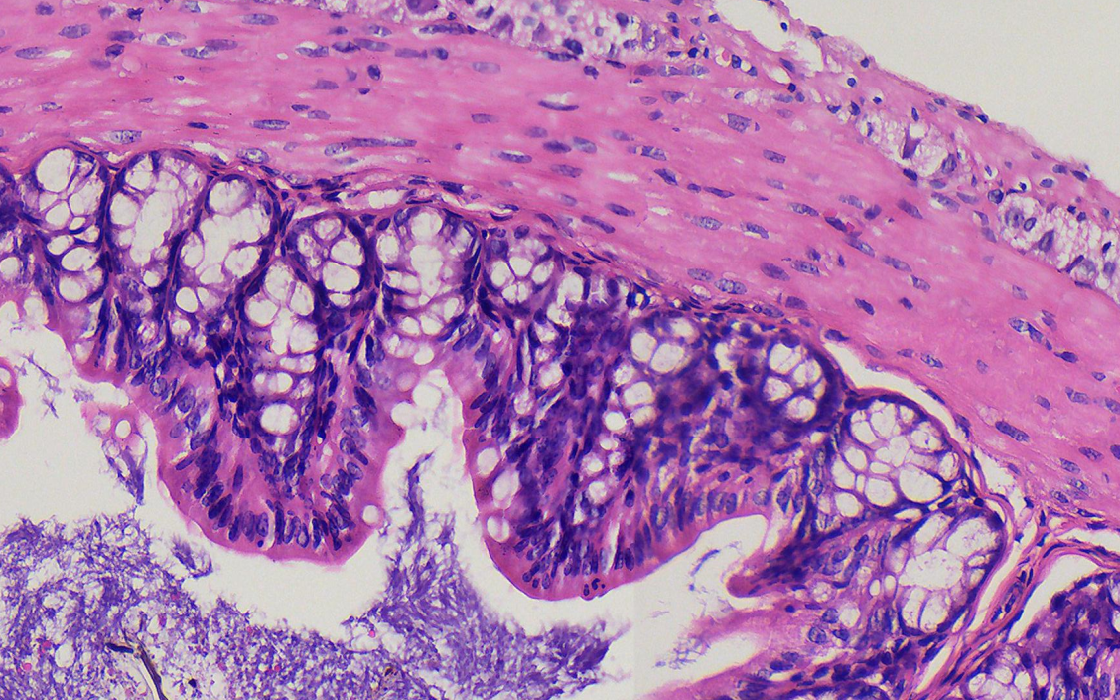

Supplement: Supplementary file 11 [file DataSheet11.zip › fig5-A(HE Original image)/DGD-D(1).png]

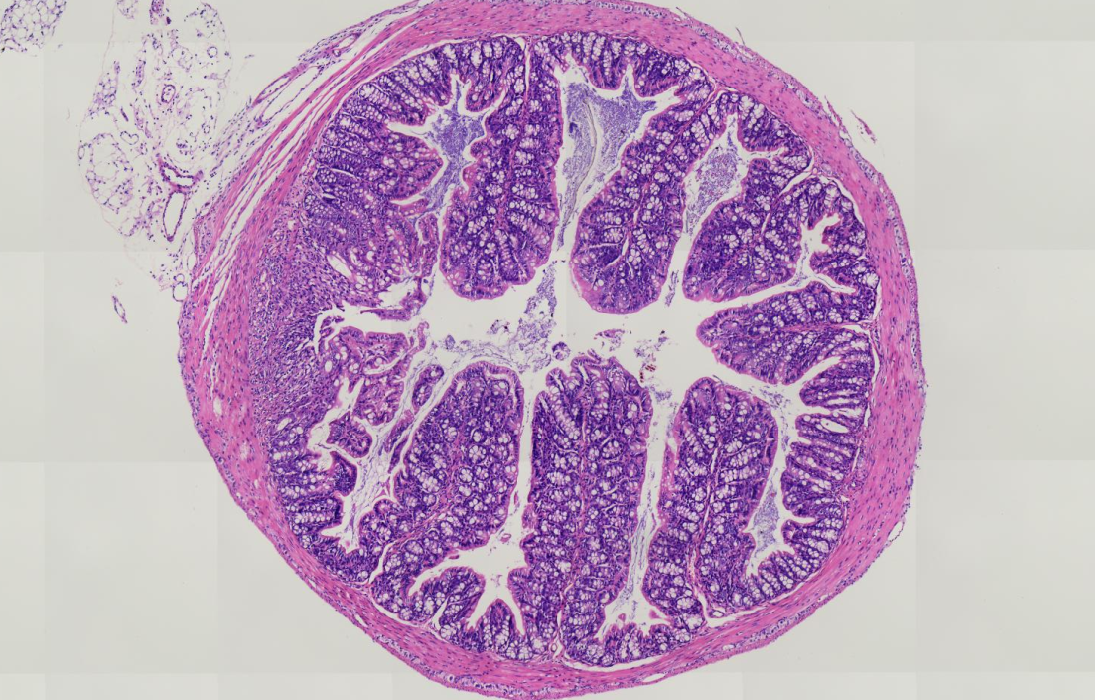

Supplement: Supplementary file 11 [file DataSheet11.zip › fig5-A(HE Original image)/DGD-D.png]

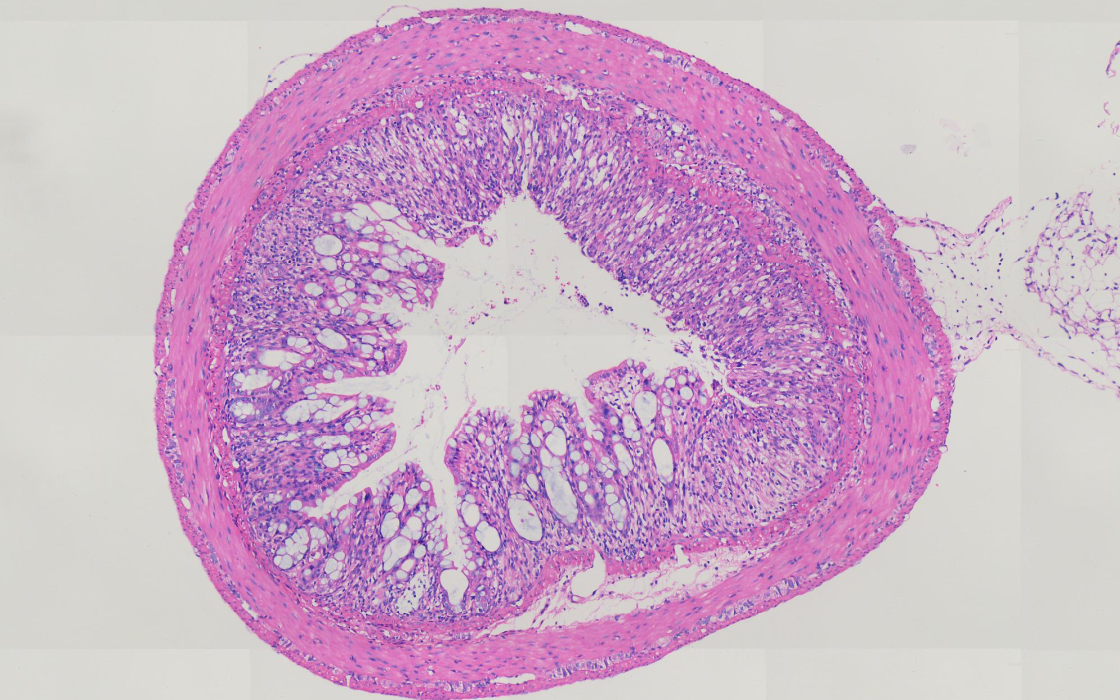

Supplement: Supplementary file 11 [file DataSheet11.zip › fig5-A(HE Original image)/DSS.png]

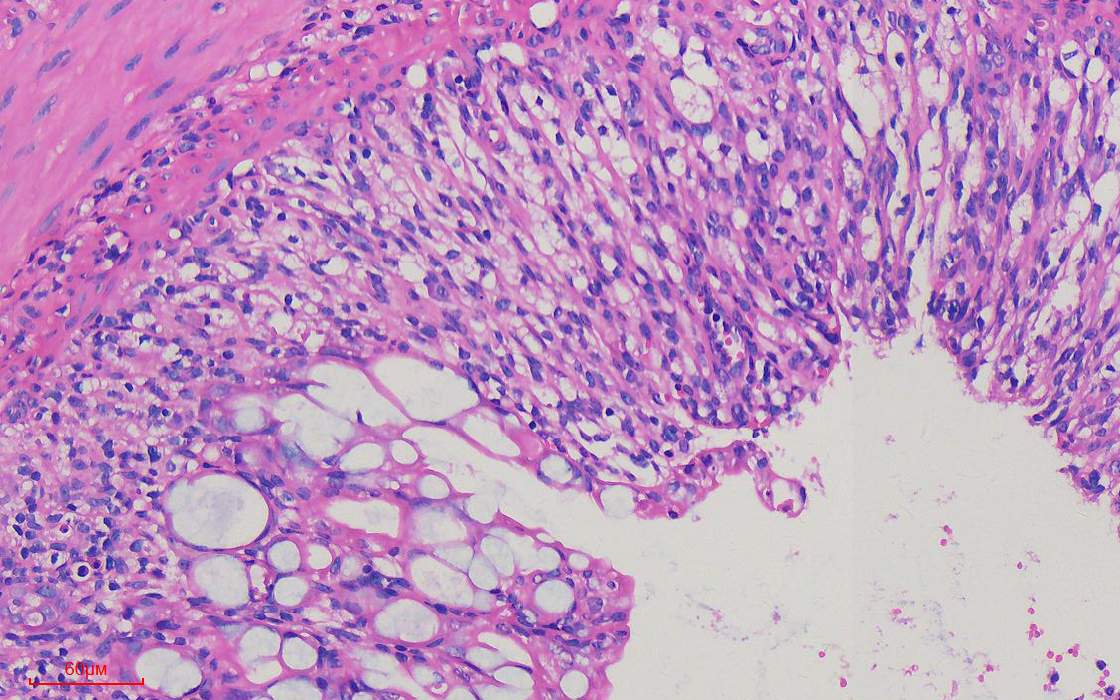

Supplement: Supplementary file 11 [file DataSheet11.zip › fig5-A(HE Original image)/DSS(1).png]

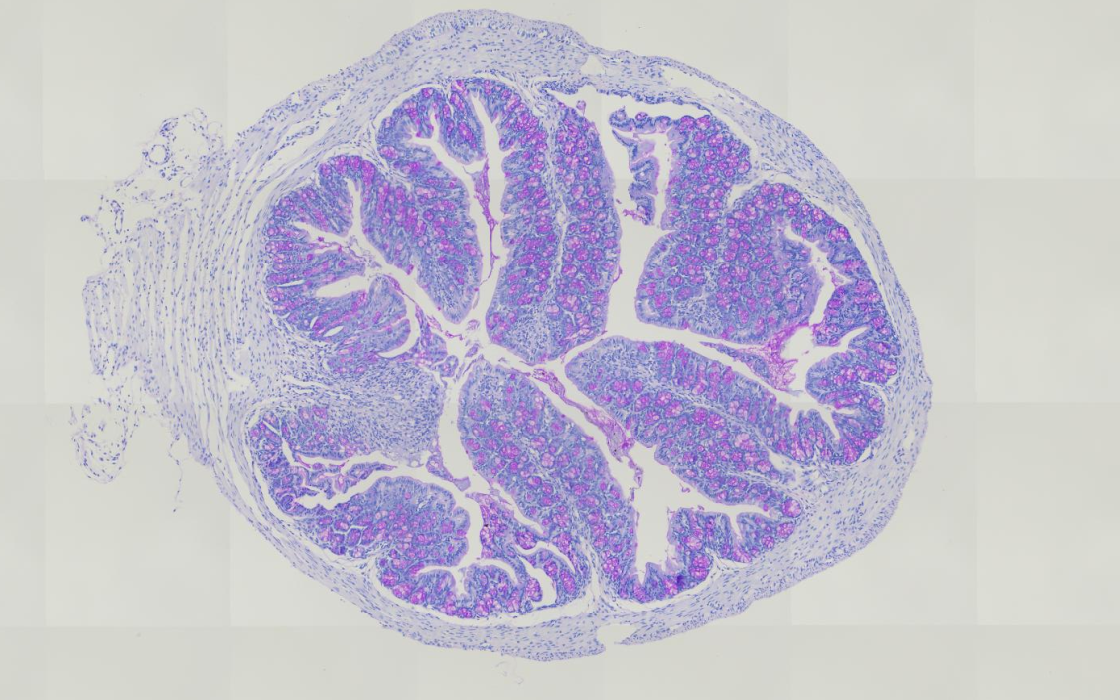

Supplement: Supplementary file 12 [file DataSheet12.zip › fig5-C(PAS Original image)/5-ASA.png]

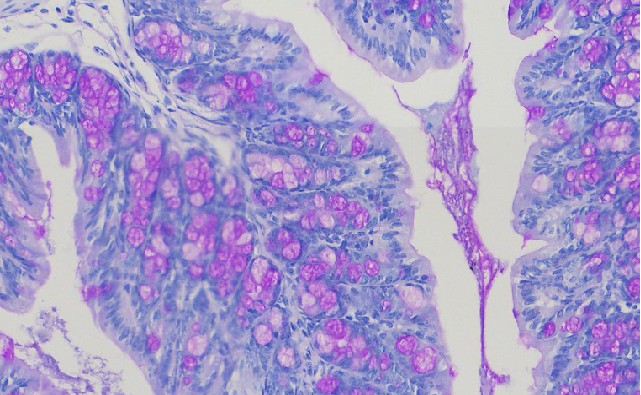

Supplement: Supplementary file 12 [file DataSheet12.zip › fig5-C(PAS Original image)/5-ASA(1).jpg]

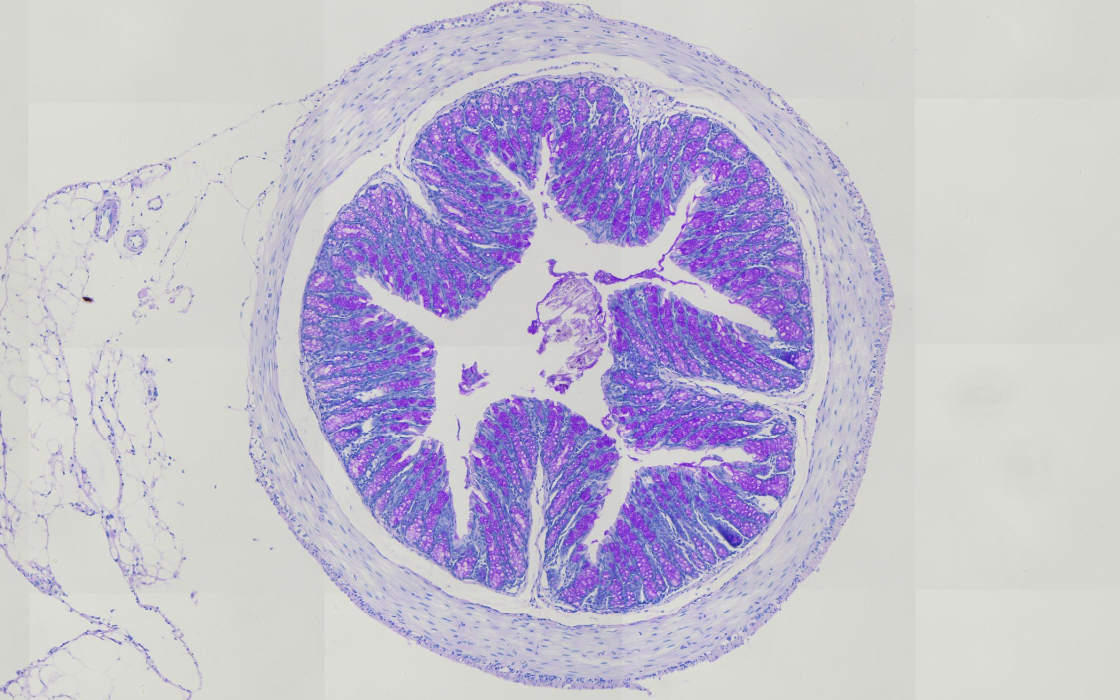

Supplement: Supplementary file 12 [file DataSheet12.zip › fig5-C(PAS Original image)/Control.png]

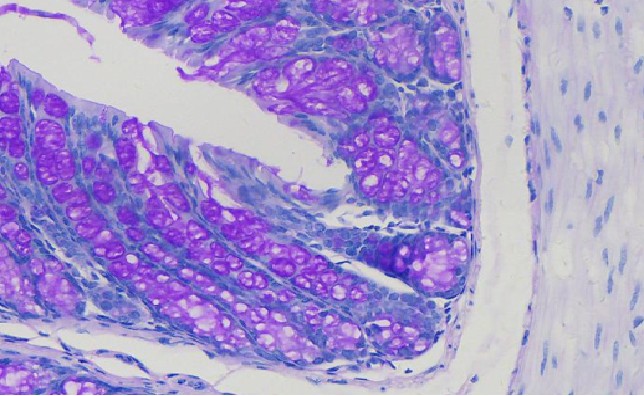

Supplement: Supplementary file 12 [file DataSheet12.zip › fig5-C(PAS Original image)/Control(1).jpg]

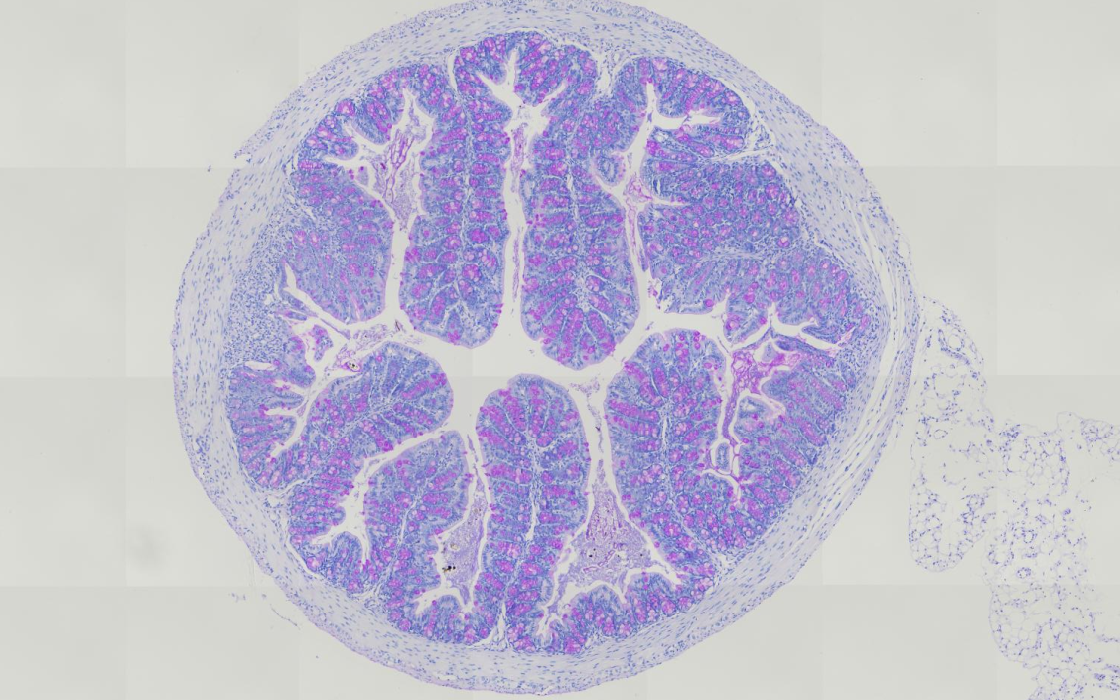

Supplement: Supplementary file 12 [file DataSheet12.zip › fig5-C(PAS Original image)/DGD-D.png]

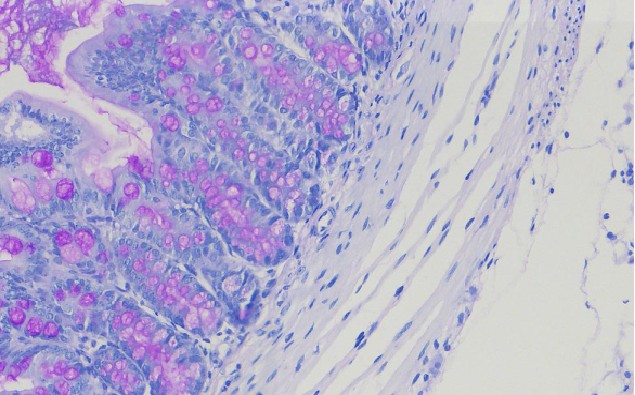

Supplement: Supplementary file 12 [file DataSheet12.zip › fig5-C(PAS Original image)/DGD-D(1).jpg]

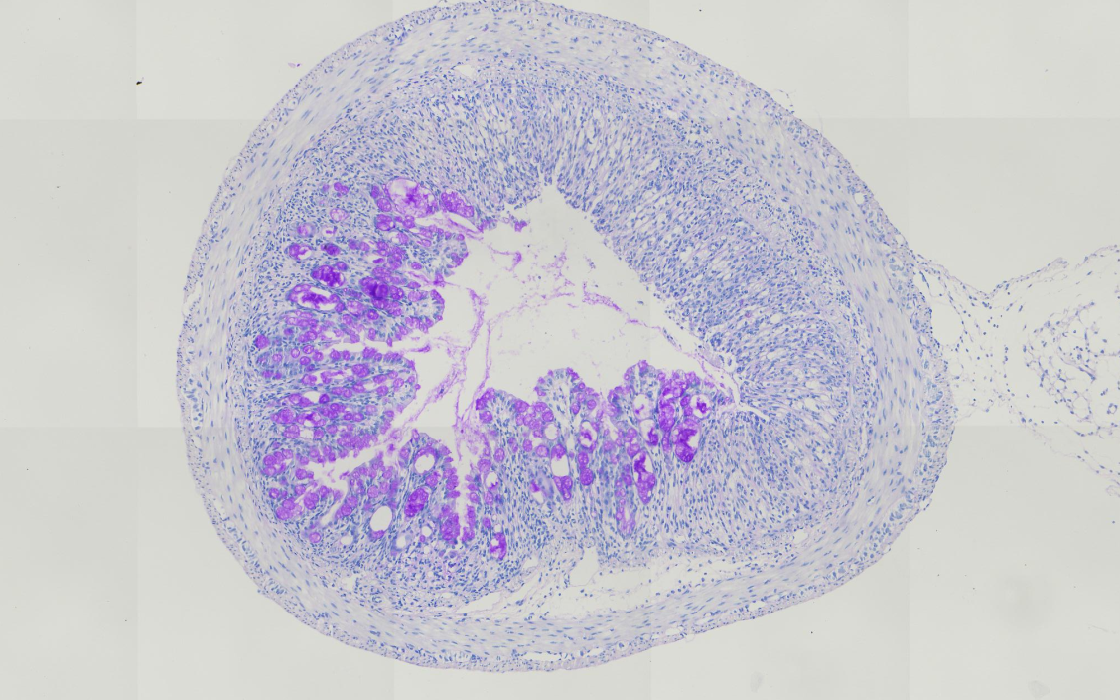

Supplement: Supplementary file 12 [file DataSheet12.zip › fig5-C(PAS Original image)/DSS.png]

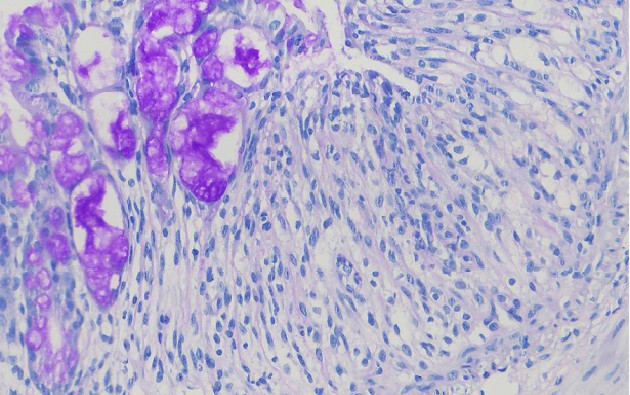

Supplement: Supplementary file 12 [file DataSheet12.zip › fig5-C(PAS Original image)/DSS(1).jpg]

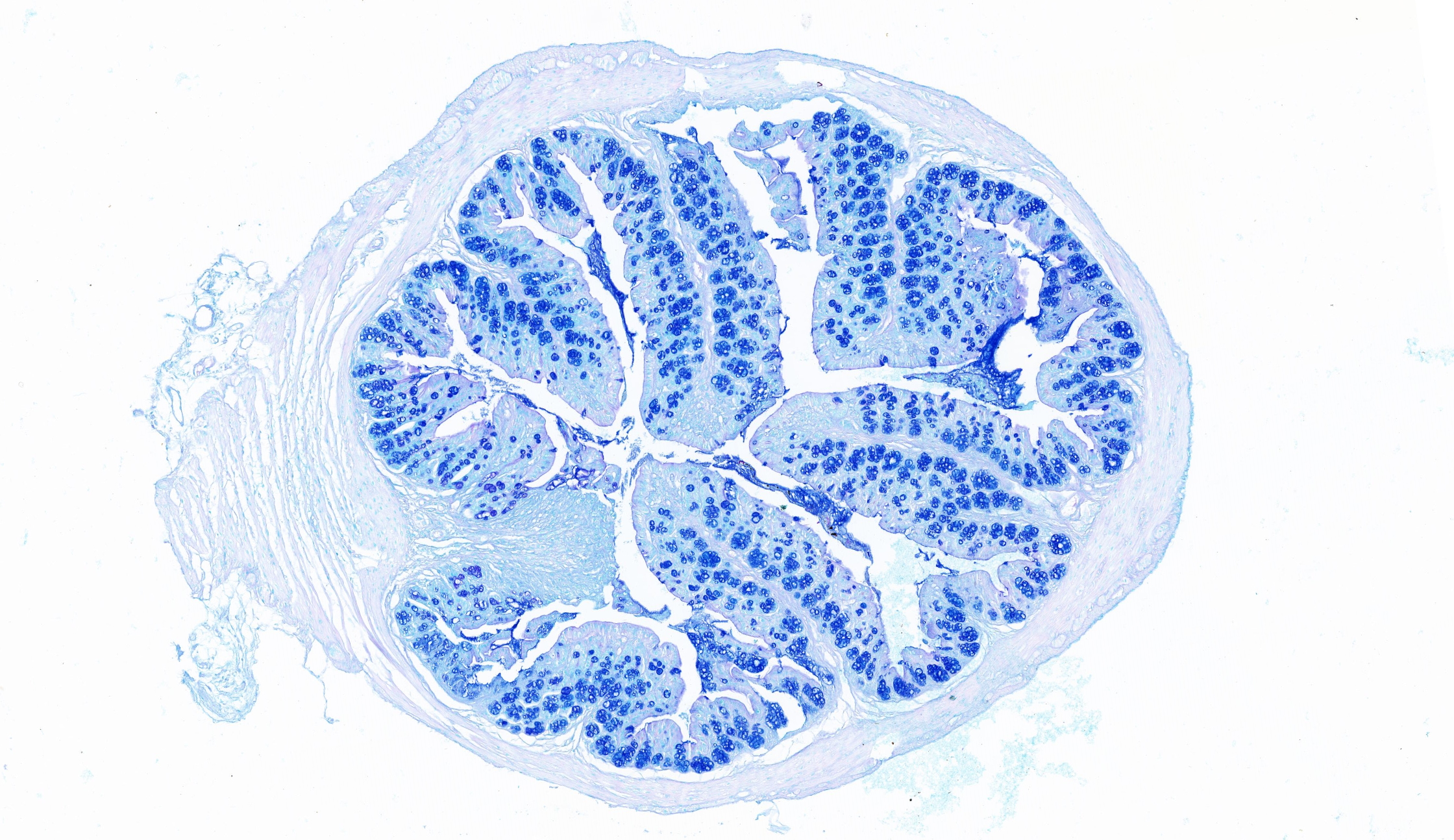

Supplement: Supplementary file 13 [file DataSheet13.zip › fig5-E(AB-PAS Original image)/5-ASA.jpg]

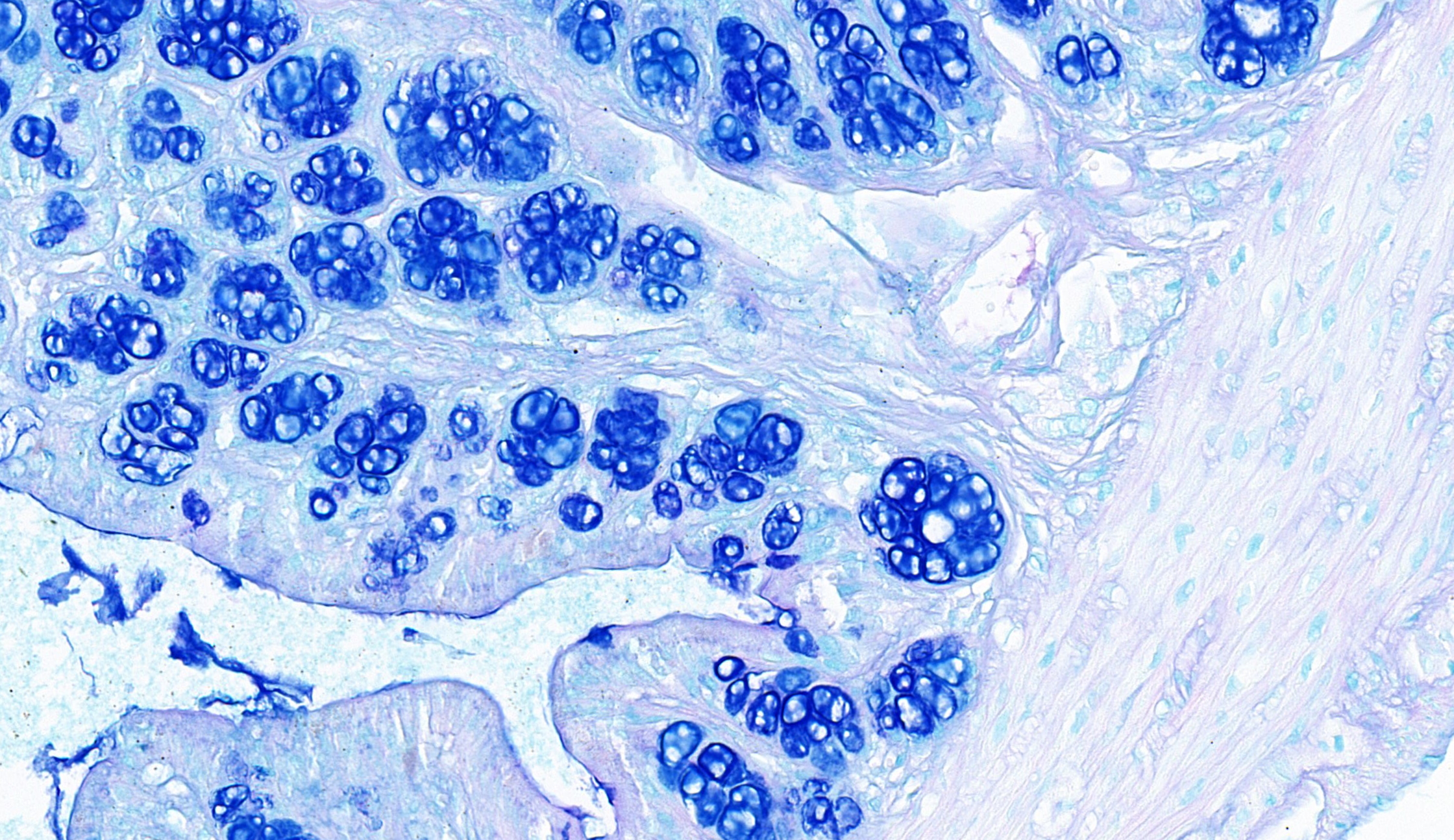

Supplement: Supplementary file 13 [file DataSheet13.zip › fig5-E(AB-PAS Original image)/5-ASA(1).jpg]

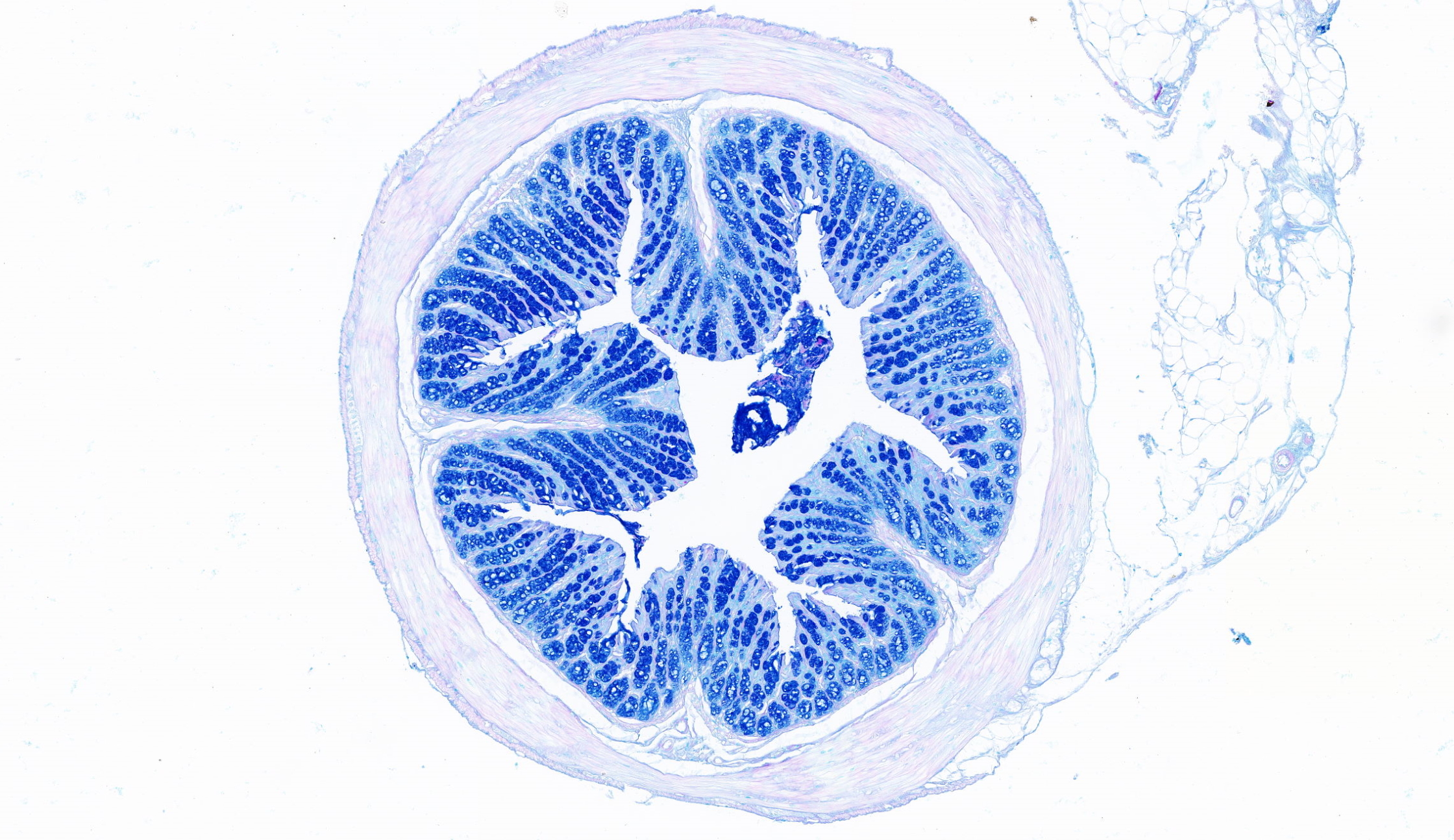

Supplement: Supplementary file 13 [file DataSheet13.zip › fig5-E(AB-PAS Original image)/Control.jpg]

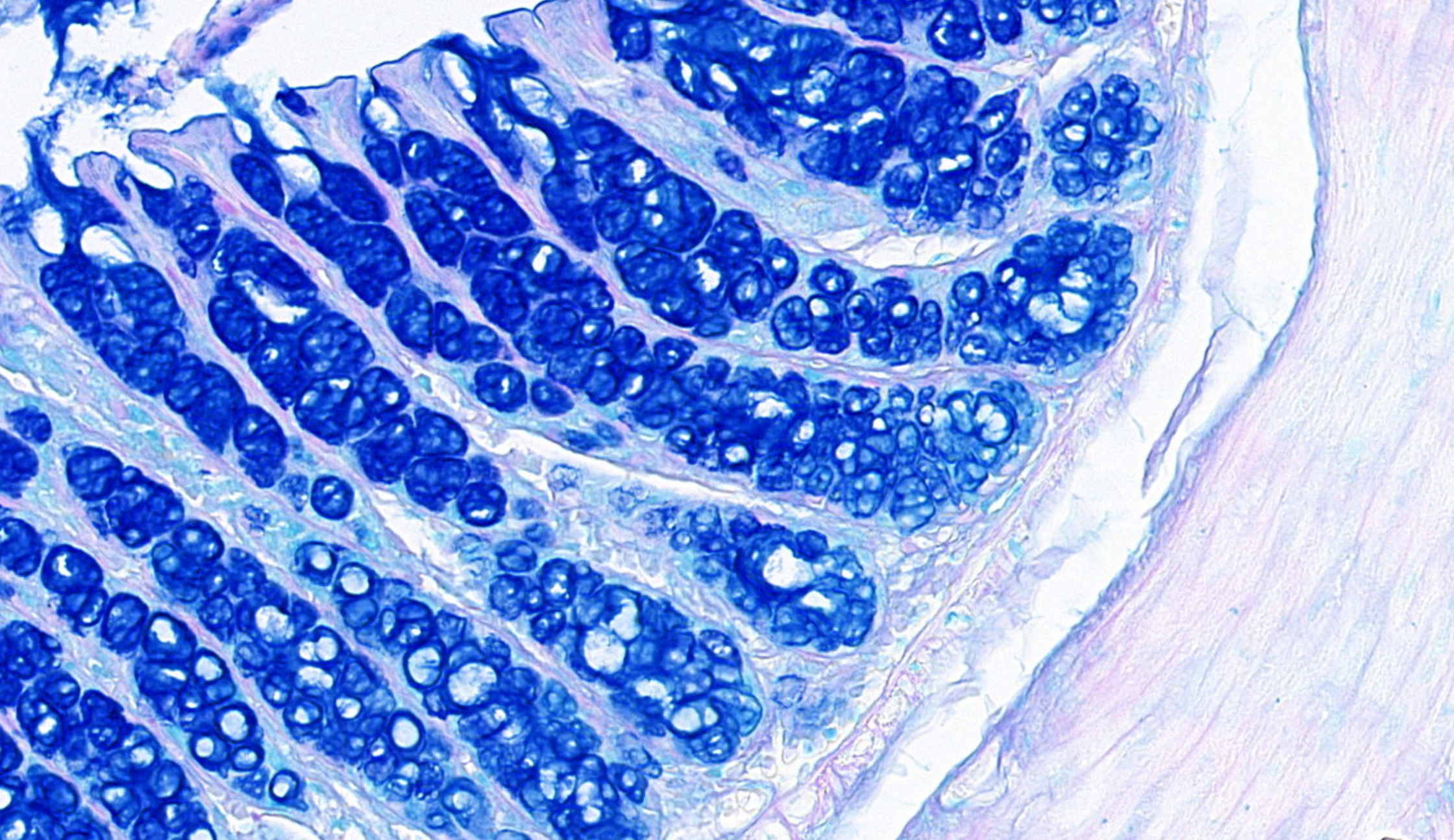

Supplement: Supplementary file 13 [file DataSheet13.zip › fig5-E(AB-PAS Original image)/Control(1).jpg]

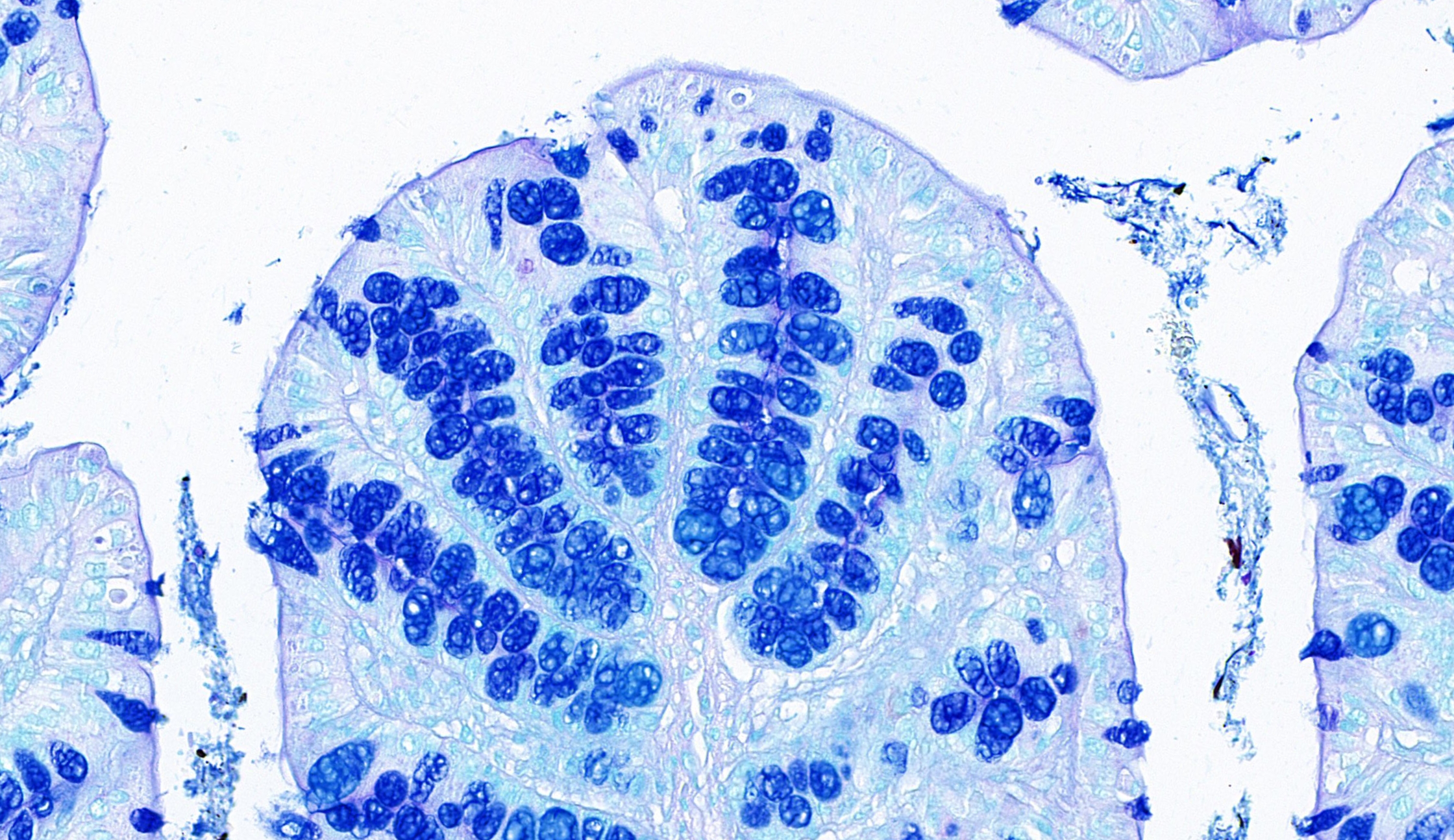

Supplement: Supplementary file 13 [file DataSheet13.zip › fig5-E(AB-PAS Original image)/DGD-D(1).jpg]

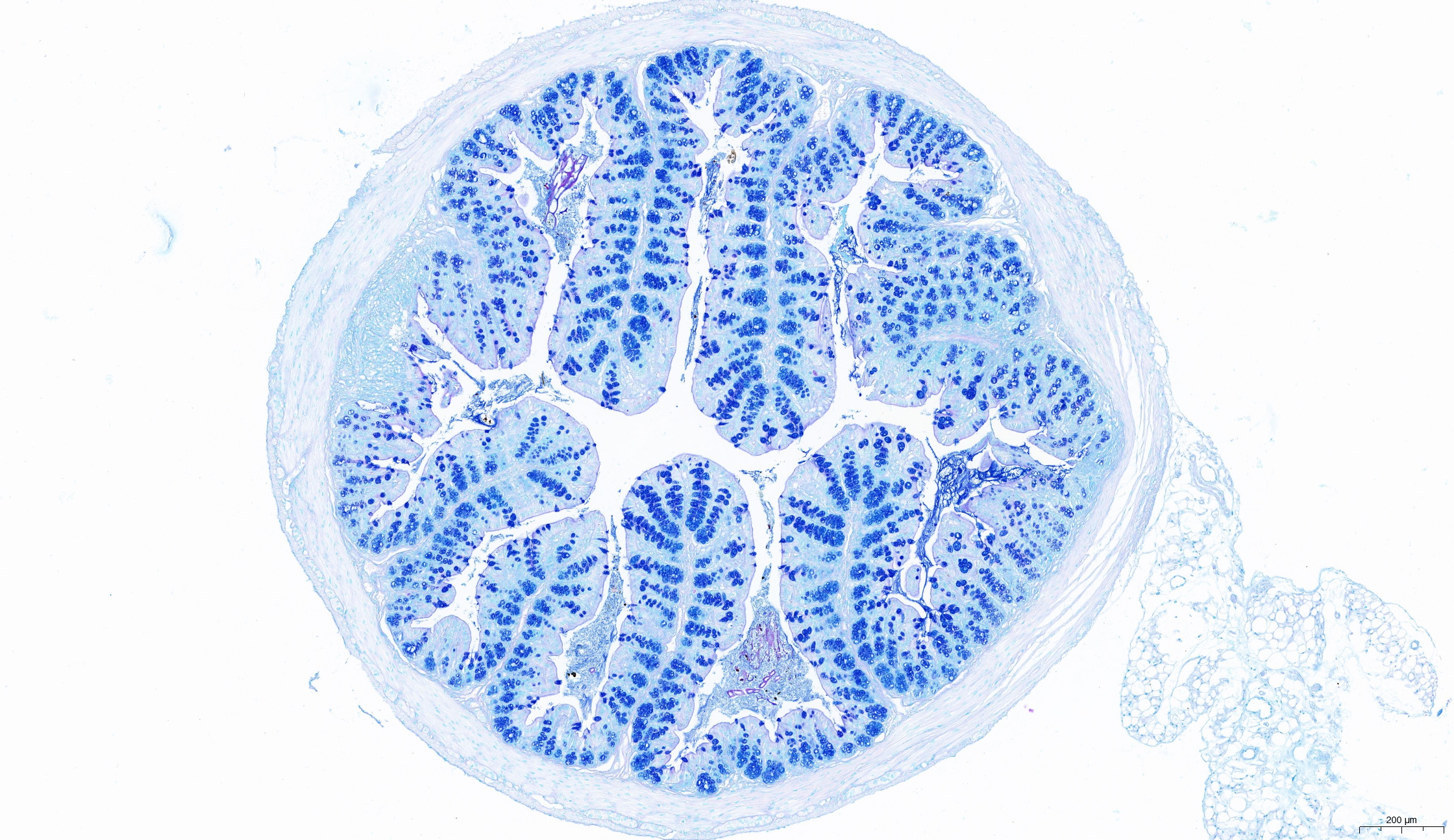

Supplement: Supplementary file 13 [file DataSheet13.zip › fig5-E(AB-PAS Original image)/DGD-D.jpg]

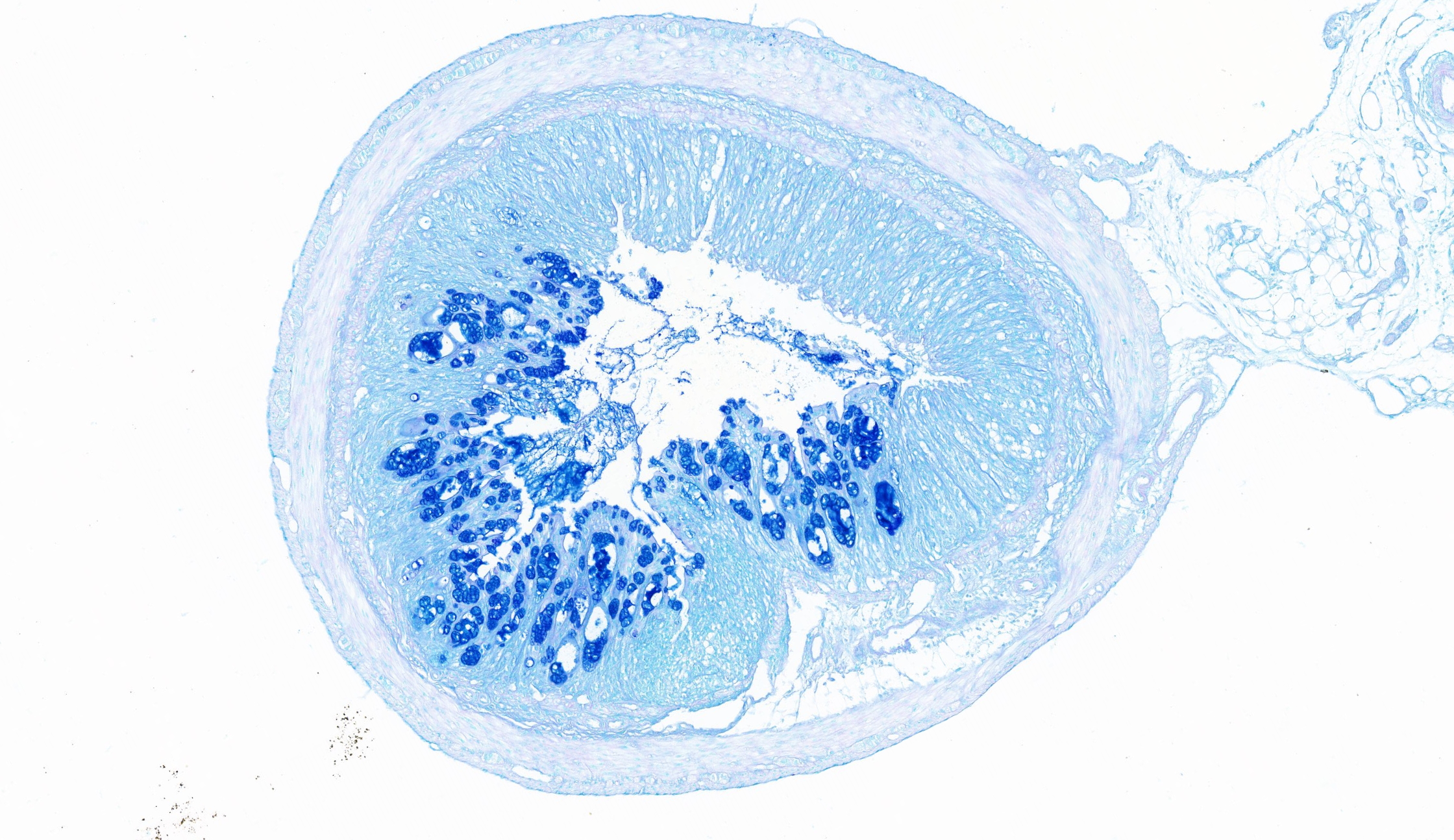

Supplement: Supplementary file 13 [file DataSheet13.zip › fig5-E(AB-PAS Original image)/DSS.jpg]

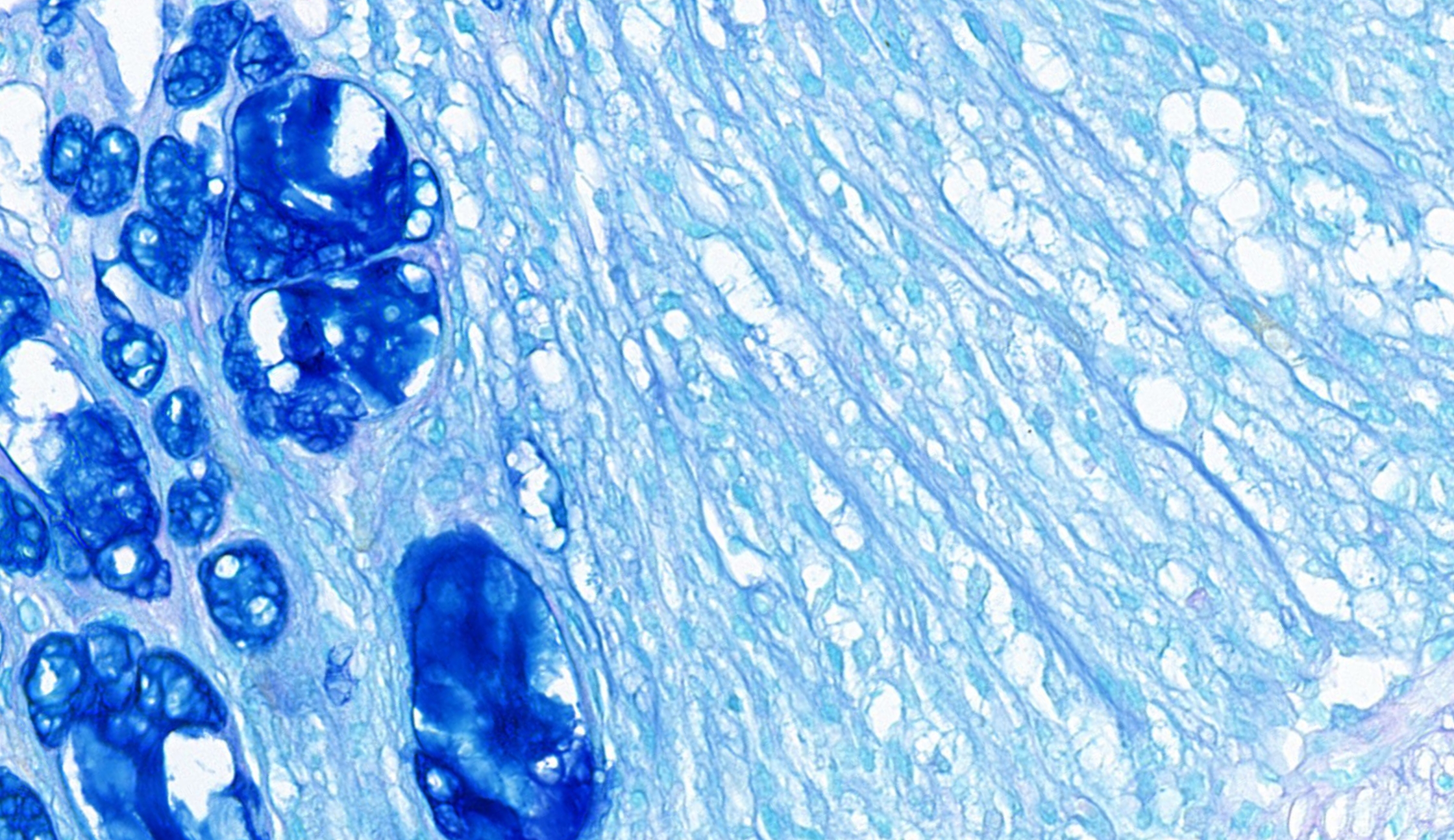

Supplement: Supplementary file 13 [file DataSheet13.zip › fig5-E(AB-PAS Original image)/DSS(1).jpg]
